# Small-Molecule Drugs Approved by the FDA in 2025

**DOI:** 10.3390/molecules31193447

**Published:** 2026-09-27

**Authors:** Xiaoyue Wang, Ying Wang, Rui Luo, Donghai Niu, Liyan Yang, Zhonglei Wang

**Affiliations:** 1 Key Laboratory of Green Natural Products and Pharmaceutical Intermediates in Colleges and Universities of Shandong Province, School of Chemistry and Chemical Engineering, Center for Nanomedicine, Qufu Normal University, Qufu 273165, China; 2 School of Physics and Physical Engineering, Qufu Normal University, Qufu 273165, China; 3 School of Chemistry & Chemical Engineering and Environmental Engineering, Weifang University, Weifang 261061, China; 4 Beijing National Laboratory for Molecular Sciences, Institute of Chemistry, Chinese Academy of Sciences, Beijing 100190, China; 5 Key Laboratory of Bioorganic Phosphorus, School of Pharmaceutical Sciences, Chemistry & Chemical Biology (Ministry of Education), Tsinghua University, Beijing 100084, China

**Keywords:** innovative advances, small-molecule drugs, FDA, first-in-class, oncology, breakthrough therapy

## Abstract

In today’s pharmaceutical sector, innovation is not only the core driver of industry upgrading, but also closely tied to patient health and well-being. A drug’s innovative mechanism of action, physiological effects, chemical structure, and indications collectively reflect both its clinical therapeutic value and the novelty of underlying basic research. This article presents the innovative advances of 31 small-molecule drugs (accounting for 67.4%) approved by the U.S. Food and Drug Administration (FDA) in 2025. These therapeutics span multiple therapeutic areas, including oncology, autoimmune diseases, infectious diseases, cardiovascular disorders, and metabolic conditions, and target a diverse array of molecular targets such as mitogen-activated protein kinase kinases 1 and 2 (MEK1/2), colony-stimulating factor 1 receptor, protein tyrosine kinase Tec, and Bruton tyrosine kinase, among others. Among these approved small-molecule drugs, 13 are designated as “first-in-class” therapies, reflecting the continuous innovation in the field of new drug development. Notably, many of these drugs (e.g., Journavx, Brinsupri, Jascayd, and Kygevvi) have also been granted breakthrough therapy designation, which underscores that they not only feature novel mechanisms of action, but also deliver clinically meaningful efficacy advantages over prior therapeutic options. Notably, in 2025, the FDA approved sunvozertinib, a novel small-molecule drug independently developed by Dizal (Jiangsu, China) Pharmaceutical Co., Ltd., for locally advanced or metastatic non-small cell lung cancer (NSCLC) with epidermal growth factor receptor (EGFR) exon 20 insertion mutations, contributing Chinese wisdom and solutions to the transformative evolution of global drug development paradigms. Cross-technology integration, long-acting drug delivery, and precision medicine stand as core industrial trends, enabling innovative drugs to boost therapeutic efficacy, mitigate toxic side effects and refine patients’ medical experience, offering patients expanded regimens for improved survival outcomes. With the development of first-in-class drugs, therapies targeting previously undruggable targets—or those inaccessible via conventional approaches—are entering clinical practice, creating major opportunities for novel, mechanism-based drug discovery. This article comprehensively analyzes the mechanisms of action, synthesis methods, and clinical trials of these drugs chronologically, and delineates emerging trends in novel drug development.

## 1. Introduction

In 2025, the U.S. Food and Drug Administration (FDA) approved a total of 46 new drugs, representing a slight decrease from the 50 approvals in 2024 and falling below the 55 approvals in 2023 [1,2,3]. Among these 46 approved drugs, small molecule agents accounted for 31 (67%), spanning multiple therapeutic areas, with oncology remaining the largest category [4,5,6,7,8,9,10]. Notably, 15 additional FDA-approved non-small-molecule therapeutics (33%)—comprising 12 biologics (seven monoclonal antibodies: Clesrovimab, Depemokimab, Garadacimab, Narsoplimab, Nipocalimab, Penpulimab, and Sibeprenlimab; two antibody–drug conjugates: Datopotamab deruxtecan and Telisotuzumab vedotin; one bispecific antibody: Linvoseltamab; one adnectin-based scaffold protein: Lerodalcibep; and one enzyme-enabled antibody formulation: pembrolizumab with berahyaluronidase alfa) and three oligonucleotide therapeutics (Fitusiran, Plozasiran, and Donidalorsen)—represent a significant advancement in modality diversification this year, complementing—not replacing—small-molecule medicines. This trend does not signify the obsolescence of small molecules; rather, it represents a strategic diversification of the therapeutic arsenal—enabling precision intervention at multiple biological levels: genomic, transcriptomic, proteomic, and cellular.

In 2025, newly approved drugs by the U.S. FDA continued the prevailing trend of “small-molecule dominance”. Building on our earlier article [3], this article highlights the latest research advances of these 31 small molecule drugs (Table 1), including Grafapex (**1**), Journavx (**2**), Gomekli (**3**), Romvimza (**4**), Blujepa (**5**), Vanrafia (**6**), Avmapki Fakzynja Co-Pack (**7**), Tryptyr (**8**), Ibtrozi (**9**), Zegfrovy (**10**), Ekterly (**11**), Anzupgo (**12**), Sephience (**13**), Vizz (**14**), Modeyso (**15**), Hernexeos (**16**), Brinsupri (**17**), Wayrilz (**18**), Forzinity (**19**), Palsonify (**20**), Inluriyo (**21**), Rhapsido (**22**), Jascayd (**23**), Lynkuet (**24**), Kygevvi (**25**), Komzifti (**26**), Hyrnuo (**27**), Cardamyst (**28**), Nuzolvence (**29**), Myqorzo (**30**), and Nereus (**31**). These drugs exhibit a broad spectrum of biological activities, primarily serving as anticancer agents (**3**, **7**, **9**, **10**, **16**, **21**, **27**), gene targeted drugs (**4**, **6**, **11**, **13**, **15**, **18**, **19**, **20**, **25**), and cardiovascular and cerebrovascular drugs (**1**, **24**, **26**, **28**, **30**). Other therapeutic indications include anti-infective drugs (**5**, **29**), pulmonary drugs (**17**, **23**), ophthalmic drugs (**8**, **14**), dermatological drugs (**12**, **22**), and neurological drugs (**2**, **31**). Among the approved small molecule drugs, Journavx, Blujepa, Avmapki Fakzynja, Tryptyr, Modeyso, Brinsupri, Wayrilz, Forzinity, Rhapsido, Jascayd, Lynkuet, Kygevvi, and Nuzolvence are classified as “first-in-class” therapies (Table 1) [10]. These agents not only possess unique mechanisms of action, but also demonstrate significantly improved efficacy compared with previous therapeutic options. The **31** small-molecule drugs approved by the U.S. FDA in 2025 embody an innovative landscape characterized by diversification, precision and groundbreaking advancements. Through structural optimization and enhanced target selectivity, these small-molecule therapeutics effectively address drug resistance in chronic diseases and oncology [11,12]. Novel therapeutic modalities including peptides [13] have matured rapidly, delivering major breakthroughs for rare diseases and refractory malignancies while filling substantial unmet clinical gaps. Moving beyond homogeneous incremental modifications, industrial research and development (R&D) has pivoted toward novel molecular targets, first-in-class mechanisms, and unmet medical needs, yielding a marked uplift in overall innovation quality [9]. Precision medicine, long-acting drug delivery, and cross-technology integration stand as core industrial trends, enabling innovative drugs to boost therapeutic efficacy, mitigate toxic side effects, and refine patients’ medical experience [14]. Sustained advances in digital healthcare, targeted delivery systems and emerging biotechnologies will facilitate progressive clinical management of intractable and rare diseases, offering patients expanded regimens for improved survival outcomes. This article provides a comprehensive analysis of the mechanisms of action, structural characteristics, synthetic methods, and clinical development status, safety, and efficacy data of these drugs in chronological order, and delineates emerging trends in novel drug development. Moreover, this study comprehensively analyzes current trends in the global drug discovery landscape, including the increasing integration of artificial intelligence in target identification. As such, it constitutes a rigorously curated and highly valuable reference for medicinal chemists, pharmacologists, and early-stage drug developers engaged in target validation, lead optimization, and candidate selection.

## 2. Grafapex (Treosulfan)

On 22 January 2025, the U.S. FDA approved Grafapex (treosulfan), developed by Medexus Pharmaceuticals in combination with fludarabine as a conditioning regimen for allogeneic hematopoietic stem-cell transplantation (allo-HSCT) in adult and pediatric patients aged 1 year and older with acute myeloid leukemia (AML) or myelodysplastic syndrome (MDS) [15,16]. Treosulfan is an alkylating agent administered to patients as a conditioning therapy prior to allogeneic hematopoietic stem cell transplantation [17]. It is used to ablate the patient’s bone marrow, creating space for the transplanted donor stem cells, which subsequently generate healthy blood cells [18]. When evaluating its pharmacokinetic profile, treosulfan bypasses liver-based bioactivation entirely. Because of its characteristic molecular architecture, the agent undergoes spontaneous hydrolysis within bodily physiological environments, generating bioactive species like single-function epoxide intermediates and L-diepoxybutane. These epoxide moieties attach alkyl groups to the N7 position of guanine units in DNA, prompting the creation of interstrand DNA cross-links. This mechanism delivers anti-neoplastic activity and selectively eradicates malignant cell populations, which in turn improves the successful implantation of donor stem cells [19,20]. Treosulfan features a carbon sugar alcohol backbone, wherein four hydroxyl groups are methylated, the hydroxyl group at the C2 position is substituted with a thiol group, and sulfate ester moieties are attached at the C1 and C4 positions. This structural design confers chemical stability under neutral pH conditions while enabling acid-catalyzed hydrolysis in mildly acidic environments: initial cleavage of the sulfate ester groups yields the monoepoxide intermediate, which subsequently undergoes spontaneous intramolecular cyclization to generate the electrophilic diepoxide metabolite (DEB). Critically, this activation pathway proceeds without enzymatic catalysis. DEB—the ultimate biologically active form of treosulfan (NSC 39069)—covalently alkylates nucleophilic sites in DNA, predominantly at the N7 position of guanine residues, thereby inducing both intrastrand and interstrand crosslinks. Such crosslinks distort the DNA double helix, impeding the function of DNA polymerases and topoisomerases, ultimately triggering S-phase cell cycle arrest and the activation of apoptotic pathways. Compared with conventional nitrogen mustard-based alkylating agents, treosulfan’s diepoxide structure exhibits markedly enhanced DNA crosslinking efficiency, with IC_50_ values reaching micromolar to nanomolar ranges across multiple tumor cell lines.

Synthesis of treosulfan (Figure 1) adopts dimethyl L-tartrate **1.1** as the sole chiral starting material [21]. Condensation of compound **1.1** with p-anisaldehyde dimethyl acetal **1.2** in toluene under p-toluenesulfonic acid catalysis at 110 °C for 2 h affords the dioxolane diester intermediate **1.3** with an isolated yield of 92%. Subsequent ester reduction of **1.3** using a sodium borohydride–iodine system in THF–water over 2 h at 15 °C produces the diol intermediate **1.4** in 84% yield. Double mesylation of diol **1.4** with methanesulfonyl chloride **1.5** in dichloromethane at 0 °C for 3 h, with pyridine as the acid scavenger, generates the disulfonate intermediate **1.6** in 79% yield. Final ketal hydrolysis of **1.6** by aqueous hydrochloric acid in methanol over 8 h at 10 °C removes the protecting group and cleaves the dioxolane ring, yielding high purity treosulfan (**1**) with a yield of 68%. The chiral configuration remains unchanged during the whole synthetic process.

The approval of treosulfan plus fludarabine is based on an open-label, randomized, non-inferiority Phase 3 trial (NCT00822393) involving 476 patients aged 18–70 years with AML or MDS undergoing allogeneic HSCT [22]. Participants were randomly assigned in a 1:1 ratio to receive either intravenous 10 g/m^2^ treosulfan daily for 3 days (days −4 to −2) plus 30 mg/m^2^ fludarabine daily for 5 days (days −6 to −2), or 0–8 mg/kg busulfan on days −4 and −3 plus fludarabine. The primary efficacy endpoint was event-free survival 2 years after HSCT, with a non-inferiority margin of a hazard ratio (HR) of 1.3. Results indicated that 2-year event-free survival was 64.0% (95% CI 56.0–70.9) in the treosulfan group compared to 50.4% (42.8–57.5) in the busulfan group, yielding an HR of 0.65 (95% CI 0.47–0.90; *p* < 0.0001 for non-inferiority, *p* = 0.0051 for superiority). Among the patients with baseline comorbidities, outcomes also favored the treosulfan group. For subjects with an HSCT-specific comorbidity index > 2, event-free survival was numerically higher in the treosulfan arm. Common adverse events associated with treosulfan included abnormal blood chemistry results (15%) and gastrointestinal disorders (11%). Serious adverse events were reported in 18% of patients in the treosulfan group, with causes generally transplantation related.

## 3. Journavx (Suzetrigine)

Studies show that most patients experience moderate-to-severe pain postoperatively. Commonly used analgesics (such as acetaminophen and opioids) lack target specificity due to multi-target mechanisms, resulting in suboptimal efficacy and frequent adverse effects; while opioids provide potent analgesia, they carry risks of dependence and addiction [23]. Poor acute pain management impairs quality of life and increases healthcare and societal burdens [24,25]. Thus, developing novel non-opioid, target-specific analgesics without potential addiction is urgently needed [26]. The perception of pain in the human body is a complex physiological process. When the body is subjected to harmful stimuli, peripheral pain-sensing neurons (nociceptors) are activated, generating action potential. These action potentials are conducted along nerve fibers to the spinal cord, and then through the spinal cord to the brain, where they are ultimately perceived as pain [27]. During this process, voltage-gated sodium channels play a crucial role. Voltage-gated sodium channels are the key channels through which neurons transmit action potential. In humans, there are nine different subtypes (Nav1.1–Nav1.9), among which Nav1.8 is selectively expressed in peripheral pain-sensing neurons and is responsible for transmitting pain signals [28,29]. On 30 January 2025, the U.S. FDA approved Suzetrigine (VX-548), a first-in-class selective NaV1.8 inhibitor developed by Vertex Pharmaceuticals, for the treatment of moderate to severe acute pain in adults, marketed under the brand name Journavx [30,31,32]. This approval marks the first novel class of analgesic agents to be authorized in over two decades, and it is the world’s first approved non-opioid oral analgesic for this indication. Journavx is a selective NaV1.8 inhibitor [33,34,35]. By selectively inhibiting NaV1.8 channels, this drug blocks the conduction of pain signals at nerve endings, thereby alleviating pain [30]. Unlike opioids, Journavx does not act on the brain and thus does not trigger the brain’s reward center, avoiding the risk of addiction.

Developed via scaffold hopping of the early candidate VX-150 by Vertex Pharmaceuticals, suzetrigine (VX-548) is a marketed analgesic agent whose structural design was disclosed in Patent WO2021/113627A1. Abandoning the phosphate prodrug strategy adopted by VX-150, this drug utilizes a phenyltetrahydrofuran scaffold instead of the diphenyl ether scaffold to enhance binding affinity toward target channels (Figure 2). Substituents of fluorine and trifluoromethoxy groups are introduced to improve metabolic stability. Possessing physicochemical properties that satisfy four criteria of Lipinski’s Rule of Five, suzetrigine exhibits favorable oral bioavailability with limited central nervous system penetration. It delivers potent inhibitory activity against NaV1.8 currents in human dorsal root ganglia neurons and displays over 31,000-fold selectivity versus other sodium channel subtypes [36,37]. VX-150 was first documented in Patent WO2015/089361A1. Although its lead compound VX-150P featured robust and selective NaV1.8 inhibitory activity, it suffered from extremely poor aqueous solubility. Derivatization into phosphate prodrug VX-150 effectively ameliorated solubility. Despite promising therapeutic efficacy observed in Phase II clinical trials, further clinical development of VX-150 was discontinued owing to adverse reactions including dizziness and headache [38,39].

The synthesis of suzetrigine (Figure 3) begins with 2-(3,4-difluoro-2-methoxyphenyl)acetic acid (**2.1**) [40,41]. Activation of **2.1** with 1,1′-carbonyldiimidazole (CDI) in acetonitrile, followed by intramolecular condensation, affords unsaturated γ-lactone **2.2** in 85% yield. Catalytic hydrogenation of **2.2** over palladium on carbon (Pd/C) in isopropanol furnishes saturated lactone **2.3** in 95% yield. Subsequent reduction of **2.3** with diisobutylaluminum hydride (DIBAL-H) in toluene delivers cyclic hemiketal **2.4** in 99.7% yield. Acylation of **2.4** with 4-nitrobenzoyl chloride yields nitrobenzoate ester **2.5**, which undergoes cyanosilylation with trimethylsilyl cyanide (TMSCN) and boron trifluoride diethyl etherate (BF_3_·Et_2_O) to afford nitrile **2.6**. Enantioselective resolution of **2.6** using quinine provides chiral carboxylic acid **2.7** in 70% yield. Treatment of **2.7** with oxalyl chloride generates acyl chloride **2.8**, which is coupled with methyl 4-aminopyridine-2-carboxylate to give amide **2.9** in 90% yield. Finally, ammonolysis of **2.9** in a solution of ammonia in methanol affords suzetrigine (**2**) in 94% yield.

The U.S. FDA approval of Journavx was based on two randomized, double-blind, placebo- and active-controlled clinical trials of acute postoperative pain. One study enrolled 1073 patients undergoing bunionectomy (NCT05553366), and the other enrolled 1118 patients undergoing abdominoplasty (NCT05558410) [42,43]. Analyses from the Phase 3 trial in abdominoplasty patients showed that Journavx met the primary endpoint: the time-weighted sum of pain intensity difference over 48 h (SPID48) was significantly reduced in the Journavx group compared with the placebo, with a mean difference of 48.4 (95% CI: 33.6–63.1; *p* < 0.0001). In addition, patient Numerical Pain Rating Scale (NPRS) scores at 48 h postoperatively were also significantly reduced from the baseline. In bunionectomy patients, the therapy also achieved the primary endpoint, with a mean SPID48 difference of 29.3 (95% CI: 14. 0–44.6; *p* = 0.0002). The most common adverse reactions in patients treated with Journavx in the studies were pruritus, muscle spasms, elevated CPK levels, and rash. In summary, this product is the first novel analgesic approved in over two decades. Its approval marks a paradigm-shifting advancement in medicine—ushering in a transformative revolution in pain management. The drug not only breaks the long-standing dominance of opioid-based therapies in the analgesic landscape, but also offers a new, highly effective, and non-addictive treatment option for hundreds of millions of patients worldwide suffering from chronic pain thanks to its distinct mechanism of action. Dubbed a “paradigm shift in pain medicine”, it provides renewed hope to the global healthcare community, which has been grappling with the ongoing opioid addiction crisis. Emerging evidence further supports its potential in neuropathic pain, positioning it as a promising component of multimodal analgesia. Its innovative target mechanism offers a new approach to overcoming the opioid crisis. However, clinical translation faces two key challenges: a lack of safety data in special populations (e.g., pregnancy and lactation) and uncertainty regarding long-term risks—constituting critical bottlenecks in evaluating its efficacy and safety within multimodal analgesia and during pregnancy.

## 4. Gomekli (Mirdametinib)

Type 1 neurofibromatosis (NF1) is a genetic disorder, with patients facing a 30–50% lifetime risk of developing plexiform neurofibromas (PN) [44]. PNs exhibit invasive growth along peripheral nerve sheaths and may result in severe disfigurement, pain, functional impairment, and even malignant transformation into malignant peripheral nerve sheath tumors [45]. Owing to the highly infiltrative nature of these tumors, surgical resection is challenging, and effective long-term treatment options have been limited for affected patients, highlighting an urgent medical need for innovative therapies to improve clinical outcomes [44]. On 11 February 2025, the U.S. FDA approved Gomekli (Mirdametinib), a MEK inhibitor developed by biopharmaceutical company SpringWorks Therapeutics, for the treatment of adult and pediatric patients aged 2 years and older with NF1 who have symptomatic, unresectable plexiform neurofibromas [46]. Notably, Gomekli is the first and only approved therapy for NF1-associated plexiform neurofibromas (NF1-PN) indicated for use in both adult and pediatric populations.

The synthesis of mirdametinib (Figure 4) proceeds via a convergent route [47]. Initially, 2-fluoro-4-iodoaniline (**3.1**) is coupled with 2,3,4-trifluorobenzoic acid (**3.2**) through lithium amide (LiNH_2_)-mediated condensation, followed by methanol esterification to afford methyl ester **3.3** in 62% yield. Subsequent hydrolysis with LiOH furnishes carboxylic acid **3.4** in 96% yield. In parallel, (S)-2,2-dimethyl-1,3-dioxolane-4-methanol (**3.5**) undergoes a Mitsunobu reaction with N-hydroxyphthalimide (**3.6**) using diethyl azodicarboxylate (DEAD) and triphenylphosphine (PPh_3_) to yield intermediate **3.7** in 86% yield. Cleavage of the phthalimide protecting group with hydrazine affords hydroxylamine **3.8** in 75% yield. Coupling of acid **3.4** and hydroxylamine **3.8**—mediated by EDCI, HOBt, and DIPEA in DMF—gives protected amide **3.9** in 67% yield. Finally, acidic deprotection of the acetonide moiety in **3.9** using p-toluenesulfonic acid (p-TsOH) in aqueous methanol delivers mirdametinib (**3**) in 82% yield.

The approval of Gomekli (mirdametinib) was based on the results of the Phase IIb ReNeu trial (NCT03962543), which enrolled 114 patients with NF1-PN aged ≥ 2 years, including 58 adults and 56 children [48]. Data demonstrated that Gomekli met the primary endpoint of ORR in both adult and pediatric cohorts, with ORRs of 41% and 52%, respectively. Patients receiving treatment achieved significant and durable tumor volume reduction; the median best percentage change in tumor volume was −41% for adults and −42% for children. Furthermore, the duration of response (DOR) was at least 12 months in 88% of adult patients and 90% of pediatric patients, and at least 24 months in 50% of adult patients and 48% of pediatric patients. Sustained improvements from the baseline in pain levels and quality of life were also observed across multiple assessment tools. In terms of safety, Gomekli exhibited a manageable safety profile and tolerability. The most common adverse events (AEs) occurring in >25% of adult patients included rash, diarrhea, nausea, musculoskeletal pain, vomiting, and fatigue. In contrast, the most common AEs (≥25%) in pediatric patients were rash, diarrhea, musculoskeletal pain, abdominal pain, vomiting, headache, paronychia, left ventricular dysfunction, and nausea. Gomekli had previously been granted orphan drug designation and Fast Track designation by the FDA. Following this approval, SpringWorks received a Rare Pediatric Disease Priority Review Voucher, which may be used to accelerate the development of other investigational therapies. The European Medicines Agency (EMA) has validated its Marketing Authorization Application, which is currently under review, potentially expanding patient access to the therapy in the future.

## 5. Romvimza (Vimseltinib)

Tenosynovial giant cell tumor (TGCT) is a rare, benign yet locally aggressive soft tissue tumor that typically arises in the synovium, bursae, or tendon sheaths of joints [49]. Patients are commonly present with joint pain, swelling, stiffness, and limited mobility. Traditional treatment is primarily surgical, but for diffuse or recurrent cases, surgery may result in significant functional impairment or even disability [50]. On 14 February 2025, the U.S. FDA approved Romvimza (vimseltinib) for the treatment of adult patients with symptomatic TGCT for whom surgical resection would likely lead to worsening functional limitations or severe morbidity [51,52]. The drug is currently under review by the EMA. Vimseltinib is a small-molecule CSF1R tyrosine kinase inhibitor. In TGCT, overactivation of the CSF1R signaling pathway leads to abnormal proliferation of macrophages in synovial tissue, resulting in tumor formation. By blocking autophosphorylation and signaling of CSF1R, vimseltinib inhibits the survival and accumulation of these abnormal cells, interrupting the pathological process at its source [53]. Clinical studies have demonstrated that this mechanism can significantly reduce tumor volume, improve joint function, and alleviate pain [54].

Leveraging prior knowledge of key drug–receptor interactions conferring high affinity and selectivity for CSF1R, Caldwell et al. [55] initiated their medicinal chemistry campaign with the 5-(pyridin-4-yloxy)pyridine core compound **V-2** (Figure 5). They systematically evaluated a diverse array of heterocyclic substituents at the 2-position, identifying the dihydropyrimidinone derivative **V-2** as an early lead compound exhibiting promising CSF1R potency and selectivity. Building upon this lead, a comprehensive structure–activity relationship (SAR) optimization effort ultimately yielded vimseltinib (DCC-3014).

Synthesis of vimseltinib (Figure 6) commences with 2-(methylthio)pyrimidin-4(3H)-one (**4.1**) [55]. N-Methylation of **4.1** using lithium hexamethyldisilazide (LiHMDS) and methyl iodide (MeI) affords N-methylpyrimidinone **4.2** in 62% yield. Subsequent bromination of **4.2** proceeds smoothly to deliver bromopyrimidine **4.3** in 97% yield. Palladium-catalyzed borylation of **4.3** with bis(pinacolato)diboron furnishes boronate ester **4.4**. Concurrently, Suzuki coupling of 6-iodo-2-methylpyridin-3-yl 3-chloropyridin-4-yl ether (**4.5**) yields diaryl ether pyrimidinone **4.6** in 67% yield. A second Suzuki cross-coupling reaction involving **4.6** constructs advanced intermediate **4.7** in 34% yield. Finally, treatment of **4.7** with isopropylamine (**4.8**) at 100 °C affects nucleophilic displacement of the methylthio group, affording vimseltinib (**4**) in 59% yield. Notably, this synthetic route employs highly toxic liquid bromine (Br_2_), which exhibits strong corrosivity toward process equipment and poses significant challenges for safe transportation and handling—thereby impeding scalability for large-scale manufacturing.

The approval of Romvimza was based on the randomized, double-blind, placebo-controlled Phase 3 MOTION study (NCT05059262) that evaluated efficacy [56]. Eligible patients had a confirmed diagnosis of tenosynovial giant cell tumor with measurable disease and at least one lesion with a minimum size of 2 cm. A total of 123 patients were randomized to receive either the placebo (n = 40) or Romvimza 30 mg (n = 83) twice weekly for 24 weeks. Randomization was stratified by tumor location (lower extremity vs. all other sites) and region (U.S. vs. non-U.S.). The primary efficacy endpoint was the ORR assessed by blind independent radiologic review (IRR) per Response Evaluation Criteria in Solid Tumors (RECIST) v1.1 at Week 25. Other efficacy outcomes measured at Week 25 included ORR assessed by Tumor Volume Score (TVS), mean change from baseline in affected joint range of motion as measured by goniometry, change from baseline in Patient-Reported Outcomes Measurement Information System—Physical Function (PROMIS-PF) 15-item score (upper and lower extremity items), and the proportion of patients with at least a 30% improvement in the average Brief Pain Inventory Worst Pain Numeric Rating Scale (NRS) score without a 30% or greater increase in opioid analgesic use. Results showed that patients randomized to Romvimza had a statistically significant improvement in ORR compared with the placebo group, with an ORR of 40% and a complete response rate of 5%. No responses were observed in the placebo group. Median DOR was not reached in the Romvimza group; with an additional 6 months of follow-up, 28 responders (85%) had a DOR ≥ 6 months and 19 (58%) had a DOR ≥ 9 months. Furthermore, statistically significant improvements in quality-of-life measures, including range of motion, patient-reported physical function, and pain, were observed in the Romvimza group relative to the placebo group, supporting the primary endpoint. The ORR assessed by TVS was 67% of patients randomized to Romvimza. Romvimza was well-tolerated in the trial. The most common adverse reactions (≥20%) included laboratory abnormalities (increased aspartate aminotransferase), periorbital edema, fatigue, rash, increased cholesterol, peripheral edema, facial edema, neutropenia, leukopenia, pruritus, and increased alanine aminotransferase. No evidence of cholestatic hepatotoxicity or drug-induced liver injury was identified by investigators in the study, and the labeling does not carry any boxed warnings or require a restricted Risk Evaluation and Mitigation Strategy program.

## 6. Blujepa (Gepotidacin)

Gonorrhea is a common sexually transmitted infection caused by Neisseria gonorrhoeae that can affect both males and females [57]. If left untreated or inadequately treated, it can lead to infertility and other sexual and reproductive health complications. According to the U.S. Centers for Disease Control and Prevention, more than 600,000 cases of gonorrhea were reported in the U.S. in 2023, making it the second most common sexually transmitted infection in the country [58]. Currently, no preventive vaccine for gonorrhea is approved in the United States, and the standard of care is antibiotic injection. Given the ability of Neisseria gonorrhoeae to develop resistance to the currently available options, including standard-of-care treatments, expanding the range of effective oral therapies has become important [59]. On 25 March 2025, GlaxoSmithKline (GSK) announced that its novel oral antibiotic Blujepa (gepotidacin, GSK2140944) had been approved by the U.S. FDA for the treatment of uncomplicated urinary tract infections (uUTI) in adult females (≥40 kg) and adolescent patients (≥12 years of age, ≥40 kg) caused by the following susceptible microorganisms: Escherichia coli, Klebsiella pneumoniae, Citrobacter freundii complex, Staphylococcus saprophyticus, and Enterococcus faecalis [60]. Blujepa is a first-in-class bactericidal triazaanthracene antibacterial agent that acts by inhibiting bacterial DNA replication. With a novel mechanism of action, it exhibits a well-balanced inhibition of two distinct type II topoisomerases against most pathogens [61]. This confers activity against Neisseria gonorrhoeae and most target uropathogens. Owing to this well-balanced inhibition against most pathogens, single target-specific mutations may not substantially compromise the activity of Blujepa. As the first novel antibiotic for gonorrhea in more than three decades, Blujepa offers a new oral treatment option for patients with gonorrhea in the U.S., where care currently relies on injectable therapies [62].

The synthesis of gepotidacin (Figure 7) proceeds via construction of the tricyclic imidazo[1,2,3-ij]-1,8-naphthyridine-4,9-dione core, followed by sequential installation of the requisite side chains [63,64,65]. The process begins with aromatic nucleophilic substitution between the starting material, 2-chloro-6-methoxy-3-nitropyridine (**5.1**), and 2-amino-1,3-propanediol (**5.2**) in ethanol, affording amino diol **5.3** in 94% yield. Subsequent acetonide protection of **5.3** furnishes compound **5.4** in 92% yield. Reduction of the nitro group in **5.4** via Pd/C-catalyzed hydrogenation yields diamine **5.5**. Alkylation of **5.5** with ethyl bromoacetate affords ester **5.6** in 84% yield; treatment with NaH induces intramolecular cyclization to deliver the bicyclic core **5.7** in 83% yield. Oxidative aromatization of **5.7** using MnO_2_ gives compound **5.8** in 69% yield. Acid-mediated deprotection of the acetonide in **5.8** affords diol **5.9** in 91% yield. Mesylation of **5.9** yields mesylate intermediate **5.10** in 52% yield, which undergoes coupling with N-Boc-4-aminopiperidine (**5.11**) to afford **5.12** in 57% yield. Boc deprotection of **5.12**, followed by chiral chromatographic resolution, provides enantiomerically pure amine **5.13** in 45% yield (over 2 steps). Finally, reductive amination of **5.13** with aldehyde **5.14**, mediated by NaBH(OAc)_3_, delivers gepotidacin (**5**) in 32% yield.

This approval was based on positive results from the pivotal Phase III EAGLE-2 (NCT04020341) and EAGLE-3 (NCT04187144) non-inferiority uUTI trials [66]. EAGLE-2 and EAGLE-3 compared the efficacy and safety of Blujepa (1500 mg orally twice daily for 5 days) versus Nitrofurantoin (100 mg orally twice daily for 5 days) in 1531 and 1605 adult and adolescent female patients with uUTI, respectively. In both trials, participants were planned to be followed for approximately 28 days [66]. The primary endpoint, a stringent composite efficacy measure, was the combined clinical and microbiological response at the test-of-cure (ToC) visit (Days 10–13) in eligible patients with Nitrofurantoin-susceptible uropathogens. Results demonstrated that Blujepa was non-inferior to Nitrofurantoin—one of the current primary standard-of-care options for uUTI—in treatment success across both trials. In EAGLE-2, Blujepa showed non-inferior treatment success: 50.6% (162/320) for Blujepa versus 47.0% (135/287) for Nitrofurantoin (covariate-adjusted treatment difference: 4.3%, 95% CI: −3.6, 12.1). In EAGLE-3, Blujepa demonstrated statistically significant superiority over Nitrofurantoin (one-sided *p* = 0.0003), with treatment success in 58.5% (162/277) of participants versus 43.6% (115/264) in the Nitrofurantoin group (covariate-adjusted treatment difference: 14.6%, 95% CI: 6.4, 22.8). Regarding safety, the safety and tolerability profile of Blujepa in the Phase III EAGLE-2 and EAGLE-3 trials was consistent with that observed in previous studies. The most frequently reported AEs in Blujepa-treated subjects were gastrointestinal (GI) in nature. Diarrhea was the most common (16% of participants), followed by nausea (9%). Among the participants reporting GI AEs in the Blujepa group, the most frequent maximum severity was mild (Grade 1, 69%) and moderate (Grade 2, 28%). Grade 3 GI events occurred in 3% of patients with GI AEs, representing less than 1% of all participants. One drug-related serious adverse event was reported in each treatment group (Blujepa and nitrofurantoin) across both trials.

## 7. Vanrafia (Atrasentan)

IgA nephropathy (IgAN) is a progressive rare kidney disease in which the immune system attacks the kidneys, frequently leading to glomerular inflammation and proteinuria. It is one of the most common autoimmune kidney diseases [67]. Up to 50% of patients with IgAN and persistent proteinuria develop kidney failure within 30 years after diagnosis, often requiring maintenance dialysis and/or kidney transplantation, and responses to treatment can vary [68]. On 2 April 2025, the U.S. FDA granted accelerated approval to Vanrafia (atrasentan), a selective endothelin A receptor antagonist developed by Novartis, for reducing proteinuria in adult patients with primary IgAN at risk of rapid disease progression, typically defined as a urine protein-to-creatinine ratio (UPCR) ≥ 1.5 g/g [69]. Vanrafia is the first and only selective endothelin A receptor antagonist approved to reduce proteinuria in primary IgAN. The drug reduces proteinuria by inhibiting the activity of endothelin A receptors, thereby helping to control disease progression. Vanrafia is a once-daily, nonsteroidal oral therapy that can be seamlessly added to supportive care for IgAN, including renin-angiotensin system (RAS) inhibitors with or without sodium-glucose cotransporter 2 (SGLT2) inhibitors. As a foundational therapy, it does not require a Risk Evaluation and Mitigation Strategy (REMS) program [70]. Atrasentan is a potent endothelin receptor antagonist (ERA) with >1800-fold selectivity for the endothelin A (ETA) receptor over the endothelin B (ETB) receptor (Ki = 63.3 nM). Endothelin-1, the primary effector of the endothelin system, binds ETA receptors—widely expressed in the kidney—to promote vasoconstriction and renal injury, while ETB activation mediates vasodilation. ETA blockade reduces renal fibrosis, inflammation, and proteinuria. Preclinical and clinical evidence supports ETA inhibition as beneficial across multiple kidney diseases, including diabetic nephropathy, IgAN, focal segmental glomerulosclerosis, and oculo-auriculo-renal syndrome. Current standard therapies rely heavily on nonspecific immunosuppressants with significant adverse effects or off-label drugs lacking robust evidence in IgAN. Targeted therapies with distinct mechanisms can help guide optimal, individualized treatment selection [71].

Synthesis of atrasentan (Figure 8) constructs chiral polysubstituted pyrrolidine via linear synthesis. The key Michael addition between nitrostyrene **6.1** and β-ketoester **6.2**, catalyzed by DBU, affords adduct **6.3** in 78% yield [72,73,74]. Subsequent hydrogenation of **6.3** using Raney nickel furnishes pyrroline **6.4**, which is then reduced with NaCNBH_3_ to afford racemic pyrrolidine **6.5**. Epimerization of **6.5** under basic conditions (NaOEt) establishes the required stereochemistry, yielding **6.6**. Boc protection of **6.6** gives **6.7**, followed by NaOH-mediated hydrolysis to afford carboxylic acid **6.8**. Amide coupling of **6.8** with a chiral oxazolidinone auxiliary affords **6.9**, and subsequent Boc deprotection with HCl in dioxane liberates the free amine **6.10**. N-Alkylation of **6.10** with N,N-dibutylbromoacetamide proceeds smoothly over two sequential steps to deliver intermediate **6.11** in 85% overall yield. Finally, cleavage of the chiral auxiliary using LiOH in aqueous THF affords atrasentan (**6**) in 78% yield.

This accelerated approval was based on the prespecified interim analysis of the Phase III ALIGN study (NCT04573478), a global, randomized, multicenter, double-blind, placebo-controlled trial evaluating the efficacy and safety of Vanrafia versus the placebo in patients with IgAN at risk of progressive renal function loss [75]. A total of 340 biopsy-proven IgAN patients with baseline total proteinuria ≥ 1 g/day, despite optimized RAS inhibitor therapy, were randomized to receive once-daily oral Vanrafia 0.75 mg or the placebo for approximately 132 weeks. Patients continued stable, maximally tolerated doses of RAS inhibitors as background supportive care (unless intolerant to RAS inhibition). An additional 64 patients receiving a stable SGLT2 inhibitor for at least 12 weeks were also enrolled. The primary efficacy endpoint for the interim analysis was the change in proteinuria, a marker of renal injury, measured as the change in 24-h UPCR from the baseline to Week 36. Secondary and exploration objectives included the assessment of renal function change from the baseline to Week 136, measured by estimated glomerular filtration rate as well as safety and tolerability. In the ongoing Phase III ALIGN study, patients treated with Vanrafia in combination with RAS inhibitors achieved a clinically meaningful and statistically significant reduction in proteinuria of 36.1% compared with the placebo (*p* < 0.0001). Beneficial effects were observed as early as Week 6 and were sustained through Week 36. In the primary study cohort, the effect of Vanrafia on UPCR was consistent across subgroups including age, sex, race, and baseline disease characteristics such as estimated glomerular filtration rate and proteinuria levels. Similar treatment effects were seen in the subgroup of patients receiving concomitant RAS and SGLT2 inhibitors, with a 37.4% reduction in UPCR versus the placebo. Regarding safety, Vanrafia demonstrated a favorable safety profile consistent with previously reported data. Adverse events reported in ≥2% of Vanrafia-treated patients and more frequently than with the placebo included peripheral edema, anemia, and elevated liver transaminases. Clinicians should perform liver enzyme testing prior to initiating Vanrafia and as clinically indicated, as some endothelin receptor antagonists have been associated with elevated aminotransferases, hepatotoxicity, and hepatic failure. In summary, atrasentan is a highly selective ERA. Clinical trial data demonstrates its robust efficacy, excellent safety, and good tolerability, offering a novel therapeutic option and enabling precision dosing for patients.

## 8. Avmapki Fakzynja (Avutometinib & Defactinib)

Low-grade serous ovarian cancer (LGSOC) is a rare form of ovarian cancer that is distinct from high-grade serous ovarian cancer (HGSOC) and requires a different therapeutic approach [76]. Compared with HGSOC, LGSOC is associated with a higher recurrence rate and lower sensitivity to chemotherapy [77]. Approximately 70% of LGSOC cases harbor RAS pathway-related mutations, and 30% of patients with LGSOC have KRAS mutations [78]. Current standard treatments for LGSOC include hormonal therapy and chemotherapy. On 12 May 2025, the U.S. FDA granted accelerated approval to the Avmapki Fakzynja Co-pack (avutometinib & defactinib), a combination therapy developed by Verastem Oncology, for the treatment of adult patients with recurrent low-grade serous ovarian cancer whose tumors harbor a KRAS mutation and who have received prior systemic therapy [79,80]. Prior to the Avmapki Fakzynja combination therapy, no FDA-approved treatments were specifically indicated for KRAS-mutated recurrent LGSOC. Avmapki is a MEK1 inhibitor that targets the MEK protein, which when mutated, causes constitutive activation of the RAS/MAPK pathway. Fakzynja inhibits FAK and proline-rich tyrosine kinase 2 (Pyk2)—two members of the FAK family of non-receptor tyrosine kinases that play critical roles in cancer cell growth, invasion, and survival. This combination is designed to more comprehensively block aberrant signaling that drives RAS/MAPK pathway-dependent tumor proliferation and drug resistance [81].

The synthesis of avutometinib (Figure 9) commences with 5-chloro-2,3-difluoropyridine (**7.1**) [82]. Treatment of **7.1** with acetamide under NaHMDS mediation affords acetamidopyridine **7.2** in 72% yield. Subsequent lithiation–formylation of **7.2**, using LiHMDS and morpholine-4-carbaldehyde, yields aldehyde **7.3** in 66% yield. Knoevenagel condensation of **7.3** with ethyl acetoacetate, followed by catalytic hydrogenation using Pd(OH)_2_/C, furnishes β-ketoester **7.4**. Pechmann cyclization of **7.4** with resorcinol constructs the coumarin core **7.5** in 41% yield. O-Arylation of **7.5** with 2-bromopyrimidine in the presence of K_2_CO_3_ affords ether intermediate **7.6** in 76% yield. Finally, sulfamoylation of **7.6** with methylsulfamoyl chloride delivers avutometinib (**7A**) with 88% yield.

The synthesis of defactinib (Figure 10) proceeds via two convergent fragment streams [83,84]. For the sulfonamide fragment: compound **7.7** undergoes sulfonamidation with methanesulfonamide to afford **7.8** in 95% yield; subsequent N-methylation with methyl iodide furnishes **7.9** in 80% yield; finally, Ni-catalyzed hydrogenation reduces the nitrile group to an aminomethyl functionality, delivering **7.10** in 83% yield. In parallel, tert-butyl 4-aminobenzoate (**7.11**) couples with 2,4-dichloro-5-(trifluoromethyl)pyrimidine (**7.12**) to yield the pyrimidine intermediate **7.13** in 97% yield. Condensation of **7.10** and **7.13** in NMP, mediated by DIPEA, affords the polycyclic ester **7.14** in 76% yield. Acid-mediated deprotection of the tert-butyl ester in **7.14** using 4 M HCl gives benzoic acid **7.15** in 93% yield. Finally, CDI-mediated amidation of **7.15** with methylamine yields defactinib (**7B**) in 94% yield.

This approval was based on the open-label, Phase 2 RAMP201 study (NCT04625270), which evaluated the safety and efficacy of the combination regimen [85]. The study enrolled 57 adult patients with measurable KRAS-mutated recurrent low-grade serous ovarian cancer (LGSOC). Patients received Avmapki 3.2 mg orally twice weekly for the first 3 weeks of each 4-week cycle, and Fakzynja 200 mg orally twice daily for the first 3 weeks of each 4-week cycle, until disease progression or unacceptable toxicity. The primary efficacy outcome measure was overall response rate assessed by a blind independent review committee per RECIST version 1.1. A secondary efficacy outcome was DOR. Tumor response assessments were performed every 8 weeks for the first 72 weeks and every 12 weeks thereafter. Study results demonstrated a confirmed overall response rate of 44% (95% CI: 31, 58), with 3.5% of patients achieving a complete response and 40% achieving a partial response. Duration of response ranged from 3.3 to 31.1 months. Tumor KRAS mutations observed among the 25 responders included A146V, G12D, G12R, G12V, and Q61H. Safety of the Avmzpki and Fakzynja combination was evaluated in 57 patients with KRAS-mutated recurrent LGSOC. Serious potential side effects of the combination included ocular disorders, skin toxicity (rash), hepatotoxicity, rhabdomyolysis, and fetal harm if administered during pregnancy. The most common adverse reactions (including laboratory abnormalities) were elevated blood enzyme levels, nausea, fatigue, abnormal liver function tests, and rash. Medicines approved under the accelerated approval pathway are required to verify their safety and efficacy in larger Phase 3 trials. The Phase 3 RAMP301 trial (NCT06072781) of Avmapki plus Fakzynja will serve as the confirmatory trial, comparing the combination to standard chemotherapy or hormonal therapy in patients with recurrent LGSOC regardless of KRAS mutation status. Verastem stated that in addition to potentially supporting the conversion of accelerated approval to full approval, this Phase 3 study may also support expansion of the combination to the treatment of LGSOC irrespective of KRAS mutation status.

## 9. Tryptyr (Acoltremon)

Dry eye disease (DED) is one of the most prevalent chronic ocular disorders, characterized by persistent symptoms including ocular stinging, burning, photophobia, blurred vision, and eye fatigue [86]. A primary pathogenic mechanism underlying DED involves inflammation and immune-mediated dysregulation leading to tear film instability and dysfunction [87]. DED affects approximately 700 million adults worldwide [88]. Although historically regarded as a condition predominantly associated with aging populations, the increasing prevalence of DED across diverse age groups and both sexes has been significantly driven by modern technological factors—particularly prolonged digital screen use. Currently, artificial tears constitute the mainstay of DED management; however, most patients fail to achieve satisfactory symptom relief. Many ophthalmic care professionals and patients widely perceive existing prescription therapies for DED as suboptimal in efficacy, slow in onset of action, and/or poorly tolerated. On 28 May 2025, the U.S. FDA approved Tryptyr (acoltremon ophthalmic solution) developed by Alcon for the treatment of the signs and symptoms of DED [89]. Tryptyr is the first TRPM8 receptor agonist (neuromodulator), which rapidly increases natural tear production by stimulating corneal sensory nerves. The active ingredient in Tryptyr, acoltremon, is an agonist of the TRPM8 cold thermoreceptor. Stimulation of the TRPM8 cold thermoreceptor has been shown to activate trigeminal nerve signaling, thereby increasing basal tear secretion [89].

Natural products are bioactive compounds derived from animals, plants, insects, marine organisms, and microorganisms, exhibiting diverse chemical structures, serving as a key source for novel drug discovery, and warranting deeper and broader research [90,91,92,93,94,95,96,97,98,99,100,101,102,103,104,105]. Throughout history, natural products have played a pivotal role in combating disease [106,107,108,109,110,111,112,113,114,115]. Notable examples include morphine [116], aspirin [117], camptothecin [118], paclitaxel [119], and artemisinin [120], all either directly derived from or chemically modified from plant sources. Acoltremon is a derivative synthesized from (−)-menthol and features a structural motif reminiscent of natural products. AR-15512, also named WS-12, belongs to the WS-series amid derivatives discovered in the 1970s when daily chemical enterprises screened alternatives to menthol (Figure 11) [38]. Subsequent studies identified it as a highly selective TRPM8 superagonist that barely activates TRPA1 and TRPV3. It elicits twice the maximum current of menthol without rapid desensitization, making it suitable for long-term dry eye treatment. Its potent binding to TRPM8 stems from the six-membered core, large hydrophobic amide side chain at the C3 position, and specific chiral configuration. Cryo-EM results revealed that it targets the allosteric pocket within the transmembrane VSLD domain, stabilizing the open channel conformation via π-interactions, multiple hydrogen bonds, and hydrophobic contacts. Different from the analogue icilin, its activation does not require intracellular calcium, leading to more stable efficacy. WS-12 alone cannot open the ion channel; it triggers conformational shifts to recruit membrane lipid PIP2, which acts as a molecular bridge to gate the pore. Instead of forcibly opening channels, it enhances physiological gating, thus conferring superior safety and tolerability [121,122,123,124,125,126,127,128,129].

The synthesis of acoltremon (Figure 12) begins with chiral, substituted cyclohexanol **8.1** [130,131]. Selective oxidation of the secondary alcohol, mediated by Fe(NO_3_)_3_·9H_2_O/NaCl under an O_2_ atmosphere in 1,2-dichloroethane (DCE) at 70 °C, affords cyclohexanone **8.2** in 98% yield. Subsequent Wittig olefination of **8.2** with (methoxymethyl)triphenylphosphonium chloride, using potassium tert-butoxide (KOtBu) as the base in THF under a nitrogen atmosphere, delivers the methoxymethyl ether **8.3**. Acidic deprotection of **8.3**, effected by treatment with concentrated HCl in chloroform, furnishes chiral cyclohexyl aldehyde **8.4** in 95% yield. Oxidative homologation of aldehyde **8.4**, employing N-hydroxyphthalimide (NHPI) as a catalyst under O_2_ in acetonitrile (ACN) at 70 °C, yields cyclohexanecarboxylic acid **8.5** in 85% yield. Conversion of **8.5** to the corresponding acyl chloride **8.6** is achieved via the reaction with thionyl chloride (SOCl_2_) at 80 °C, proceeding in 80% yield. Finally, amide coupling of acyl chloride **8.6** with 4-methoxyaniline, conducted in a biphasic H_2_O/Et_2_O system buffered with NaHCO_3_, gives acoltremon (**8**). This linear synthetic sequence fully preserves the stereochemical integrity of the cyclohexane chiral centers and proceeds in consistently high yields throughout; notably, the iron-catalyzed aerobic oxidation provides a mild, scalable alternative to conventional strong oxidants.

The approval was based on two Phase 3 clinical trials enrolling more than 930 patients with a history of dry eye disease, randomized 1:1 to Tryptyr or the vehicle [132]. In the COMET-2 (NCT05285644) and COMET-3 (NCT05360966) studies, up to four times as many patients receiving Tryptyr achieved an increase in natural tear production of at least 10 mm on Day 14 compared with the vehicle: 42.6% vs. 8.2% in COMET-2 and 53.2% vs. 14.4% in COMET-3 (*p* < 0.0001). Consistent results were observed at all time points through Day 90. Tryptyr demonstrated a statistically significant increase in natural tear production as early as day 1. The most common adverse event reported in clinical trials was instillation site pain.

## 10. Ibtrozi (Taletrectinib)

Lung cancer is the leading cause of cancer-related death worldwide [133,134]. NSCLC is one of the most common forms of lung cancer, accounting for 85% of diagnosed cases [135,136]. ROS1 fusions occur in 1–2% of all NSCLC patients, most frequently in lung adenocarcinoma, with a slightly higher prevalence of 2–3% in East Asian populations [137,138]. On 11 June 2025, the U.S. FDA approved Ibtrozi (taletrectinib) developed by Nuvation Bio for the treatment of adult patients with locally advanced or metastatic ROS1-positive (ROS1+) NSCLC [139]. This drug is a highly selective, next-generation oral ROS1 tyrosine kinase inhibitor (TKI). In pivotal trials, Ibtrozi demonstrated high response rates and durable efficacy, as well as the ability to penetrate the blood–brain barrier. Coupled with a well-characterized and manageable safety profile, it is poised to become a new standard of care for targeted therapy in this subtype of lung cancer [140]. Taletrectinib is an oral, potent, selective, next-generation ROS1 inhibitor capable of crossing the blood–brain barrier. It is primarily indicated for the treatment of NSCLC, particularly in patients harboring ROS1 gene fusions. Developed to address acquired resistance associated with existing ROS1 inhibitors, taletinib demonstrates robust central nervous system penetration and has shown clinical efficacy in NSCLC patients with brain metastases. Talretinib received its first regulatory approval in China on 20 December 2024 for the treatment of adult patients with locally advanced or metastatic ROS1-positive NSCLC who have previously received ROS1 inhibitor therapy. Subsequently, taletrectinib was approved in China on 3 January 2025 and in the U.S. on 11 June 2025, both for the treatment of adult patients with locally advanced or metastatic ROS1-positive NSCLC. On 19 September 2025, it was approved in Japan for the treatment of adult patients with unresectable advanced and/or recurrent ROS1-positive NSCLC. Additional regulatory submissions for taletrectinib are currently underway in other global markets.

The synthesis of taletrectinib (DS-6051b) (Figure 13) employs a convergent two-fragment strategy [141,142]. The Mitsunobu reaction between 4-bromophenol (**9.1**) and N-Boc-L-alaninol (**9.2**) affords ether intermediate **9.3**, which undergoes palladium-catalyzed borylation with bis(pinacolato)diboron (B_2_Pin_2_) to yield aryl boronate **9.4** in 88% isolated yield. Concurrently, (R)-1-(3-fluorophenyl)ethanamine (**9.5**) reacts with 3-bromo-6-chloroimidazo[1,2-a]pyridine (**9.6**) in DMSO at 120 °C using cesium fluoride (CsF) as the base to furnish heterocyclic bromide **9.7** in 89% isolated yield. Subsequent Suzuki–Miyaura cross-coupling between boronate **9.4** and bromide **9.7**, mediated by a palladium catalyst, delivers the Boc-protected core intermediate **9.8** in 84% isolated yield. Finally, acidic deprotection of the tert-butoxycarbonyl (Boc) group in **9.8** affords taletrectinib (**9**) as the free amine in 91% isolated yield.

Efficacy evaluations were based on two multicenter, single-arm, open-label clinical trials, TRUST-I (NCT04395677) and TRUST-II (NCT04919811), enrolling patients with locally advanced or metastatic ROS1-positive NSCLC [143,144]. The efficacy population included 157 TKI-naive patients (103 from TRUST-I and 54 from TRUST-II) and 113 patients who had received one prior ROS1 TKI (66 from TRUST-I and 47 from TRUST-II). Patients may have received prior chemotherapy for advanced disease. Efficacy evaluations included two multicenter, single-arm, open-label clinical trials: TRUST-I (NCT04395677) and TRUST-II (NCT04919811), enrolling patients with locally advanced or metastatic ROS1-positive NSCLC. The efficacy population comprised 157 patients naive to ROS1 tyrosine kinase inhibitor (TKI) therapy (103 from TRUST-I and 54 from TRUST-II) and 113 patients who had received one prior ROS1 TKI (66 from TRUST-I and 47 from TRUST-II). Patients may have received prior chemotherapy for advanced disease. The median response follow-up duration in TRUST-I was 40 months, with the longest duration of response (DOR) being 46.9 months and ongoing. In TRUST-II, the median response follow-up duration was 19 months, with the longest DOR being 30.4 months and ongoing as of October 2024. Due to the single-arm design of the TRUST trials, median progression-free survival (PFS) was not included in the prescribing information. Consistent results were observed in patients with prior ROS1 TKI treatment (TKI-pretreated) across the pivotal trials. In TRUST-I, IBTROZI achieved a confirmed objective response rate (cORR) of 52% in TKI-pretreated patients, with a median DOR of 13.2 months and median follow-up of 33 months. In TRUST-II, IBTROZI demonstrated a cORR of 62%, with a median DOR of 19.4 months and median follow-up of 19 months as of October 2024. Brain metastases represent one of the most common and severe complications of advanced ROS1-positive NSCLC. IBTROZI is designed to penetrate the central nervous system (CNS) and has demonstrated durable intracranial responses in patients with measurable brain metastases at the baseline. Intracranial responses were achieved in 73% of TKI-naive patients (11/15) and 63% of TKI-pretreated patients (15/24). IBTROZI was generally well-tolerated, with most adverse events being mild, transient, and manageable. Few patients (7%) discontinued treatment due to treatment-emergent adverse events (TEAEs). The most common adverse reactions (≥20%) included diarrhea (64%), nausea (47%), vomiting (43%), dizziness (22%), rash (22%), constipation (21%), and fatigue (20%). Overall, most central nervous system events were mild to moderate (90%) and resolved within days, with a low rate of dose modifications (5%) due to these events. The prescribing information for Ibtrozi also includes the following warnings and precautions: hepatotoxicity, interstitial lung disease/pneumonitis, QTc interval prolongation, hyperuricemia, myalgia with elevated creatine phosphokinase, fractures, and embryo-fetal toxicity.

## 11. Zegfrovy (Sunvozertinib)

According to the latest national cancer statistics released by the National Cancer Center of China, lung cancer accounts for approximately 82% of all cancer cases in the country—a striking figure [145,146]. Lung cancer ranks first among male cancer patients (accounting for ~50% of cases) and second among female patients (representing over 23% of cases). Given these sobering statistics, the timely approval and launch of sunvozertinib are urgently needed. On 2 July 2025, the U.S. FDA granted accelerated approval to Zegfrovy (Sunvozertinib) developed by Dizal (Jiangsu) Pharmaceutical for the treatment of adult patients with locally advanced or metastatic NSCLC harboring EGFR exon 20 insertion mutations [147]. It becomes the first Chinese innovative drug approved in the U.S. FDA for this indication. The EGFR mutation must be confirmed by an FDA-approved test, and patients must have experienced disease progression following platinum-containing chemotherapy. Historically, there has been a persistent lack of approved oral tyrosine kinase inhibitors (TKIs) for metastatic NSCLC patients with EGFR exon 20 insertion mutations. The unmet clinical need became even more urgent after mobocertinib (Exkivity) voluntarily withdrew this indication in 2023. Therefore, the approval of sunvozertinib (DZD9008) precisely fills this treatment gap, providing a much-needed oral, convenient therapeutic option for patients whose disease has progressed during or after platinum-based chemotherapy. Shovatinib is the first Chinese-developed innovative drug for lung cancer to receive Breakthrough Therapy Designation in both China and the U.S., and the only EGFR Exon20ins-targeted therapy for NSCLC in China.

The synthesis of sunvozertinib (Figure 14) follows a linear synthetic sequence [148,149,150,151]. Treatment of methyl 2-amino-4-chloro-5-fluorobenzoate (**10.1**) with methylmagnesium bromide (CH_3_MgBr) in THF affords tertiary alcohol **10.2** in 96% yield via a Grignard reaction. Subsequent nucleophilic aromatic substitution (S_N_Ar) of **10.2** with 2,4-dichloropyrimidine, mediated by N,N-diisopropylethylamine (DIEA) in isopropanol (i-PrOH), yields pyrimidine intermediate **10.3** in 67% yield. A second S_N_Ar reaction between **10.3** and 4-amino-5-fluoro-2-methoxynitrobenzene constructs the tricyclic scaffold **10.4** in 86% yield. N-Alkylation of **10.4** with N-methylpyrrolidine under K_2_CO_3_ catalysis furnishes compound **10.5** in 93% yield. Catalytic hydrogenation of the nitro group in **10.5** using Pt/C affords the corresponding primary amine **10.6** in 94% yield. Finally, amide coupling of amine **10.6** with 3-chloropropanoyl chloride in DCM, followed by intramolecular cyclization in CH_3_CN, delivers sunvozertinib (**10**) in 82% overall yield.

The FDA’s accelerated approval was based on efficacy data from the WU-KONG1B trial (NCT03974022), a multicenter, open-label, randomized trial [152,153]. Eligible patients had locally advanced or metastatic NSCLC with EGFR exon 20 insertion mutations and disease progression after platinum-based chemotherapy. The primary efficacy analysis population included 85 patients treated with sunvozertinib 200 mg orally once daily with food, until disease progression or unacceptable toxicity. The primary efficacy endpoint was confirmed ORR assessed by blind independent review committee (BIRC) as per RECIST v1.1. The key secondary endpoint was DOR assessed by BIRC. Results showed an ORR of 46% (95% CI: 35, 57) and a median DOR of 11.1 months (95% CI: 8.2, not evaluable). Among the patients treated with sunvozertinib, common adverse reactions were predominantly gastrointestinal and skin-related events, most of which were Grades 1–2 in severity. Common Grade ≥3 adverse events (represented by the 300 mg dose) included diarrhea (17.1%), elevated creatine phosphokinase (10.8%), anemia, rash, elevated lipase, leukopenia, hypokalemia, decreased appetite, and asthenia (each ranging from 2.7% to 3.6%). In summary, sunvozertinib, as a targeted therapeutic agent, has demonstrated favorable indications and substantial clinical efficacy in the treatment of locally advanced or metastatic NSCLC. Although certain adverse events have been observed, appropriate management strategies can effectively mitigate these effects, thereby significantly improving both the patients’ quality of life and overall survival. Ongoing and future research endeavors are expected to further advance the field of targeted therapy, offering renewed hope for individuals with NSCLC. This drug has become the third Chinese-developed targeted therapy approved for marketing in the United States—following toripalimab and tislelizumab—marking a pivotal transition in China’s lung cancer drug development paradigm: from “catching up” to “running alongside” global leaders in innovation.

## 12. Ekterly (Sebetralstat)

HAE is a rare autosomal dominant disorder characterized by deficiency or dysfunction of the C1 esterase inhibitor, leading to dysregulated bradykinin generation and consequent episodic, localized swelling of the skin and submucosal tissues [154,155]. These attacks are unpredictable and can occur at any anatomical site; however, laryngeal involvement poses a life-threatening risk due to potential upper airway obstruction. Historically, acute HAE treatment has relied on the subcutaneous or intravenous administration of therapeutic agents [156,157]. Yet this parenteral route presents substantial practical limitations: drug preparation and injection procedures are time-consuming and technically demanding, resulting in an average delay of over three hours from symptom onset to treatment initiation. Such delays adversely affect clinical outcomes: later intervention is consistently associated with more severe symptoms and prolonged attack duration. Consequently, there remains an urgent unmet medical need for an oral acute-phase therapy that combines rapid onset of action with ease of use. To address this challenge, researchers have focused on plasma kallikrein (a pivotal enzyme in the HAE pathogenic cascade) as a therapeutic target. By inhibiting plasma kallikrein activity upstream, bradykinin production can be effectively suppressed, thereby preventing the development of angioedema at its origin. Guided by this mechanistic rationale, an oral plasma kallikrein inhibitor sebetralstat was developed on 7 July 2025 by the U.S. FDA for the treatment of HAE in adult and pediatric patients aged 12 years and older [158]. By inhibiting the cleavage of high-molecular-weight kininogen, the drug reduces bradykinin production, thereby alleviating the symptoms of HAE [159].

The optimization (Figure 15) and synthesis of sebetralstat (Figure 16) commence from 4-(chloromethyl)benzyl alcohol (**11.1**) [160]. Nucleophilic substitution of **11.1** with 2-hydroxypyridine, mediated by K_2_CO_3_ in acetone at 50 °C, affords pyridone alcohol **11.2** in 78% yield. Subsequent mesylation of **11.2** with methanesulfonyl chloride (MsCl) and Et_3_N in DCM furnishes chloromethyl pyridone **11.3** in 93% yield. N-Alkylation of **11.3** with methyl 3-(methoxymethyl)-1H-pyrazole-4-carboxylate, promoted by K_2_CO_3_ in DMF at 60 °C, yields pyrazole ester **11.4** in 54% yield. Hydrolysis of the ester moiety in **11.4** under basic conditions, using aqueous NaOH, gives carboxylic acid **11.5** in 34% yield. Finally, amide coupling of **11.5** with (3-fluoro-4-methoxypyridin-2-yl)methanamine, mediated by HATU in DCM and triethylamine, delivers sebetralstat (KVD900, **11**) in 64% yield.

This approval was based on data from the randomized, three-arm, crossover Phase 3 KONFIDENT trial (NCT05259917), which evaluated the efficacy and safety of sebetralstat in pediatric and adult patients aged 12 years and older with type 1 or type 2 HAE [161]. Study participants were randomized to receive sebetralstat 300 mg, sebetralstat 600 mg, or the placebo. The primary endpoint was time to onset symptom relief, defined as at least “minimally improved” at 2 consecutive time points within 12 h after the first dose, assessed using the Patient Global Impression of Change scale. Study results demonstrated that HAE attack symptom relief was significantly faster in the sebetralstat 600 mg group compared with the placebo. Among the patients treated with sebetralstat 600 mg, the median time to onset of symptom relief was 2.0 h (95% CI: 1.5, 2.8). Results also showed a statistically significant faster time to first reduction in severity (assessed by Patient Global Impression of Severity [PGI-S]) within 12 h after the first dose in the sebetralstat 600 mg group versus the placebo. The median time to severity reduction for sebetralstat 600 mg was 9.1 h (95% CI: 3.8, not reached). In addition, time to attack resolution (PGI-S of “none” within 24 h after the first dose) was significantly faster in the sebetralstat 600 mg group than in the placebo group. Although evaluated, the 300 mg dose of sebetralstat is not recommended for treating HAE attacks. For safety, the most frequently reported adverse reaction with sebetralstat was headache. These findings were further supported by the real-world, open-label KONFIDENT-S extension trial, which as of September 2024 demonstrated that sebetralstat allowed patients to treat attacks within 10 min of onset. Updated data from KONFIDENT-S showed a median time to onset of symptom relief of 1.3 h in attacks involving the larynx and abdomen, as well as in patients receiving long-term prophylaxis. The safety profile of sebetralstat 600 mg in more than 1700 attacks in the open-label extension was consistent with that observed in the KONFIDENT trial. As per FDA labeling, the recommended dose is 600 mg taken as a single dose at the first sign of an acute HAE attack. An additional 600 mg dose may be administered at least 3 h after the first dose if needed (maximum 1200 mg in 24 h). Dosage adjustment should be considered in patients taking CYP3A4 inhibitors or inducers and in patients with hepatic impairment.

## 13. Anzupgo (Delgocitinib)

Chronic hand eczema (CHE) is a heterogeneous, recurrent inflammatory disease affecting approximately one in ten adults worldwide, with the main symptoms including pruritus and pain [162]. It is pathophysiologically characterized by impaired skin barrier function, cutaneous inflammation, and altered skin microbiota, which can severely interfere with daily life [163]. The disease imposes substantial psychological, social, and occupational burdens on patients. Anzupgo is a topical pan-JAK inhibitor that suppresses activation of the JAK-STAT signaling pathway implicated in the pathogenesis of chronic inflammatory skin diseases [164]. Anzupgo was previously approved by the European regulatory authorities for the treatment of CHE last year. On 23 July 2025, the U.S. FDA approved Anzupgo (delgocitinib) cream (20 mg/g) developed by LEO Pharma for the topical treatment of moderate to severe CHE in adult patients who have had an inadequate response to topical corticosteroids or for whom corticosteroids are not appropriate [165]. It is the first FDA-approved therapy specifically indicated for the treatment of moderate to severe CHE in adult patients. Structure-based optimization of delgocitinib, derived from the 7H-pyrrolo[2,3-d]pyrimidine scaffold, is illustrated in Figure 17 [166,167].

The synthesis of delgocitinib (JTE-052) (Figure 18) proceeds via a linear sequence [167]. Treatment of 2-chloropropanoic acid (**12.1**) with thionyl chloride (SOCl_2_) affords the corresponding acid chloride (**12.2**). Concurrently, γ-bromobutyrolactone (**12.3**) undergoes ring-opening amidation with benzylamine to yield amide (**12.4**). Subsequent condensation of (**12.2**) and (**12.4**) in DMF furnishes intermediate (**12.5**), which undergoes intramolecular cyclization upon treatment with LiHMDS, followed by acidic workup, to deliver the chiral β-lactam (**12.6**) in 95% yield. N-Alkylation of (**12.6**) with potassium phthalimide affords phthalimide (**12.7**); subsequent deprotection using diethylenetriamine (DETA) provides the free amine (**12.8**) in 80% yield. Reduction of (**12.8**) with LiAlH_4_, followed by treatment with succinic acid, yields azetidine disuccinate (**12.9**) in 85.5% overall yield over two steps. A sequential S_N_Ar reaction of (**12.9**) with 4-chloro-7H-pyrrolo[2,3-d]pyrimidine, followed by Pd/C-catalyzed hydrogenation, affords the dechlorinated heterocyclic product in 92% overall yield over two steps. Finally, condensation of this heterocycle with 3,5-dimethyl-1H-pyrazole-1-carbonitrile in acetonitrile (ACN) in the presence of Et_3_N delivers delgocitinib (**12**).

This approval was based on data from two randomized, double-blind, Phase 3 clinical trials, DELTA 1 (NCT04871711) and DELTA 2 (NCT04872101), which enrolled adult patients with moderate to severe CHE who had an inadequate response to topical corticosteroids or for whom topical corticosteroids were not recommended [168]. In both trials, subjects were randomized to apply 20 mg/g Anzupgo or the vehicle cream to affected areas of the hands and wrists twice daily for 16 weeks. The primary endpoint was the proportion of subjects achieving Investigator’s Global Assessment-CHE (IGA-CHE) treatment success at Week 16, defined as a score of 0 (clear) or 1 (almost clear) with an improvement of at least 2 points from the baseline. Both studies met their primary endpoints with statistically significant results. In DELTA 1, IGA-CHE treatment success was achieved in 20% (64/325) of patients receiving Anzupgo compared with 10% (16/162) in the vehicle group. Similarly, in DELTA 2, IGA-CHE success was reported in 29% (91/313) of the Anzupgo group and 7% (11/159) of the vehicle group. The studies also met key secondary endpoints measuring reductions in pain and pruritus. Significantly higher proportions of patients treated with Anzupgo showed improvements of at least 4 points in both the Hand Eczema Symptom Diary pruritus score and pain score compared with those using the vehicle cream. The most common adverse reactions reported during treatment were application site pain, paresthesia, pruritus, erythema, and bacterial skin infections (including finger cellulitis, paronychia, other skin infections, leukopenia, and neutropenia).

## 14. Sephience (Sepiapterin)

Phenylketonuria (PKU) is an inherited disorder caused by defects in the phenylalanine hydroxylase (PAH) gene [169,170]. The PAH enzyme is responsible for converting phenylalanine to tyrosine; when its function is impaired, phenylalanine accumulates in the body to toxic levels [171,172]. Patients with untreated or poorly managed PKU may develop irreversible neurological damage, including intellectual disability, developmental delay, and seizures [173]. On 28 July 2025, the U.S. FDA approved Sephience (sepiapterin) developed by PTC Therapeutics for the treatment of hyperphenylalaninemia in patients with PKU aged 1 month and older, including infants and adults [174]. This approval marks a major advance in the field of PKU therapy, offering the first oral treatment option for a broad age range of patients worldwide. Sepiapterin (PTC923, CNSA-001) is an oral formulation with a dual mechanism of action to enhance the activity of PAH. First, sepiapterin acts as a prodrug that is rapidly absorbed and converted intracellularly to tetrahydrobiopterin, an essential cofactor for PAH. Sepiapterin also exerts an independent chaperone effect, correcting misfolding of the PAH protein and thereby enhancing enzymatic function. Through this dual mechanism, sepiapterin effectively reduces the blood phenylalanine levels and has the potential to treat a broad population of patients with PKU [175].

The synthesis of sepiapterin (Figure 19) begins with the chiral MOM-protected aldehyde **13.1** and 2,4-diamino-6-cyclohexyloxypteridine **13.2** [176,177]. Condensation of **13.1** and **13.2** affords Adduct **13.3**. Subsequent iodine–hydrogen peroxide-mediated asymmetric hydroxylation converts **13.3** to **13.4** in an overall yield of 61% across these two steps. Oxidation of secondary alcohol in **13.4** using TPAP/NMO furnishes the ketone intermediate **13.5**. Cleavage of the MOM protecting group is achieved by treatment of **13.5** with HCl in methanol at 50 °C, yielding the free hydroxy ketone **13.6** in 94% yield. Finally, Pd/C-catalyzed hydrogenation of **13.6** under mild heating delivers sepiapterin (**13**) in 59% yield.

This approval was based on the two-part Phase 3 APHENITY trial (NCT05099640), which evaluated the efficacy of sapropterin in pediatric and adult patients aged 1 to 61 years diagnosed with PKU and hyperphenylalaninemia with blood phenylalanine (Phe) levels ≥600 μM on at least two measurements [178]. Part 1, 157 participants received open-label sapropterin for 14 days at age-based doses (7.5 mg/kg daily for <6 months, 15 mg/kg for 6 to <12 months, 30 mg/kg for 12 months to <2 years, 60 mg/kg for ≥2 years), with 66% achieving a ≥30% reduction in Phe levels, and in Part 2, following a 2-week washout, 98 participants aged ≥2 years who had a ≥30% Phe reduction in Part 1 were randomized 1:1 to 6 weeks of sapropterin (dose-escalated from 20 to 60 mg/kg/day) or the placebo, with the primary endpoint of mean change in blood Phe from the baseline to Weeks 5–6 showing that sapropterin reduced the Phe levels by 64.2% (95% CI: 74.1–54.4) relative to the placebo, representing a mean change of −415.8 μmol/L versus −19.9 μmol/L (treatment difference: −395.9 μM; 95% CI: −463.1 to −328.7; *p* < 0.0001), and the most common adverse reactions reported were diarrhea, headache, abdominal pain, hypophenylalaninemia, discolored feces, and oropharyngeal pain.

## 15. Vizz (Aceclidine)

Presbyopia is an inevitable age-related decline in near vision that affects the daily lives of virtually all individuals aged 45 years and older [179,180]. With advancing age, the eye’s crystalline lens progressively stiffens, resulting in a diminished capacity to alter its shape. This loss of lens elasticity impairs the lens’ ability to focus light from nearby objects onto the retina. On 31 July 2025, the U.S. FDA approved Vizz Eye Drops (1.44% Aceclidine ophthalmic solution), developed by LENZ Therapeutics, as the first Aceclidine-based eye drop for the non-invasive treatment of presbyopia in adults [181]. This approval represents an important advance in the management of presbyopia, as it offers patients a non-surgical option to improve near vision. However, while Vizz demonstrated rapid improvement in near vision in clinical trials, direct randomized controlled studies comparing it with conventional agents such as pilocarpine or other pharmacological approaches remain lacking. Thus, its relative advantages in clinical practice still require further validation. The mechanism of action of Vizz relies primarily on its properties as a selective miotic agent. It effectively constricts the iris sphincter muscle to produce a pupil diameter of less than 2 mm. This pinhole effect enhances the depth of focus and thereby improves near vision. Compared with traditional agents such as pilocarpine, Vizz exhibits greater pupil selectivity, meaning that it causes minimal stimulation of the ciliary muscle and may therefore offer advantages in reducing certain side effects.

The synthesis of aceclidine (Figure 20) starts from 1-azabicyclo[2.2.2]octan-3-ol **14.1** and acetic anhydride **14.2** [182]. O-Acetylation of the hydroxyl group proceeds via acyl substitution with triethylamine as the base at 160 °C, affording aceclidine (**14**) in 81% yield.

This approval was based on two randomized, double-blind, Phase 3 trials, CLARITY-1 (NCT05656027) and CLARITY-2 (NCT05753189), which evaluated the efficacy of Aceclidine in 466 adults aged 45 to 75 years with spherical power ranging from −4.00 to +1.00 D, astigmatism up to 2.00 D, and spherical equivalent myopia not exceeding −4.00 D, including patients post-refractive surgery and/or with pseudophakic eyes [4,183]. In CLARITY-1, subjects were randomized to receive once-daily Aceclidine or brimonidine for 42 days, while in CLARITY-2, subjects were randomized to Aceclidine or the vehicle control. The primary endpoint was the proportion of subjects achieving an improvement of 3 or more lines in corrected near vision at 40 cm and a loss of no more than 1 line (≥5 letters) in corrected distance vision at 4 m at 3 h post-dose on Day 1. Results showed that 65% of patients treated with Aceclidine met the primary endpoint in CLARITY-1, compared with only 12% of those receiving brimonidine (*p* < 0.01), and similar findings were observed in CLARITY-2, with 71% of the Aceclidine group achieving the primary endpoint versus 8% in the vehicle group (*p* < 0.01). Notably, both trials demonstrated that Aceclidine improved near vision within 30 min and sustained effects for up to 10 h after administration. The most common adverse reactions included ocular irritation (20%), blurred vision (16%), and headache (13%); these reactions were generally mild and transient, resolving spontaneously in most patients, and certain rare but serious adverse events, such as allergic reactions or retinal detachment, have also been documented in postmarketing surveillance databases.

## 16. Modeyso (Dordaviprone)

Diffuse midline glioma (DMG) is a rare and highly aggressive form of pediatric high-grade glioma, predominantly affecting children and young adults [184,185]. H3 K27M-mutant diffuse glioma is a rare malignant brain tumor and is associated with poor prognosis [186]. Because these tumors are predominantly located in midline structures of the brain, surgical resection is often not feasible, and radiation therapy remains the current standard of care. On 6 August 2025, the U.S. FDA granted accelerated approval to Modeyso (dordaviprone) developed by Jazz Pharmaceuticals, for the treatment of recurrent or progressive H3 K27M-mutant DMG in adults and pediatric patients aged 1 year and older [187,188]. This approval marks the first systemic therapy approved for this highly aggressive and refractory brain tumor. Dordaviprone is a mitochondrial caseinolytic protease P activator that acts via multiple mechanisms, including activation of the integrated stress response, induction of tumor cell apoptosis, and restoration of histone H3 K27 trimethylation in H3 K27M-mutant tumors [189]. It also exhibits dopamine D2 receptor inhibitory activity. These effects underpin its promising preclinical antitumor activity and provide a targeted therapeutic option for patients with this genetically defined malignancy.

The synthesis of dordaviprone (ONC201) (Figure 21) commences with the formation of the corresponding acid chloride from 4-chloronicotinic acid (**15.1**) using SOCl_2_ at 90 °C. Subsequent amide coupling with 2-(methylthio)-2-imidazoline hydroiodide, mediated by Et_3_N in DCM, affords amide **15.2** in 96% yield [190]. Cyclization of **15.2** via reflux with o-methylbenzylamine and potassium phosphate (K_3_PO_4_) in N,N-dimethylacetamide (DMAC) furnishes the fused tricyclic intermediate **15.3** in 79% yield. Reduction of the pyridine ring to the corresponding piperazine moiety is achieved via high-pressure hydrogenation of **15.3** using platinum dioxide (PtO_2_) under 45 psi H_2_ in a MeOH/TFA mixture, yielding piperazine derivative **15.4** in 80% yield. Finally, reductive amination of **15.4** with benzaldehyde, employing sodium triacetoxyborohydride as the reducing agent in a mixture of AcOH and DCM, delivers dordaviprone (**15**) in 87% yield.

This approval was based on a pooled analysis of data from five clinical studies, including one Phase I trial (NCT03416530), two Phase II trials (NCT02525692, NCT03295396), one single-patient expanded access program (NCT05392374), and one expanded access study (NCT03134131) [191,192]. The analysis included a total of 50 subjects: adult patients (aged 18 years and older) received 625 mg oral dordaviprone, while pediatric patients received weight-based dosing. Dosing was administered either once weekly or once every three weeks, with treatment cycles of 3 to 4 weeks depending on study design. Efficacy was evaluated using the Response Assessment in Neuro-Oncology 2.0 criteria, with ORR as the primary endpoint. By blind independent central review (BICR), the ORR was 22% (95% CI: 12, 36). The median DOR was 10.3 months (95% CI: 7.3, 15.2), with 73% of patients maintaining a response for at least 6 months and 27% for at least 12 months. The safety profile of dordaviprone was assessed in 376 adult and pediatric patients with glioma across four open-label clinical studies. Serious adverse reactions occurred in 33% of patients; those reported in more than 2% of patients included hydrocephalus, vomiting, headache, seizures, and muscle weakness. The most common adverse reactions in patients receiving dordaviprone were fatigue, headache, vomiting, nausea, and musculoskeletal pain. The approval of dordaviprone marks a significant milestone in oncology, representing the first targeted systemic therapy approved for adult and pediatric patients aged 1 year and older with DMG who experience disease recurrence following prior treatment. We anticipate that ongoing clinical trials will yield robust efficacy and safety data, enabling timely regulatory approval and broad clinical implementation to benefit a greater number of patients.

## 17. Hernexeos (Zongertinib)

Lung cancer is the leading cause of cancer-related mortality worldwide. By 2040, the global incidence of lung cancer is projected to exceed 3 million cases [193,194]. In the field of lung cancer, HER2 mutations are predominantly characterized by “kinase domain insertions”, a structural alteration that impairs the effective binding of conventional HER2-targeted therapeutics to their intended target [195,196]. Consequently, the development of TKIs in oncology research capable of precisely recognizing and engaging these mutant structures has long represented a major challenge, often described as a “hard nut to crack”. Zongertinib is a novel oral selective HER2 tyrosine kinase inhibitor developed by Boehringer Ingelheim. It binds covalently to the HER2 tyrosine kinase domain, including exon 20 insertion mutations, without affecting wild-type epidermal growth factor receptors [197]. On 8 August 2025, the drug received accelerated approval from the U.S. FDA for the treatment of adult patients with unresectable or metastatic non-squamous NSCLC whose tumors have activating mutations in the HER2 (ERBB2) tyrosine kinase domain (TKD) confirmed by an FDA-approved test and who have received prior systemic therapy [198]. It is the first oral HER2-targeted therapy for lung cancer and the world’s first oral small-molecule inhibitor for this mutation subtype.

Zongertinib’s clinical success stems from its rationally designed molecular architecture (Figure 22) [197,199]. Researchers have demonstrated that the compound exhibits high-affinity binding to the ATP-binding pocket of mutant HER2, thereby effectively inhibiting downstream oncogenic signaling at its source. Unlike earlier-generation inhibitors, zongertinib displays markedly reduced off-target activity against EGFR, resulting in a more favorable safety profile with fewer adverse effects. Furthermore, zongertinib demonstrates partial blood–brain barrier penetration, suggesting potential therapeutic efficacy in patients with central nervous system metastases—a clinically critical advantage in HER2-mutant non-small cell lung cancer, where approximately 30% of patients develop brain metastases. To address the poor selectivity of conventional HER2 inhibitors against wild-type EGFR, researchers screened over 12,000 compounds and identified a compound bearing a pyrimido[5,4-d]pyrimidine scaffold. An acrylamide warhead was installed on the piperazine moiety to covalently target Cys805 of HER2, boosting the antiproliferative potency by more than 80-fold. Subsequent substitution of chlorine with methyl yielded compound 10, which exhibited an IC_50_ below 100 nmol·L^−1^ in Ba/F3 cells driven by HER2 YVMA mutation. To further enhance discrimination against wild-type EGFR, the benzene ring was replaced with diverse bicyclic heteroaromatic groups to modulate binding within the hydrophobic back pocket. BI-1622 with a [1,2,4]triazolo[1,5-a]pyridine moiety retained potent HER2 inhibitory activity while achieving approximately 50-fold selectivity over wild-type EGFR. Structure–activity relationship analysis verified that the N atom at the 4-position of this heterocycle is critical for sustaining binding affinity to HER2. Through multiparameter structural optimization based on two probe compounds, BI-4142 and BI-1622, the final candidate zongertinib (13) was developed. It exerted potent antiproliferative effects on HER2 YVMA-driven Ba/F3 cells with an IC_50_ of merely 16 nmol·L^−1^ and around 100-fold selectivity for mutant HER2 over wild-type EGFR. Its potency was drastically attenuated in cell lines carrying the C805S mutation, confirming that zongertinib achieves high efficacy and low toxicity via covalent engagement with the Cys805 residue.

The synthesis of zongertinib (Figure 23) starts from 6-(methylthio)pyrimido[5,4-d]pyrimidin-4(1H)-one (**16.1**). Treatment of 16.1 with SOCl_2_ affords the chloro-thiol intermediate **16.2** [200,201]. Subsequent S_N_Ar between **16.2** and 3-methyl-4-((1-methyl-1H-benzo[d]imidazol-5-yl)oxy)aniline yields the thioether **16.3** in 93% isolated yield. Oxidation of the methylthio group in **16.3** using hydrogen peroxide (H_2_O_2_) in the presence of sodium tungstate (Na_2_WO_4_) furnishes the corresponding sulfoxide **16.4** in 94% yield. Reaction of **16.4** with N-Boc-piperazine via nucleophilic substitution affords the Boc-protected derivative **16.5** in 87% yield. Deprotection of the Boc group with acetyl chloride generates the free amine **16.6**. Acylation of **16.6** with 3-chloropropanoyl chloride delivers the chloroalkylamide intermediate in 69% yield. Finally, base-mediated elimination, induced by KOH, produces the acrylamide crude product in 91% yield; recrystallization from isopropyl alcohol (IPA) affords purified zongertinib (**16**) in 97% yield.

This approval was based on data from a treatment-naïve cohort in the single-arm, open-label Phase 1b Beamion LUNG-1 trial (NCT04886804) [202,203,204]. Seventy-four patients with unresectable or metastatic non-squamous NSCLC harboring HER2 tyrosine kinase domain mutations received zongertinib 120 mg once daily until disease progression or unacceptable toxicity. The primary endpoint was objective response rate (ORR) assessed by blind BICR as per RECIST version 1.1. Results demonstrated an ORR of 77% (95% CI, 66–85), including 11% complete responses and 65% partial responses. Furthermore, 64% of responders had a duration of response of at least 6 months, and 44% maintained response for at least 12 months. For safety, the most common adverse reactions of Zongertinib included diarrhea, rash, hepatotoxicity, fatigue, nausea, musculoskeletal pain, and upper respiratory tract infection.

For patients, the emergence of zongertinib carries three pivotal implications: (1) As an oral targeted therapy, zongertinib significantly enhances treatment convenience. Historically, many HER2-targeted agents required intravenous infusion; in contrast, zongertinib enables once-daily oral administration with a favorable safety profile and reduced treatment-related toxicity; (2) It offers renewed hope for patients with acquired resistance or those who have exhausted multiple lines of prior therapy—particularly for individuals previously deemed “treatment-refractory” or “therapeutically ineligible”, for whom zongertinib represents a viable new therapeutic option and a meaningful extension of survival opportunity.; and (3) It reinforces “molecular profiling” as a standard of care in lung cancer management. HER2 mutations are not exclusive to breast cancer; their identification in non-small cell lung cancer underscores the critical importance of comprehensive genomic testing for every lung cancer patient—to uncover actionable molecular alterations amenable to precision targeting. In summary, in today’s evolving oncology landscape, “HER2 mutation” is no longer synonymous with therapeutic futility. The advent of zongertinib exemplifies the transformative power of precision medicine: by elucidating the underlying molecular mechanisms of disease, we can identify and deploy highly specific therapeutic “keys” to unlock effective, individualized treatment pathways.

## 18. Brinsupri (Brensocatib)

Bronchiectasis is a serious chronic lung disease characterized by permanent dilation of the airways due to a cycle of infection, inflammation, and lung tissue damage [205,206]. The condition is marked by frequent exacerbations of pulmonary symptoms that require antibiotic treatment and/or hospitalization [207,208]. Most cases of bronchiectasis in adults are non-cystic fibrosis bronchiectasis [209]. On 12 August 2025, the U.S. FDA approved Brinsupri 10 mg and 25 mg tablets, a first-in-class dipeptidyl peptidase 1 (DPP1) inhibitor targeting neutrophilic inflammation developed by global biopharmaceutical company Insmed Incorporated [210]. It is the first globally approved therapy for non-cystic fibrosis bronchiectasis, addressing a long-standing treatment gap [211,212]. The drug is indicated for adults and pediatric patients aged 12 years and older. Brensocatib is an orally administered, reversible DPP1 inhibitor designed to target the underlying cause of bronchiectasis. DPP1 is an enzyme responsible for activating pro-inflammatory neutrophil serine proteases. By inhibiting DPP1, brensocatib reduces NSP activity, a key driver of chronic airway inflammation in patients with non-cystic fibrosis bronchiectasis [213].

Doyle et al. reported potent, selective, metabolically stable first-gen reversible DPP1 inhibitors, culminating in oral candidate Bre-1 (Figure 24) [214]. Rat QWBA revealed aortic binding, halting development pre-clinical due to safety. As compound 4 used an α-amino acid scaffold, non-α-amino acid analogues were explored. Reanalysis identified β-amino acid derivative Bre-2 (pIC_50_ = 6.0) as a starting point. Ring-modified analogues yielded AZD7986 (LLE = 7.6), highly selective over cathepsins S, L, B, K, D, E, Z, H, G. It showed excellent selectivity across >200 in vitro assays and good plasma stability (t_1/2_ > 10 h). Strong in vitro–in vivo clearance correlation (rat/dog) and low human hepatocyte turnover supported once-daily dosing.

The synthesis of brensocatib (INS1007, AZD7986) (Figure 25) starts from 6-chlorobenzo[d]oxazol-2(3H)-one **17.1** [214]. N-methylation of **17.1** with MeI and Cs_2_CO_3_ in DMF gives chlorobenzoxazolone **17.2** in 92% yield. Pd-catalyzed Miyaura borylation of **17.2** affords aryl boronate **17.3**. Suzuki coupling of **17.3** with (S)-tert-butyl (1-cyano-2-(4-iodophenyl)ethyl)carbamate delivers biaryl intermediate **17.4**. Boc deprotection of **17.4** with formic acid provides free amine **17.5**. EDC-mediated amide coupling of **17.5** with (S)-4-Boc-1,4-oxazepane-2-carboxylic acid yields Boc-protected amide **17.6**. Second Boc deprotection of **17.6** in aqueous formic acid furnishes brensocatib (**17**) in 91% yield.

The FDA approval was based on data from the 52-week Phase 3 ASPEN trial (NCT04594369) and the 24-week Phase 2 WILLOW trial (NCT03218917) [215,216]. All adult participants had a confirmed diagnosis of non-cystic fibrosis bronchiectasis and at least two documented pulmonary exacerbations in the 12 months prior to screening. Pediatric participants in the ASPEN trial had at least one pulmonary exacerbation in the preceding 12 months. In the ASPEN trial, 1680 adults and 41 pediatric patients were randomized to receive once-daily oral Brinsupri 10 mg (n = 538), Brinsupri 25 mg (n = 575), or the placebo (n = 563). The primary endpoint was the annualized rate of pulmonary exacerbations over 52 weeks of treatment. Pulmonary exacerbation was defined as worsening of three or more key symptoms within 48 h—increased cough, increased or changed sputum volume/character, increased sputum purulence, worsened dyspnea, reduced exercise tolerance, fatigue/malaise, and/or hemoptysis—resulting in the need for antibiotic therapy. Results showed that compared with the placebo, Brinsupri 10 mg and 25 mg reduced the annual rate of pulmonary exacerbations by 21.1% and 19.4%, respectively. Both doses significantly prolonged time to first exacerbation (10 mg: HR 0.81, 95% CI 0.70–0.95; 25 mg: HR 0.83, 95% CI 0.70–0.97) and increased the likelihood of being exacerbation-free (10 mg: OR 1.41, 95% CI 1.11–1.81; 25 mg: OR 1.40, 95% CI 1.10–1.79). Patients receiving 25 mg also showed significantly less decline in lung function at Week 52, measured by post-bronchodilator FEV1, with a treatment difference of 38 mL (95% CI 11–65) versus the placebo. In the WILLOW trial, 256 adults were randomized to once-daily oral Brinsupri 10 mg (n = 82), 25 mg (n = 87), or the placebo (n = 87). Compared with the placebo, time to first pulmonary exacerbation (the primary endpoint) was significantly longer in patients receiving 10 mg (HR 0.58, 95% CI 0.35–0.95) and 25 mg (HR 0.62, 95% CI 0.38–0.99). The most common adverse reactions of Brinsupri include upper respiratory tract infection, headache, rash, dry skin, hyperkeratosis, and hypertension. The prescribing information also includes warnings and precautions related to gingival and periodontal adverse reactions. Live vaccines should be avoided in patients treated with Brinsupri.

## 19. Wayrilz (Rilzabrutinib)

Immune thrombocytopenia (ITP) is a complex immune dysregulation disorder that causes low platelet counts (<100,000/μL), leading to various bleeding symptoms and an increased risk of thromboembolism [217,218]. In addition to bruising and potentially life-threatening bleeding such as intracranial hemorrhage, patients with ITP may also experience reduced quality of life, including physical fatigue and cognitive impairment [219]. On 29 August 2025, the U.S. FDA approved Wayrilz (rilzabrutinib), a covalent inhibitor developed by Sanofi, for the treatment of adult patients with persistent or chronic ITP who have had an insufficient response to prior therapy [220]. Wayrilz is a novel oral reversible BTK inhibitor. BTK is expressed in B cells, macrophages, and other innate immune cells, and plays a key role in multiple immune-mediated disease processes and inflammatory pathways [221,222]. Through multiple immunomodulatory effects targeting different pathways of the immune system, Wayrilz helps address the underlying causes of ITP. Wayrilz is the first BTK inhibitor approved in the United States for the treatment of immune thrombocytopenia.

Rilzabrutinib, a reversible covalent inhibitor targeting BTK, shares an identical heteroaromatic core scaffold with the approved BTK agent ibrutinib, corresponding to a maximum common substructure value of 100%. Compared with ibrutinib, rilzabrutinib exhibits superior safety performance and enhanced selectivity across the human kinome. X-ray crystallographic analysis confirmed that rilzabrutinib can associate with the BTK protein through two distinct interaction mechanisms: covalent binding and noncovalent binding. Notably, the (S)-enantiomer of rilzabrutinib displays comparable biochemical inhibitory activity against BTK. Nevertheless, only its (R)-stereoisomer features a slow target dissociation rate, which enables sustained occupancy of the kinase active pocket [223,224]. Early clinical development of BTK inhibitors focused primarily on oncology. The covalent irreversible inhibitor ibrutinib is FDA-approved for hematologic malignancies, achieving highly selective and sustained BTK inhibition by targeting a non-catalytic cysteine near the ATP-binding site, even after systemic clearance. Beyond its antiproliferative effects in cancers, preclinical data show that BTK inhibitors rapidly reduce inflammation, neutralize pathogenic autoantibodies, and suppress new autoantibody production—prompting evaluation in autoimmune diseases, including in combination with the irreversible covalent inhibitor Ril-2 (Figure 26). To improve solubility and absorption and thus overcome Ril-2′s pharmacokinetic limitations—a piperazine-based oxadiazole compound (rilzabrutinib, PRN1008) was developed, exhibiting favorable water solubility, intrinsic permeability, and low efflux. Notably, in the HWB CD69 B-cell activation assay, rilzabrutinib demonstrated higher potency (123 nM) than Ril-2 (216 nM) [224].

The synthesis of rilzabrutinib (Figure 27) provides two key fragments independently [224,225]: 4-bromo-2-fluorophenol **18.1** undergoes Chan–Lam etherification to afford diaryl ether **18.2** in 29% yield, and subsequent lithiation-borylation delivers arylboronic acid **18.3** in 92% yield; 3-amino-1H-pyrazolo[3,4-d]pyrimidine **18.4** is iodinated with NIS to yield iodoheterocycle **18.5** in 52% yield and then converted to N-Boc piperidine-substituted iodoheterocycle **18.6** via the Mitsunobu reaction. Pd(dppf)Cl_2_-catalyzed Suzuki–Miyaura coupling of the two fragments furnishes the fused core intermediate **18.7** in 76% yield. Boc deprotection of **18.7** is performed with trifluoroacetic acid to generate free amine **18.8**, followed by amide condensation of **18.8** with cyanoacetic acid in 25% yield to give cyanoacetamide intermediate **18.9**, and final sidechain coupling of **18.9** with oxetane-substituted piperazine in 69% yield to afford rilzabrutinib (**18**).

The FDA approval of Wayrilz was based primarily on positive results from the pivotal Phase 3 LUNA 3 clinical study (NCT04562766), a randomized, multicenter trial evaluating the efficacy and safety of Wayrilz versus the placebo in adult and adolescent patients with persistent or chronic ITP [226,227,228,229]. Patients received oral Wayrilz 400 mg twice daily or the placebo for a 12- to 24-week double-blind treatment period, followed by a 28-week open-label treatment phase and a 4-week safety follow-up or long-term extension. The adolescent portion of the study is ongoing. The primary endpoint was durable platelet response, defined as achieving a platelet count of ≥50,000/μL in more than two-thirds of at least eight weekly assessments during the final 12 weeks of the 24-week blinded period, without rescue therapy. Secondary endpoints included time to platelet response (platelet count ≥ 50 × 10^9^/L, or 30 × 10^9^/L to <50 × 10^9^/L with at least a doubling from baseline without rescue), weeks maintaining a defined platelet threshold, rescue therapy use, physical fatigue scores, and bleeding scores. Analyses showed that at Week 25, 23% of patients in the Wayrilz group achieved a durable platelet response compared with 0% in the placebo group (*p* < 0.0001). The median duration of platelet response was 7 weeks with Wayrilz versus 0.7 weeks with the placebo. Time to first response was also significantly shorter: 36 days with Wayrilz versus not reached with the placebo (*p* < 0.0001). On the ITP Patient Assessment Questionnaire, patients receiving Wayrilz showed an overall improvement of 10.6 points across nine health-related quality-of-life domains compared with 2.3 points in the placebo group; these results were descriptive and not statistically tested. The most common adverse reactions (incidence ≥ 10%) included diarrhea, nausea, headache, abdominal pain, and COVID-19.

## 20. Forzinity (Elamipretide)

Barth syndrome is an extremely rare X-linked genetic disorder that primarily affects males [230,231]. It is caused by mutations in the tafazzin gene, which leads to reduced levels of cardiolipin—a phospholipid critical for mitochondrial function [232,233]. The disease is characterized by mitochondrial abnormalities resulting in exercise intolerance, muscle weakness, debilitating fatigue, heart failure, recurrent infections, and growth retardation [234]. It is associated with a shortened life expectancy, with 85% of early deaths occurring before age 5 [235]. On 19 September 2025, the U.S. FDA granted accelerated approval to Forzinity (elamipretide HCl, SS-31, MTP-131), a first-in-class mitochondrial-targeted therapy developed by the biotechnology company Stealth, for improving muscle strength in adult and pediatric patients weighing at least 30 kg (approximately 66 lbs) with Barth syndrome [236]. This is the first drug approved for the treatment of Barth syndrome and the first approved mitochondrial-targeted therapy. Forzinity is a mitochondrial cardiolipin binder designed to improve mitochondrial structure and function by targeting the inner mitochondrial membrane and reversibly binding to cardiolipin. The drug is also currently being developed for primary mitochondrial myopathies and dry age-related macular degeneration [236]. SS-31 differs from conventional antioxidants: rather than scavenging free radicals, it stabilizes mitochondrial membrane lipids to optimize mitochondrial function at the source and prevent aberrant ROS generation, thereby achieving true cellular anti-aging. The SS-31 peptide embodies the convergence of modern mitochondrial nutrition and precision medicine—not merely an antioxidant, but a fundamental repair and protection strategy for the cell’s “energy core”. Its success could redefine our understanding of aging, cellular health, and chronic disease reversal. In the future, SS-31 may synergize with NAD+ boosters (e.g., NMN, NR), SOD mimetics, and ubiquitin pathway modulators to form a next-generation mitochondrial support system—advancing more effective anti-aging strategies.

The synthesis of elamipretide (Figure 28) begins with the TBTU/NMM-mediated coupling of phenylalaninamide **19.1** and **19.2** to form dipeptide **19.3** in 71.2% yield [237]. Selective hydrogenolytic Cbz deprotection using Pd/C, H_2_, and MeSO_3_H quantitatively produces mesylate salt **19.4**. Condensation of **19.4** with t-Bu-protected dimethyltyrosine affords tripeptide **19.5** (68.9%), followed by identical hydrogenation to yield intermediate **19.6**. Subsequent TBTU-promoted coupling of **19.6** with Cbz-D-Arg sodium salt delivers fully protected tetrapeptide **19.7** (65.1%). Final global deprotection with TFA/IPA cleaves all acid-labile Boc, t-Bu ester, and Cbz protecting groups, affording elamipretide (**19**) at a 50% yield.

The approval was based on data from the TAZPOWER trial (NCT03098797), a Phase 2/3, randomized, double-blind, placebo-controlled, crossover study, and its 192-week, open-label, single-arm extension phase [238]. This randomized trial evaluated the efficacy and safety of once-daily subcutaneous injections of 40 mg Forzinity administered for 12 weeks in 12 subjects aged ≥ 12 years weighing > 30 kg with genetically confirmed Barth syndrome. The primary endpoints of the trial were the 6-min walk distance and the total fatigue score from the Barth syndrome symptom assessment. Forzinity did not demonstrate superiority over the placebo at these primary endpoints. Ten subjects completed the randomized trial and entered the extension phase, which was designed to evaluate the long-term safety and tolerability of Forzinity. Among these 10 subjects, 8 participated through Week 168 of the extension phase. Knee extensor muscle strength, measured using a hand-held dynamometer, was evaluated as one of the secondary endpoints during both the randomized trial and the extension phase. No increase in knee extensor muscle strength was observed during the randomized trial; however, increases were observed during the extension phase. At the baseline follow-up prior to treatment initiation, the median muscle strength (minimum, maximum) was 124 (92, 176) Newtons. In the randomized trial, muscle strength at week 12 increased by a median of 4 (−41, 86) Newtons from the baseline in the Forzinity group compared to a decrease of 5 (−31, 48) Newtons in the placebo group. In the open-label extension phase, improvements in muscle strength from the baseline were observed at Weeks 12, 24, 36, 48, 72, and 168 in patients treated with Forzinity, with median changes of 34 (7, 95), 68 (3, 90), 57 (9, 92), 41 (−2, 144), 35 (−4, 100), and 63 (38, 78) Newtons, respectively. The most common adverse reaction observed in patients treated with Forzinity during the trial was injection site reactions.

## 21. Palsonify (Paltusotine)

Acromegaly is mainly caused by pituitary growth hormone adenomas (accounting for more than 95%) and rarely by tumors in other locations or genetic syndromes [239,240,241]. Chronic excessive secretion of growth hormones leads to symptoms such as enlarged hands and feet, facial changes (including prominent cheekbones and prognathism), and visceral organ hypertrophy [242]. Although surgery is the preferred treatment option, only a portion of patients can achieve complete relief of biochemical indicators through surgery. Long-term drug “stabilization” therapy has become the inevitable choice for those who do not achieve remission after surgery or are not eligible for surgery. For a long time, injectable long-acting formulations have been the only option for traditional treatment, and frequent administration has imposed a heavy burden on patients. On 25 September 2025, the U.S. FDA approved Palsonify (paltusotine), developed by Crinetics Pharmaceuticals, Inc., for first-line treatment of adult patients with acromegaly who have an inadequate response to surgery and/or are ineligible for surgical intervention [243]. As a non-peptide agonist selectively targeting somatostatin receptor subtype 2, Palsonify (paltusotine) has become the first one-daily oral therapeutic agent approved for the management of adult acromegaly [244].

The SS2 agonist paltusotine was derived from a previously documented potent diphenylquinoline lead, which arose from chemical derivatization of a micromolar quinolone amid HTS hit. One notable property of this hit molecule is its dual bioactivity: it acts as a high-affinity gonadotropin-releasing hormone antagonist, yielding an IC_50_ of 3.5 nM (Figure 29) [245].

The synthesis of paltusotine (CRN00808) (Figure 30) starts from 6-bromo-4-hydroxyquinoline **20.1** [245]. Sequential NCS chlorination and PBr_3_ bromination yield dihalogenated quinoline **20.2**, which undergoes nucleophilic substitution with N-Boc-4-aminopiperidine at 140 °C (DIEA) to afford **20.3** in 63% yield. Two successive Suzuki–Miyaura couplings are carried out: reaction of **20.3** with 2-hydroxy-3-cyanophenylboronic acid delivers **20.4** (90% yield), and subsequent coupling of **20.4** with 3,5-difluorophenylboronic acid furnishes core intermediate **20.5** in 52% yield. Boc deprotection of **20.5** with TFA in DCM provides paltusotine (**20**) in 52% yield.

This approval was based on data from the pivotal Phase 3 trials PATHFNDR-1 (NCT05192382) and PATHFNDR-2 (NCT04837040), in which Palsonify demonstrated rapid onset, reliable biochemical control, and sustained efficacy [244,246]. PATHFNDR-1 enrolled 111 adult subjects with biochemically uncontrolled acromegaly randomized to receive Palsonify (n = 54) or the placebo (n = 57) for 24 weeks, with the primary endpoint being the proportion of subjects achieving biochemical control (defined as IGF-1 levels ≤ 1.0 × ULN) compared with the placebo, and a post hoc subgroup analysis of the primary efficacy endpoint showed that 34% (11/32) of Palsonify-treated patients achieved remission versus 3% (1/35) for the placebo in subjects who were treatment-naïve, previously uncontrolled, or had unknown prior control status, while 86% (19/22) of the Palsonify group achieved remission versus 9% (2/22) for the placebo in subjects with prior remission off treatment or documented prior control, and the Palsonify-treated subjects had lower numerical scores on the acromegaly-related symptom severity scale measured by a patient-reported tool assessing headache, joint pain, sweating, fatigue, weakness, swelling, and/or numbness/tingling compared with the placebo; PATHFNDR-2 enrolled 58 patients previously biochemically controlled with long-acting injectable octreotide or lanreotide somatostatin analogues, with the primary endpoint being the proportion maintaining biochemical remission (IGF-1 ≤ 1.0 × ULN) with Palsonify versus the placebo, and Palsonify-treated subjects also had lower symptom severity scores on the patient-reported scale evaluating the same symptoms compared with the placebo group; across both trials, the most common adverse reactions (≥5%) in patients receiving Palsonify were diarrhea, abdominal pain, nausea, decreased appetite, sinus bradycardia, hyperglycemia, palpitations, and gastroenteritis.

Pharmacological treatment of acromegaly is undergoing a pivotal shift—from routine injectable regimens to convenient, at-home oral therapy. The successful clinical implementation of paltusotine validates the efficacy and safety of oral treatment, and its favorable compatibility with commonly prescribed thyroid medications helps alleviate concerns regarding concomitant drug use in clinical practice, thereby offering acromegaly patients a novel therapeutic option that enhances quality of life.

## 22. Inluriyo (Imlunestrant)

Breast cancer ranks as the second most diagnosed cancer globally [247,248]. Among the various molecular subtypes of breast cancer, estrogen receptor-positive (ER+)/human epidermal growth factor receptor 2-negative breast cancer is the most prevalent subtype [249,250]. Up to 40% of patients with advanced or metastatic estrogen receptor-positive (ER+)/HER2-negative breast cancer acquire ESR1 gene mutations during endocrine therapy [251,252]. These mutations confer resistance to standard first-line endocrine therapies, including aromatase inhibitors and selective estrogen receptor modulators (SERMs) [253,254]. In the field of breast cancer treatment, recent advances in research have continuously driven the optimization and innovation of therapeutic strategies. On 26 September 2025, the U.S. FDA approved Inluriyo (imlunestrant), an oral selective estrogen receptor degrader (SERD) developed by Eli Lilly and Company, for adult patients with ER+, HER2–, ESR1mutated advanced or metastatic breast cancer [255]. The approval was supported by results from the EMBER-3 trial, which demonstrated that imlunestrant significantly improved patients’ PFS and reduced the risk of disease progression or death by 38% [256,257]. The approval of Inluriyo brings new therapeutic hope to this patient population.

Imlunestrant, a next-gen oral SERD, was designed to fix pharmacokinetic and efficacy flaws of prior ERα-targeted therapies. Derived from acolbifene (EM-652), a non-degrading SERM with poor oral absorption, the series underwent stepwise optimization [258,259] (Figure 31). Compound **14n** with chiral pyrrolidine boosted ERα degradation to 91% yet weakly suppressed ESR1 mutants. GDC-0927 bearing fluorinated azetidine reached 97% degradation but suffered high hepatic clearance and low bioavailability. As the optimized product, imlunestrant achieves ≥95% ERα degradation, decent oral bioavailability, and potent activity against ESR1 mutants. It antagonizes ERα and triggers its proteasomal breakdown, inhibiting estrogen-dependent transcription and PI3K/AKT signaling to curb tumor growth, effectively overcoming aromatase inhibitor resistance driven by ESR1 mutations. With a rigid quinoline-benzoxepine core and fluorinated azetidine side chain, it outperforms injectable fulvestrant, elacestrant, and preclinical SERDs **14n**/GDC-0927 in oral exposure, BBB penetration, metabolic stability, and anti-resistance potency while retaining ideal oral bioavailability.

The synthesis of imlunestrant (LY3484356) (Figure 32) begins with 4-bromo-3-chloro-7-methoxyquinoline **21.1** and 4-fluorobenzoyl chloride **21.2** [258,259]. Friedel–Crafts acylation gives ketone **21.3** in 68% yield; BBr_3_-mediated demethylation affords phenol **21.4** (92% yield), followed by Mitsunobu etherification to yield **21.5** (61% yield). XPhos Pd G2-catalyzed Suzuki coupling of **21.5** with 2-fluoro-4-(trifluoromethyl)phenylboronic acid delivers biaryl intermediate **21.6** in 78% yield. LiBHEt_3_ carbonyl reduction produces alcohol **21.7** (55% yield), and intramolecular cyclization with NaH in THF furnishes racemic cyclized product **21.8** in 72% yield. Chiral SFC separation finally provides enantiopure imlunestrant (**21**) with *ee* > 99%.

This approval was based on data from the randomized, open-label, active-controlled Phase 3 EMBER-3 trial (NCT04975308), which evaluated the oral estrogen receptor antagonist Inluriyo in adult patients with ER+, HER2- locally advanced or metastatic breast cancer who had been previously treated with an aromatase inhibitor alone or in combination with a CDK4/6 inhibitor [260,261]. Study participants were randomized 1:1:1 to receive Inluriyo 400 mg once daily (n = 138), the investigator’s choice of endocrine therapy (n = 118; fulvestrant 500 mg intramuscularly on Days 1, 15, 29 and monthly thereafter, or exemestane 25 mg once daily), or other investigational combination regimens until disease progression or unacceptable toxicity. The primary endpoint was progression-free survival assessed by the investigator by RECIST v1.1. In the ESR1m population, Inluriyo significantly reduced the risk of disease progression or death by 38% compared with the investigator’s choice of endocrine therapy (fulvestrant or exemestane) (hazard ratio, 0.62 [95% CI, 0.46–0.82]; *p* = 0.0008), with a median progression-free survival of 5.5 months (95% CI, 3.9–7.4) versus 3.8 months (95% CI, 3.7–5.5), respectively. Results also showed an objective response rate of 14.3% in the Inluriyo group (complete response: 0.9%; partial response: 13.4%) versus 7.7% in the endocrine therapy group (complete response: 0%; partial response: 7.7%). As for the analysis, overall survival data were immature, with a mortality rate of 31% reported in the ESR1-mutated population. In the EMBER-3 study, most AEs with Inluriyo were low-grade (Grade 1–2), and the most common adverse reactions (≥10%) included laboratory abnormalities such as decreased hemoglobin, musculoskeletal pain, decreased calcium, neutropenia, increased AST, fatigue, diarrhea, increased ALT, increased triglycerides, nausea, thrombocytopenia, constipation, increased cholesterol, and abdominal pain; 4.6% of patients permanently discontinued treatment due to AEs, 2.4% had dose reductions, and 10% had dose interruptions.

## 23. Rhapsido (Remibrutinib)

Chronic spontaneous urticaria (CSU) is a mast-cell-driven disease thought to arise from immune dysregulation [262,263]. In patients with CSU, the immune system can be activated via either allergic (IgE-mediated) or autoimmune (IgG-mediated) pathways, which triggers BTK activation in mast cells and basophils [264]. Although the mechanism is not fully understood, it is believed that once BTK is activated, it triggers the release of histamine and other pro-inflammatory mediators, leading to the erythema, swelling, and pruritic urticaria characteristic of CSU [265]. H1 antihistamines are the first-line treatment for CSU; however, approximately 60% of patients experience inadequate symptom control with antihistamine monotherapy and persistent CSU symptoms despite treatment [266]. On 30 September 2025, the U.S. FDA approved Rhapsido (remibrutinib), a BTK inhibitor developed by Novartis, for the treatment of adult patients with CSU whose symptoms are inadequately controlled by H1-antihistamines [267,268]. Rhapsido is the only oral targeted BTK inhibitor approved for CSU. Novartis has submitted regulatory applications to global authorities including the European Union, Japan, and China, and has obtained priority review status in China. As a highly selective BTK inhibitor, Rhapsido mechanistically controls the progression of CSU by precisely blocking the BTK signaling pathway and inhibiting the release of histamine and other inflammatory mediators from mast cells and basophils [269,270]. Administered orally twice daily, the drug offers a convenient therapeutic option for patients who are unwilling or unable to receive injectable treatment.

Remibrutinib (brand name Rhapsido), the first oral high-selectivity BTK inhibitor authorized for CSU, is a novel irreversible covalent BTK agent developed via rational structural optimization balancing potency and pharmacokinetic properties. Its development originated from reversible lead CGI-1746 (Figure 33) [223]. Following a design strategy analogous to branebrutinib, remibrutinib was derived from the reversible BTK inhibitor RMB-23a. Guided by the X-ray co-crystal structure of BTK in complex with 23a, researchers deleted the piperazine carboxamide-substituted benzene ring and installed linkers tethered to acrylamide electrophilic warheads. Crystallographic data indicated that RMB-23a stabilizes BTK in an inactive state: its pyrrolopyrimidine core engages the kinase hinge region, and the carboxamide-substituted benzene ring lies adjacent to Cys481, offering a favorable anchor point for electrophilic warheads. Structural derivatization delivered irreversible BTK inhibitor, which achieved subnanomolar BTK inhibitory activity (IC_50_ = 0.9 nM) in biochemical testing. Nevertheless, rapid plasma clearance observed in pharmacokinetic assessments halted further progression. Subsequent research screened diverse hinge-binding backbones and acrylamide-containing linkers to optimize ADMET profiles, ultimately yielding the aminopyrimidine clinical candidate remibrutinib. Remibrutinib binds BTK tightly with a subnanomolar Kd of 0.63 nM and displays remarkable selectivity over other front-region cysteine-containing off-target kinases. Its BTK co-crystal structure confirms covalent conjugation to Cys481: the aminopyrimidine scaffold forms dual hydrogen bonds with hinge residue Met477, and the fluorophenylcyclopropyl group fills the H3 pocket to lock BTK into an inactive conformation, largely accounting for its superior BTK kinome selectivity.

Researchers installed an acrylamide warhead to covalently bind BTK Cys481, transforming the compound into an irreversible inhibitor. Subsequent SAR optimization simplified redundant lipophilic fragments and introduced fluorine on the central phenyl ring to eliminate metabolic vulnerable sites and reinforce binding via multipolar interactions. Scaffold hopping replaced the pyrrolopyrimidine hinge-binding core with 6-aminopyrimidine, and refined piperidine/tetrahydropyridine linkers greatly lowered liver metabolic clearance while improving cell permeability [271,272]. Remibrutinib treats CSU through dual-pathway suppression of disease drivers: BTK blockade in mast cells and basophils halt the secretion of histamine and proinflammatory substances; meanwhile, BTK inhibition in B cells cuts pathogenic autoantibody synthesis [273]. Unlike traditional BTK inhibitors such as ibrutinib, it preferentially binds BTK’s inactive conformation, delivering outstanding kinase selectivity, satisfactory oral bioavailability, and stable plasma retention. Low doses can nearly fully occupy BTK, supporting its clinical application for autoimmune disorders represented by CSU [274].

The synthesis of remibrutinib (LOU064) (Figure 34) commences with 1-bromo-5-fluoro-2-methyl-3-nitrobenzene **22.1**, which undergoes Miyaura borylation to give boronate ester **22.2**, followed by Pd/C-catalyzed nitro hydrogenation to yield anilino boronate **22.3** [274,275]. Separately, methyl 4-bromo-2-fluorobenzoate **22.4** undergoes Suzuki coupling with cyclopropylboronic acid to form ester **22.5**. Condensation of **22.3** and **22.5** promoted by NaHMDS delivers coupled intermediate **22.6** in 76% yield. The second pyrimidine fragment is prepared from 2,4-dichloro-3-methoxypyrimidine **22.7**: hydroxylamine-mediated amination furnishes aminomethoxypyrimidine, and BBr_3_ demethylation affords hydroxypyrimidine **22.8** (59% yield). Mitsunobu alkylation with N-Boc-2-aminoethanol yields N-Boc ether **22.9** (53% yield). Pd-catalyzed Suzuki coupling of **22.6** and **22.9** gives Boc-protected core intermediate **22.10** (74% yield). Sequential TFA-mediated Boc deprotection and T3P-promoted coupling with acrylic acid finally affords remibrutinib (**22**) in 45% yield.

This approval was based on data from two identical 52-week randomized, double-blind, placebo-controlled trials: REMIX-1 (NCT05030311) and REMIX-2 (NCT05032157) [276,277]. Both studies enrolled adult patients with CSU inadequately controlled by H1-antihistamines, defined by persistent pruritus and hives for at least 6 weeks. Eligible patients had a weekly Urticaria Activity Score (UAS7) of at least 16, a weekly Itch Severity Score (ISS7) of at least 6, and a weekly Hives Severity Score (HSS7) of at least 6 during the 7 days prior to randomization. In REMIX-1 and REMIX-2, 39% and 29% of patients, respectively, reported a CSU duration of more than 5 years. Participants were randomized 2:1 to receive Rhapsido 25 mg twice daily (REMIX-1: n = 309; REMIX-2: n = 297) or the placebo (REMIX-1: n = 153; REMIX-2: n = 153) for a 24-week double-blind period, followed by a 28-week open-label phase in which all patients received Rhapsido 25 mg twice daily. The co-primary endpoints were the absolute change from the baseline in ISS7 (sum of daily itch severity scores over 7 days, range 0–3) and HSS7 (sum of daily hives severity scores over 7 days, range 0–3) at Week 12. Results showed significantly greater improvements in pruritus and hives with Rhapsido compared with the placebo. In REMIX-1, the least-squares (LS) mean differences at Week 12 were −2.63 (95% CI: −3.70, −1.56) for ISS7 and −3.61 (95% CI: −4.85, −2.36) for HSS7. In REMIX-2, the LS mean differences were −3.23 (95% CI: −4.29, −2.16) and −4.47 (95% CI: −5.71, −3.23), respectively. Significant improvements were also observed in the key secondary endpoint of change from the baseline in UAS7 (combined ISS7 and HSS7) at Week 12, with LS mean differences versus the placebo of −6.22 (95% CI: −8.45, −4.00) in REMIX-1 and −7.68 (95% CI: −9.91, −5.46) in REMIX-2. In addition, a higher proportion of patients receiving Rhapsido achieved well-controlled disease (UAS7 ≤ 6) in Weeks 2 and 12, as well as complete resolution of itch and hives (UAS7 = 0) at Week 12 compared with those receiving the placebo. Rhapsido has a well-characterized safety profile with no requirement for ongoing laboratory monitoring. The most common adverse events (≥3%) were nasopharyngitis (nasal congestion, sore throat, rhinorrhea), bleeding, headache, nausea, and abdominal pain. Beyond CSU, Novartis is actively advancing the clinical development of Rhapsido in other immune-related disorders including chronic inducible urticaria, hidradenitis suppurativa, and food allergy, demonstrating its potential in the field of immunotherapy.

## 24. Jascayd (Nerandomilast)

Idiopathic pulmonary fibrosis (IPF), one of the most common progressive fibrotic interstitial lung diseases, presents severe survival challenges for patients [278,279]. On 14 October 2025, the U.S. FDA approved Jascayd (nerandomilast) tablets developed by Boehringer Ingelheim for the treatment of IPF [280,281]. It marks the first new therapy for IPF approved in more than a decade, and the first-ever and currently the only PDE4B-preferential inhibitor authorized for this indication. More than a decade has passed without new therapies approved in this field since the launch of nintedanib (Ofev) and pirfenidone (Esbriet). The approval of nerandomilast has not only broken this deadlock, but also provided a novel treatment option for patients. Jascayd is an oral PDE4 inhibitor with antifibrotic and immunomodulatory effects. By inhibiting PDE4, it reduces the expression of profibrotic growth factors and inflammatory cytokines that are overexpressed in idiopathic pulmonary fibrosis [282]. Compared with older therapies, Jascayd offers tolerability advantages and has demonstrated benefits for lung function.

The synthesis of nerandomilast (BI 1015550) (Figure 35) starts with piperidine-catalyzed conjugate addition of methyl thioglycolate **23.1** and methyl acrylate **23.2** to yield thioether diester **23.3** (95–99% yield) [283]. In situ-generated titanium(IV) isopropoxychloride mediates Dieckmann cyclization of **23.3** to give cyclic β-ketoester **23.4**. Condensation of **23.4** with urea **23.5** furnishes amidocarbamate **23.6**, which cyclizes under alkaline conditions to dihydroxypyrimidine **23.7**. POCl_3_ chlorination of **23.7** affords dichlorothiolane pyrimidine **23.8**. Nucleophilic substitution of **23.8** with 3-aminomethyl-3-azetidinol gives amine adduct **23.9** (76% yield). Asymmetric sulfoxidation of **23.9** with t-BuOOH, co-catalyzed by (S)-BINOL and Ti(OⁱPr)_4_, delivers chiral sulfoxide **23.10** (87.4% yield). Final S_N_Ar coupling of **23.10** with 2-chloro-4-(piperidin-4-yl)pyrimidine provides nerandomilast.

The FDA approval was based on two clinical trials: FIBRONEE-IPF (NCT05321069) and Trial 2 (NCT04419506) [284,285]. The primary endpoint of FIBRONEER™-IPF was the absolute change from the baseline in forced vital capacity (FVC, a lung function parameter) at Week 52. Nerandomilast significantly attenuated FVC decline relative to the placebo. The adjusted mean FVC reductions were −106 mL and −122 mL in the 18-mg and 9-mg nerandomilast groups, respectively, versus −170 mL in the placebo group. Moreover, the 18-mg nerandomilast group exhibited an early and sustained therapeutic effect, with a progressively diverging FVC change curve from the baseline over time compared with the placebo. The most common adverse reactions (incidence ≥ 5%) included gastrointestinal disorders such as diarrhea, nausea, vomiting and decreased appetite; psychiatric and nervous system disorders such as depression (including depressed mood, increased depression score and suicidal ideation), headache and dizziness; infections including upper respiratory tract infection; as well as general and metabolic disorders such as fatigue, weight loss, and back pain.

## 25. Lynkuet (Elinzanetant)

Hot flashes are one of the most prevalent symptoms experienced by women during menopause, characterized by sudden episodes of facial flushing, diaphoresis, and palpitations, which significantly impair quality of life [286,287]. Although conventional hormone replacement therapy (HRT) is clinically effective, its use is contraindicated in certain women—particularly those with a history of or elevated risk for breast cancer, venous thromboembolism, or other estrogen-sensitive conditions. Consequently, there remains a persistent unmet need for safe and efficacious non-hormonal alternatives. On 24 October 2025, Bayer announced that its novel agent Lynkuet (elinzanetant) had received U.S. FDA approval for moderate-to-severe vasomotor symptoms (hot flashes) associated with menopause [288]. As the world’s first approved dual neurokinin-1/3 (NK-1/3) receptor antagonist, it initiates a new era of non-hormonal, targeted therapy for menopausal hot flashes. Elinzanetant is a once-daily, non-hormonal, first-in-class oral dual antagonist of the NK1 and NK3 receptors. It acts by modulating the activity of kisspeptin/neurokinin B/dynorphin (KNDy) neurons in the hypothalamus, thereby restoring the thermoregulatory balance disrupted by declining estrogen levels during menopause. Its mechanism of action is distinct from that of traditional hormone replacement therapy. With the approval of elinzanetant, menopausal women will gain access to a safer and more convenient treatment option, establishing non-hormonal therapy as one of the mainstream therapeutic approaches.

The synthesis of elinzanetant (Figure 36) begins with 4-chloro-5-nitropyridin-2(1H)-one **24.1** [289]. Suzuki–Miyaura coupling with 4-fluoro-2-methylphenylboronic acid furnishes pyridone **24.2** in 93% yield. POCl_3_ chlorination delivers dichloropyridine **24.3** in 93% yield, followed by Pd/C hydrogenation to reduce the nitro group and afford amine **24.4** in 98% yield. Amide condensation of **24.4** with 2-[3,5-bis(trifluoromethyl)phenyl]-2-methylpropanoyl chloride produces amide **24.5** in 95% yield, and Cs_2_CO_3_/MeI-promoted N-methylation provides **24.6** in 94% yield. S_N_Ar substitution of **24.6** with (S)-4-(benzyloxymethyl)morpholin-3-amine yields dibenzyl protected intermediate **24.7** in 88% yield. Pd/C hydrogenolysis removes the benzyl group to give alcohol **24.8** in 98% yield, and subsequent alkaline treatment finally affords elinzanetant (**24**) in 97% yield.

This approval was based on data from the OASIS 1 (NCT05042362) and OASIS 2 (NCT05099159) clinical trials, which enrolled 796 menopausal women experiencing at least 50 moderate to severe hot flushes per week, including nighttime episodes [290]. Study participants, with a mean age of 54.5 years, were randomized to receive Lynkuet 120 mg or the placebo once daily at bedtime for 12 weeks. The primary endpoints were the mean changes from the baseline in the frequency and severity of moderate to severe vasomotor symptoms (VMS) in Weeks 4 and 12, including daytime and nighttime hot flushes measured using the Hot Flush Daily Diary (HFDD). From Week 4 onward, Lynkuet significantly reduced the frequency and severity of moderate-to-severe hot flashes; by Week 12, more than 80% of patients achieved a ≥50% reduction, with efficacy sustained through Week 52. Significant improvements in sleep quality, emotional stability, and quality of life were also observed. Administered as a once-daily oral non-hormonal agent, Lynkuet was well-tolerated, with mild headache, fatigue, and somnolence as the most common adverse events. Its unique mechanism modulates hypothalamic KNDy neurons to restore estrogen-disrupted thermoregulation, thereby alleviating hot flashes at the root cause.

## 26. Kygevvi (Doxecitine/Doxribtimine)

Thymidine kinase 2 deficiency is an extremely rare, life-threatening inherited mitochondrial disorder that impairs the body’s ability to produce and repair mitochondrial DNA [291,292]. Symptoms include muscle weakness (myopathy) and respiratory failure. The condition is often fatal, with patients who develop first symptoms at age 12 or younger facing a very high risk of early death, typically within 3 years of symptom onset [293]. To date, no approved therapies exist other than supportive care. On 3 November 2025, global biopharmaceutical company UCB announced that the U.S. FDA had approved Kygevvi (doxecitine/doxribtimine)) powder for oral solution for adult and pediatric patients with thymidine kinase 2 deficiency (TK2d) whose symptoms onset at age 12 or younger [294]. It is currently the only approved therapy for this disorder. Kygevvi is a fixed-dose combination of the pyrimidine nucleosides doxecitine and doxribtimine. It incorporates deoxycytidine and deoxythymidine into skeletal muscle mitochondrial DNA, thereby restoring mitochondrial DNA copy number in TK2d-mutant mice.

The synthesis of doxecitine (Figure 37) adopts a nucleoside synthetic route starting from uracil **25.1** [295]. Silylation with HMDS/TMSCl affords bis(trimethylsilyl)uracil **25.2**. CuI-catalyzed glycosylation of **25.2** yields protected uridine **25.3** in 50% yield. Methanolysis with NaOMe delivers free uridine **25.4** in 93% yield. 5′-DMT protection of **25.4** gives intermediate **25.5**, which undergoes PyAOP-mediated C4-amination to form amino nucleoside **25.6** in 60% yield. TCA deprotection of **25.6** provides doxecitine (**25A**), and the three-step overall yield reaches 72%.

The synthesis of doxribtimine (Figure 38) starts with thymidine **25.7** [296]. Cyclization with diethyl carbonate in DMF containing sodium hydroxide at 120 °C generates fused bicyclic intermediate **25.8**, and the yield is 95.6%. Treatment of **25.8** with HBr in DMF at 40 °C gives brominated riboside **25.9** as a viscous oil that can be directly used for the subsequent step. Nickel-catalyzed hydrogenation in pH 7.4 phosphate buffer finally delivers doxribtimine (**25B**), and the yield is 88.6%.

The approval relied on efficacy and safety evidence from one Phase 2 trial (NCT03845712), two retrospective chart reviews (NCT03701568, NCT05017818), and a compassionate-use expanded-access program, which demonstrated clinical benefit in TK2d patients with symptom onset ≤12 years of age [294,297]. Survival outcomes of treated patients were compared with an external untreated control cohort derived from the published literature and Study 2. Eighty-two genetically confirmed TK2d patients with symptom onset ≤12 years received Kygevvi or pyrimidine nucleoside therapy. Overall survival was assessed against an external untreated control group matched by age at symptom onset (≤2 years or >2 to ≤12 years). Kygevvi reduced the overall risk of death by approximately 86% (95% CI: 61–96%). Three deaths (3.7%) occurred in the treatment arm versus 28 deaths (35.9%) in the controls. Treated patients showed improved survival, with a mean survival of 9.6 years at 10 years post-treatment, compared with 5.7 years in the control group. The most frequent adverse events (≥5% incidence) were diarrhea, abdominal pain (including upper abdominal pain), vomiting, and elevated alanine and aspartate aminotransferase levels.

## 27. Komzifti (Ziftomenib)

AML is an aggressive hematologic malignancy. Mutations in the NPM1 gene occur in approximately 30% of AML cases and represent one of the most prevalent driver genetic alterations in this disease [298]. Early relapse and progressively shortened survival after each recurrence underscore the urgent need for therapies capable of achieving durable remission. Kura Oncology and Kyowa Kirin jointly announced on 13 November 2025, that the U.S. FDA had granted full approval of Komzifti (ziftomenib) for adult patients with relapsed/refractory (R/R) AML harboring susceptible NPM1 mutations and lacking satisfactory alternative therapies [299]. Komzifti is an oral menin inhibitor. Menin is a regulatory protein that normally promotes hematopoietic differentiation; genetic aberrations disrupt its function, leading to uncontrolled proliferation of immature cells. By blocking menin’s aberrant activity, the inhibitor restores normal differentiation and suppresses leukemic growth [300].

Ziftomenib (KO-539) was designed to block the oncogenic menin–MLL protein–protein interaction through four sequential rounds of rational structure-based design guided by protein crystal structures (Figure 39) [301]. Initially, weakly potent lead compounds were obtained from high-throughput screening, and side-chain modifications established the thiazolopyrimidine core scaffold to improve binding potency. Subsequent fluorine substitution and heterocycle replacement reinforced protein binding affinity and metabolic stability. Further heterocyclic transformation eliminated steric hindrance, elevated selectivity against tumor cells, and mitigated off-target toxicity. The final stage involved scaffold remodeling and the installation of lipophilic side chains to simultaneously optimize in vitro inhibitory activity, oral bioavailability, and half-life. Throughout the developmental workflow, multiple draggability criteria encompassing pharmacodynamic efficacy, target selectivity, physicochemical properties, and pharmacokinetic behaviors were holistically balanced, affording a long-acting orally bioavailable small-molecule PPI inhibitor with favorable safety profiles.

The synthesis of ziftomenib (Figure 40) starts from diamino thiophene pyrimidine **26.1** [302,303]. BTC oxidation converts it to dihydroxy intermediate **26.2**, and chlorination with PCl_5_ and POCl_3_ delivers dichlorothienopyrimidine **26.3**. Nucleophilic substitution with tert-butyl 4-aminopiperidine-1-carboxylate provides monoamino-substituted product **26.4**. Methylamine substitution in ethanol generates N-methylated intermediate **26.5**, and TFA-mediated Boc deprotection yields free piperidine **26.6**. Reductive amination with cyanoindole aldehyde furnishes Boc-protected coupled intermediate **26.7**. A second TFA deprotection removes the Boc group to afford secondary amine **26.8**. Final mesylation with MsCl and TEA produces ziftomenib (**26**).

This approval was based on data from the single-arm, open-label Phase 2 KOMET-001 trial (NCT04067336), which evaluated the menin inhibitor ziftomenib in adult patients with relapsed/refractory acute myeloid leukemia (R/R AML) harboring NPM1 mutations, including types A, B, D, and other mutations leading to cytoplasmic localization of the NPM1 protein [300,304]. A total of 112 patients (median age 69 years) received oral ziftomenib 600 mg once daily until disease progression or unacceptable toxicity. Patients who underwent hematopoietic stem cell transplantation during ziftomenib treatment were allowed to resume therapy, and 4 patients (3.6%) received a transplant following ziftomenib. Efficacy was assessed based on complete remission (CR) rate, complete remission with partial hematologic recovery (CRh) rate, duration of CR + CRh, and conversion from transfusion dependence to independence. After a median follow-up of 4.2 months (range 0.1–41.2 months), the CR + CRh rate was 21.4% (95% CI: 14.2–30.2), with a median duration of 5.0 months (95% CI: 1.9–8.1). The CR rate was 17.0% (95% CI: 10.5–25.2), and the CRh rate was 4.5% (95% CI: 1.5–10.1). Among patients achieving CR or CRh, the median time to first response was 2.7 months (range 0.9–15.0 months), and 21 out of 24 responders (88%) achieved a response within 6 months of treatment initiation. At the baseline, 66 patients were dependent on red blood cell and/or platelet transfusions, and 46 were transfusion-independent. Following ziftomenib treatment, 21.2% of transfusion-dependent patients achieved transfusion-independence, and 26% of transfusion-independent patients remained independent for any 56-day period post-baseline. The most common adverse reactions included infection by unspecified organism, hemorrhage, fatigue, bacterial infection, musculoskeletal pain, differentiation syndrome, pruritus, febrile neutropenia, and increased transaminases. Ziftomenib labeling includes a boxed warning regarding differentiation syndrome.

## 28. Hyrnuo (Sevabertinib)

NSCLC is the most common form of lung cancer, activating HER2 mutations occur in 2% to 4% of patients with advanced NSCLC [305,306]. About 80% of NSCLC patients are diagnosed at an advanced stage, making treatment considerably more challenging [307,308]. Currently, treatment options for patients with HER2-mutant NSCLC remain limited, creating a pressing need for more effective therapies. On 19 November 2025, the U.S. FDA granted accelerated approval to Hyrnuo (sevabertinib) developed by Bayer for adult patients with locally advanced or metastatic non-squamous NSCLC harboring activating HER2 TKD mutations and previously treated with systemic therapy [308]. As an oral reversible dual HER2/EGFR inhibitor, Sevabertinib potently suppresses mutant HER2 (including HER2 exon 20 insertions and point mutations) and exhibits high selectivity toward mutant EGFR [309,310]. Its optimized molecular design enhances binding affinity to the HER2 kinase domain while minimizing interaction with wild-type EGFR to reduce off-target toxicity, thereby blocking proliferative signaling and inhibiting tumor growth.

The synthesis of sevabertinib (BAY 2927088) (Figure 41) starts from 2-chloro-6-methoxyaniline **27.1** [311]. Reaction with thiophosgene yields aryl isothiocyanate **27.2**, and the yield exceeds 99%. Nucleophilic addition with tert-butyl 1,3-dioxocyclohexanecarboxylate gives the thioamide adduct, followed by cyclization to form cyclic intermediate **27.3**, and the yield is 71%. TFA-mediated deprotection of the tert-butyl ester in DCM delivers hydroxyl lactam **27.4**, and the yield is 78%. Condensation of **27.4** with (S)-(3-((1,4-dioxan-2-yl)methoxy)pyridin-4-yl)methanamine furnishes sidechain substituted intermediate **27.5**, and the yield is 81%. Final oxidative cyclization in MeOH with TFA and hydrogen peroxide produces sevabertinib (**27**), and the yield is 51%.

This approval was based on efficacy and safety data from the Phase 1/2 SOHO-01 trial (NCT05099172), which evaluated Hyrnuo in patients with unresectable or metastatic non-squamous NSCLC harboring activating HER2 TKD mutations who had received prior systemic therapy [310]. HER2 activating mutations were detected in tumor tissues or plasma by local laboratories before enrollment. The primary efficacy endpoints were confirmed ORR and DOR assessed by blind BICR as per RECIST v1.1. Results supporting approval showed an ORR of 71% (95% CI: 59–82%) with a median DOR of 9.2 months (95% CI: 6.3–15.0), and 54% of responders maintained a response for at least 6 months among patients with locally advanced or metastatic NSCLC (n = 70), with HER2 TKD-activating mutations who had received prior systemic therapy but no prior HER2-targeted treatment. Among the patients (n = 52) who had received prior systemic therapy including HER2-targeted antibody–drug conjugates, the ORR was 38% (95% CI: 25–53%), median DOR was 7.0 months (95% CI: 5.6 months to not evaluable), and 60% of responders had a DOR of at least 6 months. In the safety-evaluable population (n = 44), 95.5% of patients experienced treatment-related adverse events (TRAEs), and 40.9% had Grade 3 TRAEs. The most common TRAEs were diarrhea (86.4%), rash (43.2%), and paronychia (25.0%). Treatment was discontinued due to AEs in 6.8% of patients. The prescribing information includes warnings and precautions for diarrhea, hepatotoxicity, interstitial lung disease/pneumonitis, ocular toxicity, elevated pancreatic enzymes, and embryo-fetal toxicity. Sevabertinib is currently being further evaluated in the global randomized Phase 3 SOHO-02 trial (NCT06452277), which compares sevabertinib with standard systemic therapy in previously untreated patients with HER2-mutated advanced NSCLC.

From a safety perspective, the adverse reactions associated with sevabertinib are primarily mechanism-related and commonly include diarrhea, rash, and elevated hepatic enzymes; most of these events are Grades 1–2 and can be effectively managed through dose modification and supportive care. Notably, the incidence of Grade ≥3 adverse events is relatively low and clinically manageable, and the risk of interstitial lung disease is notably low—this favorable safety profile represents a potential advantage over antibody–drug conjugates. Although overall efficacy remains largely preserved in elderly patients, the incidence of Grade ≥3 diarrhea is increased in this population, warranting heightened clinical vigilance. Pharmacologically, sevabertinib is an orally administered small-molecule TKI, offering advantages including convenient administration, broad tissue distribution, and rapid onset of action. Moreover, its relatively weak inhibition of wild-type EGFR may contribute to a more favorable toxicity profile. However, sevabertinib is predominantly metabolized by CYP3A, conferring a non-negligible risk of drug–drug interactions; thus, concomitant medication use must be carefully evaluated in clinical practice. In summary, sevabertinib demonstrates promising efficacy and a favorable safety profile in patients with HER2-mutant advanced NSCLC, supporting its potential utility both as initial therapy and in later lines of treatment, particularly as a sequential option following ADC failure. Future research should prioritize randomized controlled trials to rigorously define its comparative effectiveness against current standard therapies and to explore its optimal integration into first-line regimens, combination strategies, and real-world clinical practice—ultimately aiming to maximize long-term survival benefits for patients.

## 29. Cardamyst (Etripamil)

Patients with paroxysmal supraventricular tachycardia experience episodes of rapid heartbeat exceeding 100 beats per minute [312,313]. Symptoms may include palpitations, chest discomfort, shortness of breath, dizziness or lightheadedness, and syncope. The condition arises from a short-circuit arrhythmia in the upper chambers of the heart. Symptoms persist until normal heart rhythm is restored. In rare cases, frequent episodes can damage the heart and lead to dilated cardiomyopathy [314]. Previously effective treatments typically required intravenous administration in a medical setting, imposing stress and financial burden on patients. On 12 December, Milestone announced that the U.S. FDA had approved the Cardamyst (etripamil) nasal spray for acute conversion of paroxysmal supraventricular tachycardia to sinus rhythm in adults [315]. It is the first self-administered nasal spray indicated for this arrhythmia. Etripamil is a rapid-onset L-type calcium-channel blocker formulated for intranasal self-administration to terminate acute paroxysmal supraventricular tachycardia episodes outside clinical settings.

The synthesis of etripamil (Figure 42) consists of two fragment syntheses and a final coupling–reduction sequence [316,317]. Starting from 3-cyanophenylpropanoic acid **28.1**, Curtius rearrangement yields Boc-amine **28.2** with a yield of 50%. N-methylation delivers **28.3** with a yield of 90%. Acidic Boc deprotection affords amino acid salt **28.4** with a yield of 68%, and esterification produces amino methyl ester **28.5** with a yield of 93%. For the chiral fragment from 3,4-dimethoxybenzyl cyanide **28.6**, alkylation gives nitrile **28.7** with a yield of 91.3%. Michael addition generates carboxylic acid **28.8** with a yield of 71.4%. Resolution with (R)-1-phenylethylamine provides enantiopure (S)-acid **28.9** with a yield of 25.8%. CDI-mediated coupling of **28.5** and **28.9** forms nitrile amide **28.10** with a yield of 83%. Borane reduction of the cyano group in THF finally affords etripamil (**28**) with a yield of 50%.

Approval was supported by the randomized, double-blind, placebo-controlled Phase 3 RAPID trial (NCT03464019), which evaluated the safety and efficacy of etripamil in patients with symptomatic paroxysmal supraventricular tachycardia (PSVT) [318,319]. A total of 692 participants were randomized 1:1 to self-administer intranasal 70 mg etripamil or the placebo during a PSVT episode without medical supervision, with a second dose permitted if symptoms persisted after 10 min. The primary endpoint was the time to confirm conversion of PSVT to sinus rhythm for at least 30 s within 30 min of the first dose. In patients with confirmed paroxysmal supraventricular tachycardia (PSVT, n = 184), 64% of patients who self-administered Cardamyst (n = 99) achieved restoration of sinus rhythm within 30 min compared with 31% in the placebo group (n = 85), with a hazard ratio of 2.6 (95% CI: 1.7–4.2, *p* < 0.001). The median time to sinus rhythm restoration was 17.2 min in the Cardamyst group versus 53.5 min in the placebo group. The most frequently reported treatment-emergent adverse events included nasal discomfort, nasal congestion, rhinorrhea, throat irritation, and epistaxis. Given the potential risk of dizziness and/or syncope associated with Cardamyst, self-administration is recommended in a seated position.

## 30. Nuzolvence (Zoliflodacin)

Globally, nearly 100 million people are infected annually with gonorrhea, a sexually transmitted infection caused by Neisseria gonorrhoeae [320,321,322]. Over the past decade, the emergence of “super gonorrhea” has raised significant public health concerns. Gonorrhea is the second most common bacterial sexually transmitted infection worldwide [323]. The emergence and global spread of drug-resistant strains have led the World Health Organization (WHO) to identify antibiotic resistance as one of the top 10 most serious global health threats [324]. Neisseria gonorrhoeae has developed resistance to most antibiotics used to treat these infections, including cephalosporins such as the injectable ceftriaxone, which is currently the recommended first-line therapy for uncomplicated urogenital gonorrhea. On 12 December, the U.S. FDA approved Nuzolvence (zoliflodacin) granules by Innoviva Specialty Therapeutics for uncomplicated genitourinary gonorrhea in adolescents aged ≥12 years and adults weighing ≥35 kg [325]. As a novel oral water-soluble antibacterial agent with a new mechanism of action, it represents the first new oral therapy for gonorrhea in nearly a decade. Pharmacologically, Nuzolvence is a first-in-class spiropyrimidinetrione and an oral type II topoisomerase inhibitor that exerts bactericidal activity by disrupting bacterial DNA replication. Its unique structure and mechanism distinguish it from all existing antibiotic classes and confer potent activity against Neisseria gonorrhoeae, including resistant strains. This first-in-class profile makes it a key strategic agent for addressing rising gonococcal resistance [325].

Following high-throughput screening, QPT-1 was identified as the first spiropyrimidinetrione compound to inhibit both bacterial DNA gyrase and topoisomerase IV, without observed cross-resistance to fluoroquinolones (Figure 43) [326,327]. Subsequently, scientists at AstraZeneca introduced a benzisoxazole scaffold. However, owing to concerns regarding toxicity and genotoxicity, additional structural modifications were explored. Building upon this lead compound, an extensive SAR optimization campaign ultimately led to the discovery of zoliflodacin.

The synthesis of zoliflodacin (ETX0914) (Figure 44) starts from 2,3,4-trifluorobenzaldehyde **29.1** [327]. Acetal protection with ethylene glycol gives dioxolane **29.2**, and the yield is 78%. Ortho-lithiation of **29.2** followed by DMF quenching delivers aldehyde **29.3**, and the yield is 93%. Oximation of **29.3** furnishes oxime **29.4**, and the yield is 80%. NCS chlorination converts **29.4** to chlorooxime **29.5**, and the yield is 76%. 1,3-dipolar cycloaddition with chiral amino alcohol yields fused isoxazoline **29.6**, and the yield is 86%. Intramolecular cyclization mediated by CDI affords lactam intermediate **29.7**, and the yield is 53%. Acidic acetal deprotection releases aldehyde **29.8**, and the yield is 99%. Nucleophilic substitution with cis-2,6-dimethylmorpholine gives sidechain substituted aldehyde **29.9**, and the yield is 73%. Knoevenagel condensation of **29.9** with barbituric acid forms alkene **29.10**, which undergoes epimerization via [1,5]-hydrogen shift to generate intermediate **29.11**. Final stereopurification delivers zoliflodacin (**29**), and the overall yield of this final sequence is 71%.

This approval was based on data from an open-label, active-controlled Phase 3 study (ClinicalTrials.gov: NCT03959527) that evaluated the safety and efficacy of Nuzolvence in 930 patients aged 12 years and older, weighing at least 35 kg, with uncomplicated gonorrhea caused by Neisseria gonorrhoeae [328,329]. Participants were randomized 2:1 to receive a single dose of Nuzolvence 3 g or the standard-of-care (SOC) regimen consisting of a single intramuscular dose of ceftriaxone 500 mg plus oral azithromycin 1 g. The primary endpoint was the microbiological response (cure or failure) at the urogenital site at the ToC visit 6 (±2) days post-treatment. The microbiological intent-to-treat urogenital population included 744 patients, 506 treated with Nuzolvence and 238 with SOC, all of whom had Neisseria gonorrhoeae isolated from the urethra or cervix at the baseline and were not infected with strains resistant to both ceftriaxone and azithromycin at the baseline. Results showed that Nuzolvence was non-inferior to SOC in achieving microbiological cure at the urogenital site (90.9% vs. 96.2%, respectively; difference −5.3% [95% CI: −8.7, −1.4]). The most common adverse reactions to Nuzolvence included neutropenia, headache, leukopenia, dizziness, nausea, and diarrhea. The prescribing information also includes warnings and precautions related to embryotoxicity, testicular toxicity, and risk to male fertility.

## 31. Myqorzo (Aficamten)

Obstructive hypertrophic cardiomyopathy (oHCM) is a common inherited heart disorder characterized by abnormal thickening of the heart muscle, which can block blood flow and impair normal cardiac function [330]. Patients often experience symptoms such as dyspnea, fatigue, chest pain, dizziness, or syncope, resulting in severe limitations in daily activities [331]. On 19 December 2025, Cytokinetics announced that the U.S. FDA had approved Myqorzo (aficamten) tablets for adult patients with symptomatic oHCM to improve function and symptoms [332]. Myqorzo is a next-generation selective cardiac myosin inhibitor designed to directly target the underlying pathophysiology of oHCM: excessive myocardial contractility [332]. In oHCM, hyper-activated myosin–actin interactions cause abnormal myocardial hypertrophy and left ventricular outflow tract obstruction. By reducing excessive myosin activity, Myqorzo relieves outflow tract obstruction and improves cardiac filling and ejection efficiency. With appropriate dosing and monitoring, this targeted therapy alleviates symptoms without impairing normal cardiac function [333,334]. Aficamten belongs to a novel class of mechanism-based therapeutics—cardiac myosin inhibitors. Rather than indirectly modulating cardiac function via the nervous system or hemodynamic pathways, aficamten acts directly at the molecular level of myocardial contraction. Cardiac contraction relies on the dynamic cross-bridge cycling between myosin and actin within cardiomyocytes. Aficamten precisely attenuates this pathologically hyperactive cross-bridge formation, thereby exerting a targeted “braking” effect on excessive myocardial contractility at its source. This mechanism aligns perfectly with the core pathophysiological basis of oHCM.

Ficamten was optimized in two rounds of SAR research to strike a balance among potency, target selectivity, and druglikeness (Figure 45) [335,336]. The primary HTS hit compound Af-1 only weakly inhibited cardiac myofibrils (IC_50_ = 2.7 μM), with excessive lipophilicity, poor metabolic stability, and low ligand efficiency. To strengthen target binding, the indoline scaffold was switched to indane, which creates extra hydrogen bonds with Leu267 and Ala463 in the allosteric pocket of cardiac myosin alongside hydrophobic contacts from aromatic and alkyl groups. The resulting compound gained better activity (IC_50_ = 1.0 μM) but still bore unfavorable lipophilicity and clearance profiles. The second optimization stage cut lipophilicity to reduce intrinsic clearance and boost oral exposure: replacing benzoyl amide with polar 1-methylpyrazole-4-carboxamide dropped the cLogP to 2.1 without disrupting key binding interactions. Further stereochemical tuning of the (R)-indane core and ethyl-substituted oxadiazole finally delivered the clinical candidate ficamten. Distinct from conventional ATP-competitive inhibitors, ficamten occupies a unique allosteric pocket on the β-cardiac myosin heavy chain. It slows phosphate release after ATP hydrolysis and locks myosin in an inactive conformation, restricting the shift to its active state. Such allosteric regulation eases myocardial hypercontractility by decreasing functional myosin heads, relieving left ventricular outflow tract obstruction. Launched in 2025 as a cardiac sarcomere modulator, ficamten outperforms the first-in-class mavacamten. Its rigid (R)-indane-oxadiazole-pyrazole carboxamide framework supports predictable binding and gentle dose–response characteristics. It does not interfere with CYP450 enzymes, minimizing drug–drug interactions, and features low intrinsic clearance, straightforward metabolic pathways, moderate half-life, and rapid steady-state concentration attainment.

The synthesis of aficamten (Figure 46) starts from 5-chloroindan-1-one **30.1** [335,336,337]. Pd-catalyzed cyanation delivers cyanoindanone **30.2**. Condensation with tert-butanesulfinamide gives sulfinyl imine **30.3**, and asymmetric reduction yields chiral sulfinamide **30.4**. Acidic deprotection removes the sulfinyl group to afford chiral aminoindane hydrochloride **30.5**. Amide coupling with 1-methylpyrazole-4-carboxylic acid produces amide **30.6**. Treatment with hydroxylamine converts cyano to amidoxime **30.7**, and thermal cyclization with propionic acid furnishes oxadiazole intermediate **30.8**, and the yield is 97%. TFA-mediated deprotection of **30.8** gives free amine **30.9**. Final EDCI/HOAt coupling delivers aficamten (**30**), and the yield is 88%.

FDA approval was primarily supported by positive findings from the pivotal Phase 3 SEQUOIA-HCM trial (NCT05186818), a randomized, double-blind, placebo-controlled study in adults with symptomatic oHCM [338,339,340,341]. The trial demonstrated robust efficacy, safety, and clinically meaningful benefits of Myqorzo across symptomatic, exercise capacity, hemodynamic, and biomarker endpoints. At Week 24, Myqorzo significantly improved exercise capacity versus the placebo, with an increase of 1.8 mL/kg/min in peak oxygen uptake (pVO_2_) assessed by cardiopulmonary exercise testing (CPET), whereas the placebo showed no change (least-squares mean difference: 1.74 mL/kg/min; 95% CI: 1.04–2.44; *p* = 0.000002). Moreover, the treatment effect of Myqorzo was consistent across all prespecified subgroups, including age, gender, baseline patient characteristics, and use of background beta-blocker therapy. Myqorzo was well-tolerated, with no cases of worsening heart failure or treatment discontinuation due to reduced left ventricular ejection fraction (LVEF). Treatment-emergent serious adverse events occurred in 5.6% of patients receiving Myqorzo, compared with 9.3% of those receiving the placebo. Core laboratory echocardiography showed that 5 patients (3.5%) in the Myqorzo group had an LVEF <50% versus only 1 patient (0.7%) in the placebo group. Hypertension (8% vs. 2%) was the only adverse reaction with an incidence exceeding 5% and was more frequent in the Myqorzo arm. Myqorzo-associated blood pressure elevation was consistent with relief of left ventricular outflow tract obstruction and improved cardiac output.

Certainly, it is essential to objectively recognize that drugs such as avelumab are neither standalone “miracle drugs” nor definitive endpoints for all therapeutic approaches. Rather, they will be integrated with conventional pharmacotherapies and surgical interventions into a dynamic, adaptable, and modular treatment matrix. For each individual patient, the selection of specific agents, optimal dosing regimens, and timing of therapeutic intervention must be determined by qualified clinicians based on longitudinal, real-world clinical follow-up and comprehensive assessment. The successful implementation of the first prescriptions of Afu-Ketai across multiple regions in China holds profound significance: it completes the patient’s therapeutic “toolkit” with the most advanced, evidence-based therapeutic option currently available. Under the premise of standardized, multidisciplinary evaluation, patients now have an additional, scientifically grounded choice—one that empowers shared decision-making, and in select cases, offers the potential to meaningfully alter disease trajectory.

## 32. Nereus (Tradipitant)

Motion sickness is caused by a sensory conflict among visual, vestibular, and proprioceptive inputs; this conflict triggers the release of substance P and activation of NK-1 receptors in the central nervous system, leading to nausea and vomiting [342]. On 30 December 2025, the U.S. FDA approved Nereus (tradipitant), developed by Wanda Pharmaceuticals, for the prevention of motion-induced vomiting [343]. As an oral neurokinin-1 (NK-1) receptor antagonist, it represents the first novel pharmacological therapy for motion sickness in over four decades. This approval marks a breakthrough in understanding and managing this debilitating condition. Since the FDA’s approval of the transdermal scopolamine patch in 1979, no novel pharmacotherapies for motion sickness have been approved, marking a gap of over four decades. This recent approval thus represents a significant milestone. It is noteworthy that the regulatory pathway to approval for this drug was not without challenges. In December 2018, the U.S. FDA imposed a partial clinical hold on tradipitant, citing its classification of motion sickness as a chronic condition and requiring the sponsor to conduct an additional six-month chronic toxicity study in dogs. It was not until 4 December 2023 that the FDA revised its classification of motion sickness and consequently rescinded the requirement for the supplemental chronic toxicity study. The agency formally lifted the clinical hold, thereby removing a key regulatory barrier to the drug’s ultimate approval.

A reported route to tradipitant (Figure 47) starts from 3-bromopyridine hydrochloride **31.1** [344]. Sonogashira coupling with trimethylsilylacetylene yields alkynylpyridine **31.2**, and the yield is 96%. Cu-catalyzed azide-alkyne cycloaddition with bis(trifluoromethyl)benzyl azide generates triazole **31.3**, and the yield ranges from 62% to 72%. Iodination of **31.3** with ICI in DCM affords iodotriazole **31.4**, and the yield is 87%. Reductive cross-coupling of **31.4** with (2-chlorophenyl)(2-bromopyridin-4-yl)methanone **31.5** using Zn, ZnCl_2_, and TMSCl in DMF finally delivers tradipitant (**31**) with a 50% yield.

Approval of Nereus was supported by three pivotal clinical trials, including two Phase 3 studies, Motion Syros (NCT04327661) and Motion Serifos (NCT05903924), conducted in real-world maritime settings and one supportive trial in subjects with a documented history of motion sickness [345,346]. In Motion Syros (n = 365), vomiting occurred in 18.3–19.5% of the Nereus group versus 44.3% of the placebo group. In Motion Serifos (n = 316), vomiting rates were 10.4–18.3% with Nereus and 37.7% with the placebo. Across both trials, Nereus reduced the vomiting risk by more than 50% to 70%. Overall, across all key studies, Nereus demonstrated significant efficacy in reducing the incidence of vomiting and a favorable safety profile suitable for short-term use. The most common adverse reactions (incidence ≥ 5%) were somnolence, headache, and fatigue.

## 33. Conclusions and Outlook

Over the past year, the number of first-in-class drugs with novel mechanisms of action have steadily increased, making mechanistic innovation a core driver of drug development. Advances in emerging technologies are continuously enhancing the biopharmaceutical industry’s ability to identify new targets and develop drugs with entirely new mechanisms. Globally, in 2025, new drug R&D continued to be driven by first-in-class therapies and sustained by orphan drugs, while advancing toward higher-level technological integration and global collaboration—fueled by diversified development approaches and increasingly precise clinical needs. In 2025, newly approved drugs by the U.S. FDA continued the prevailing trend of “small-molecule dominance”: small-molecule therapeutics spanned ten major therapeutic areas—including oncology, infectious diseases, and cardiovascular disorders—with six representing first-in-class therapies globally targeting specific molecular pathways. Notable examples include gepotidacin, the first novel agent for uncomplicated urinary tract infections in three decades (a bacterial DNA topoisomerase inhibitor); zoliflodacin, the world’s first oral spiropyrimidinetrione-class antibiotic for urogenital gonorrhea; and suzetrigine, the first non-opioid analgesic—all addressing critical unmet medical needs left unfulfilled by conventional treatments. Notably, in 2025, rare disease therapeutics emerged as the largest indication category among FDA-approved new drugs, with 13 approvals—surpassing oncology (12 approvals)—highlighting a pronounced policy emphasis on underserved patient populations. Specifically, these 13 rare disease drugs targeted 11 distinct conditions. In oncology, the 12 newly approved agents focused predominantly on high-incidence malignancies with eight precision-targeted therapies. For instance, in HER2-mutant NSCLC, two novel agents (sevabertinib and zongertinib) were approved, significantly expanding the therapeutic armamentarium for this molecularly defined patient population. Beyond these categories, cardiovascular diseases, infectious diseases, and pulmonary disorders ranked next in frequency.

At the level of technological breakthroughs and original innovation, small-molecule drugs featuring novel mechanisms accounted for over 70%, effectively dispelling the misconception that “small-molecule therapeutics lack innovation”. Among these, allosteric inhibition and covalent binding have emerged as particularly promising strategies. On the one hand, multiple approved or late-stage clinical candidates employ allosteric inhibition to achieve exceptional target selectivity. For instance, mirdametinib demonstrates approximately 100-fold greater selectivity for mutant targets in type 1 neurofibromatosis compared with conventional orthosteric MEK inhibitors. Similarly, aficamten specifically modulates the contractile-state conformation of cardiac myosin, thereby minimizing off-target effects on non-cardiac tissues in patients with obstructive hypertrophic cardiomyopathy. On the other hand, covalent inhibitors, including the developed EGFR inhibitor sunvozertinib, the HER2 inhibitor zongertinib, and the BTK inhibitor rilzabrutinib, represent four clinically advanced agents that form irreversible (or slowly reversible) covalent bonds with their targets. This mechanism enables sustained target suppression, reduces required dosing frequency and magnitude, and confers activity against certain resistance-associated mutations.

Regarding pharmaceutical regulatory reform, the U.S. FDA introduced several groundbreaking policy initiatives in 2025 concerning the approval of drugs—and in certain cases, related products—targeting core areas such as clinical trial standards for novel therapeutics and accelerated review pathways. These measures aim to strike a balanced approach between expediting the availability of innovative medicines and ensuring robust safety and risk control while also addressing persistent industry challenges, including high drug prices and an inequitable distribution of healthcare resources. First, for novel drug approvals, the FDA shifted from a default requirement of two pivotal clinical trials to a “single pivotal trial” default framework, with confirmatory evidence serving as a critical supplementary component. This represents a fundamental departure from the Agency’s longstanding core review principle—unchanged for over six decades—and stands as the most closely watched regulatory development as of late 2025. The central objective is to reduce the number of required clinical trials while mitigating the inherent uncertainties of single-trial evidence through rigorous post-approval confirmatory data generation. Second, the FDA launched the “Commissioner’s National Priority Voucher” (CNPV), a new, ultra-accelerated review pathway designed to deliver decisions within one to two months. As the Agency’s first-ever “extreme acceleration” mechanism, the CNPV constitutes a significant enhancement over existing expedited programs—including Priority Review and Breakthrough Therapy designation—by focusing specifically on drugs addressing nationally defined public health priorities and enabling an unprecedented compression of review timelines.

The three main drug discovery paradigms to date have been reviewed: serendipitous or empirical drug discovery (ancient times to the 19th century), phenotypic drug discovery (mid-19th century to the 1950s–1960s), and target-based drug discovery (from the 1980s molecular biology revolution to present). Despite steadily rising R&D investment, the persistent “Eroom’s Law”, characterized by high costs and high failure rates in new drug development, reveals a major translational gap in the linear “target identification → animal studies → clinical trials” model. Artificial intelligence (AI)-driven drug discovery has evolved from algorithm optimization to computational infrastructure, entering a new “data-driven + hardware-accelerated” era. AI will play an increasingly constructive role in enhancing the quality of R&D decision-making, accelerating timelines at critical development milestones, and minimizing wasteful investments—ultimately poised to reshape the competitive landscape of novel drug development. In terms of future trends and key focus areas, these interdisciplinary technologies are profoundly reshaping drug development paradigms, enabling innovative therapies to be delivered at lower cost and higher efficiency.

## Figures and Tables

**Figure 1:**
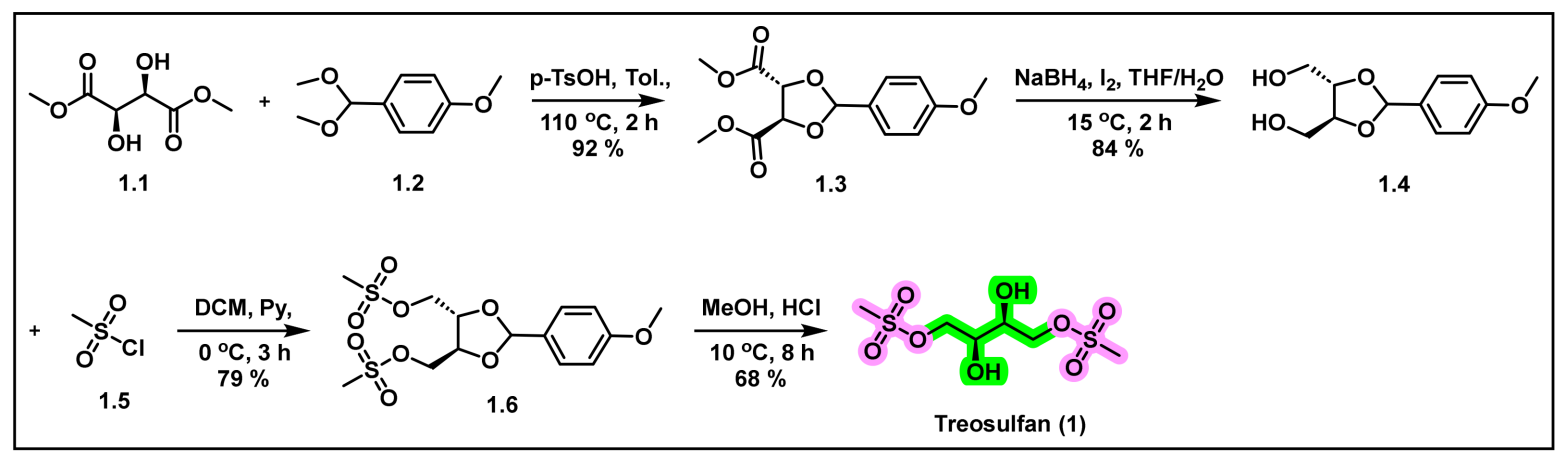
Synthesis of treosulfan.

**Figure 2:**
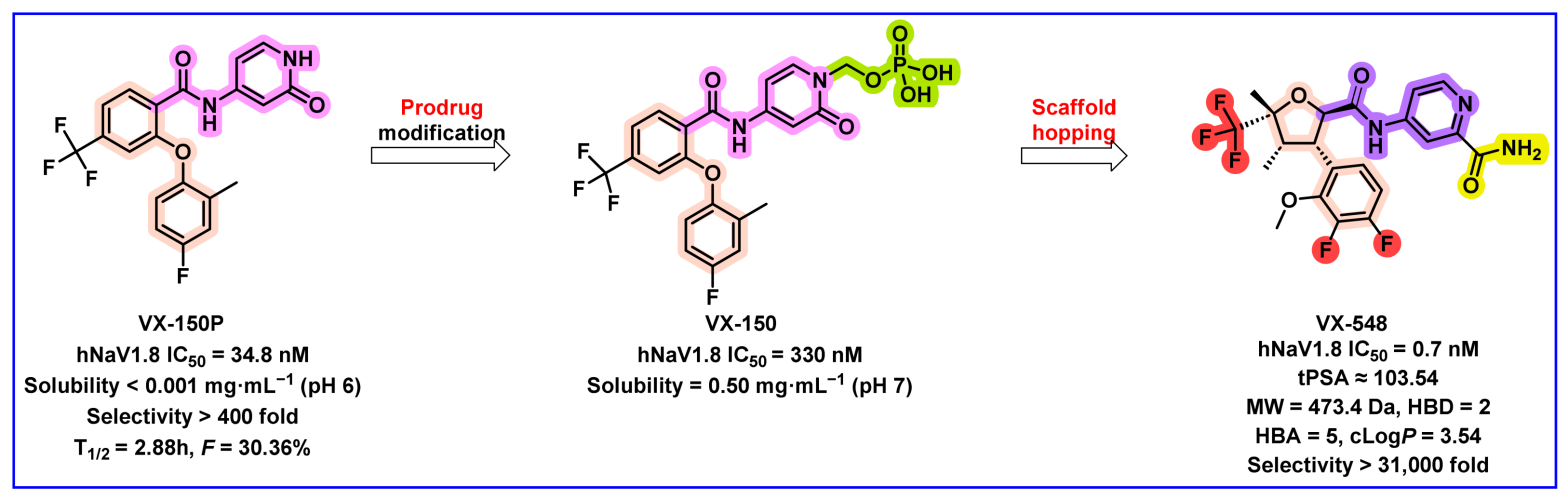
Optimization of suzetrigine.

**Figure 3:**
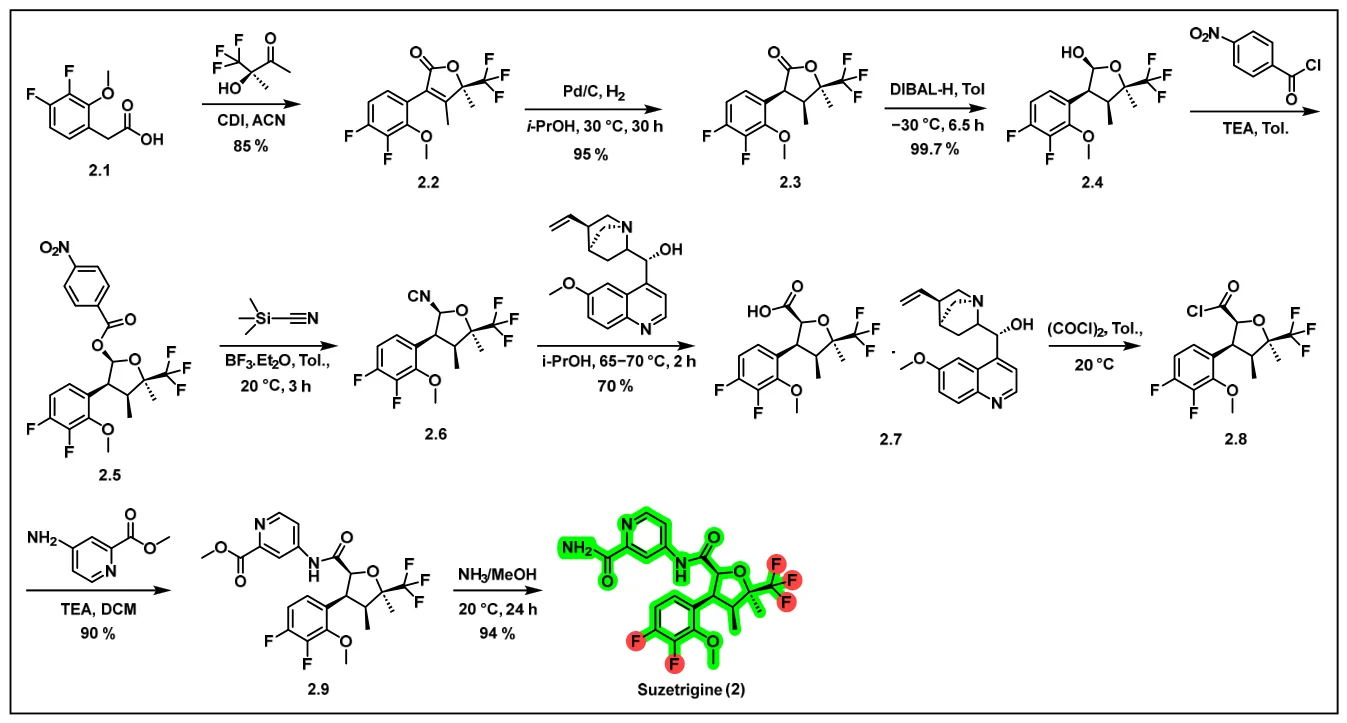
Synthesis of suzetrigine.

**Figure 4:**
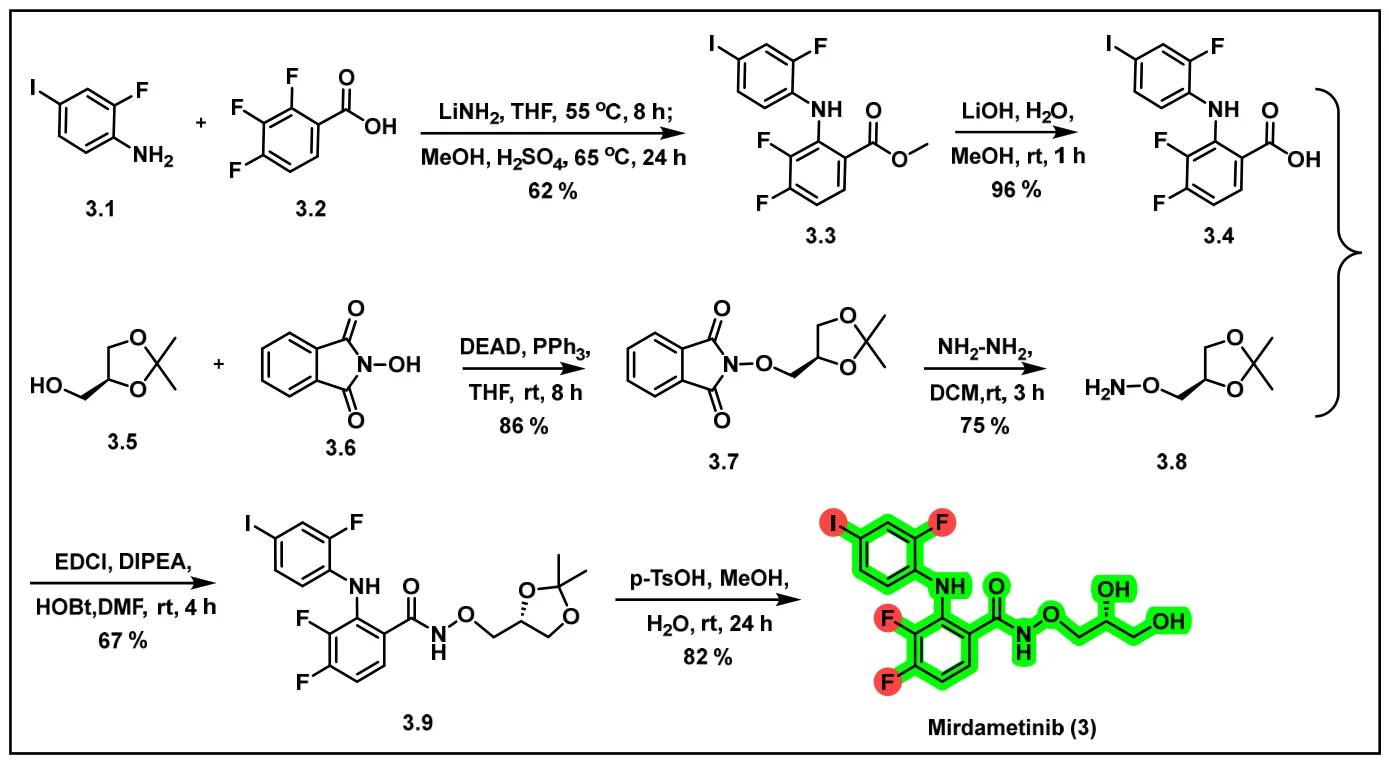
Synthesis of mirdametinib.

**Figure 5:**
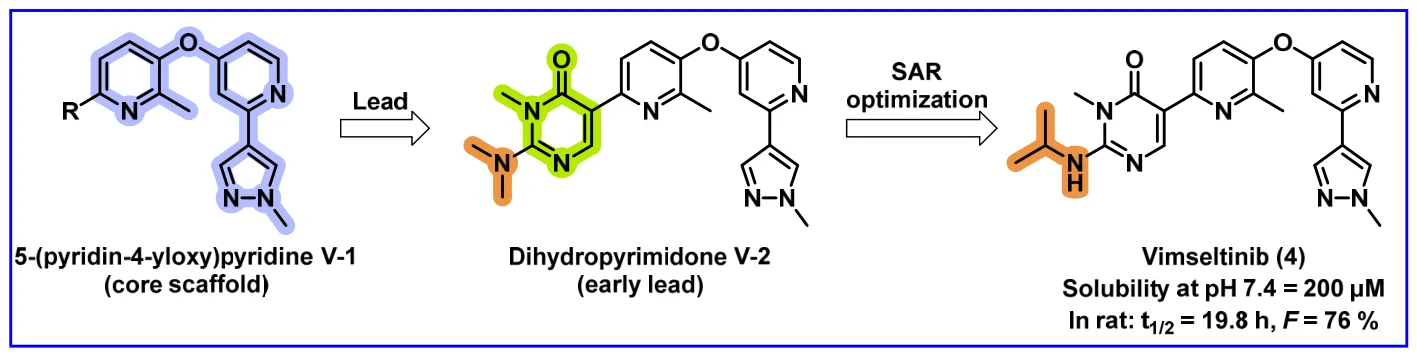
Optimization of vimseltinib.

**Figure 6:**
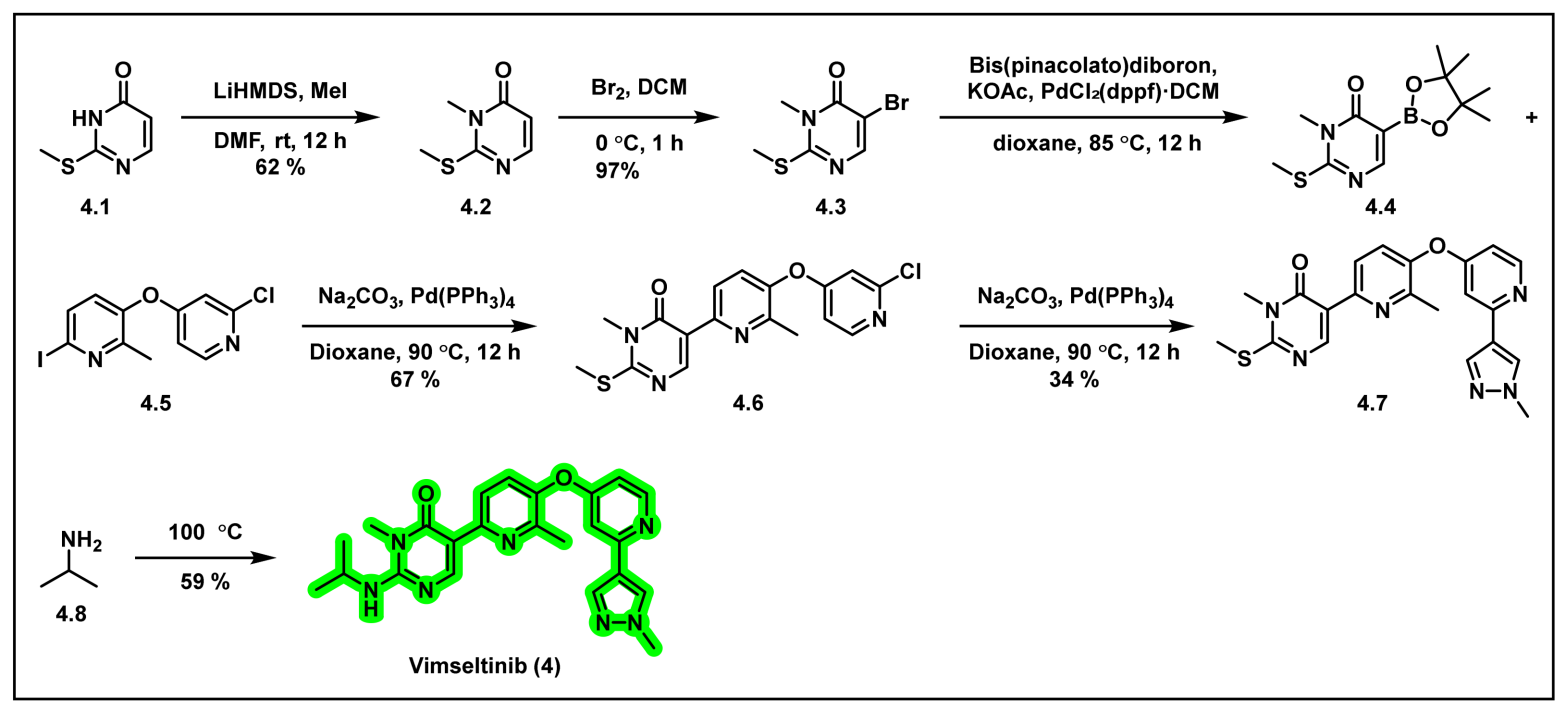
Synthesis of vimseltinib.

**Figure 7:**
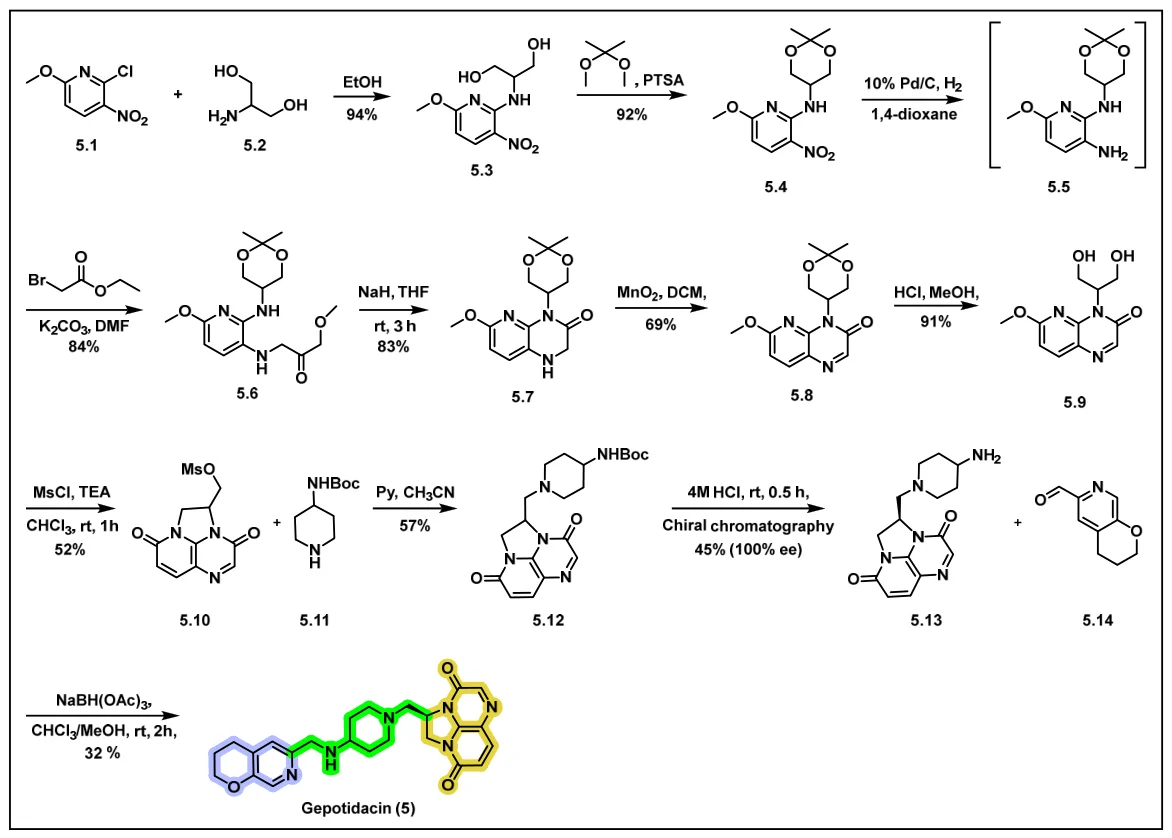
Synthesis of gepotidacin.

**Figure 8:**
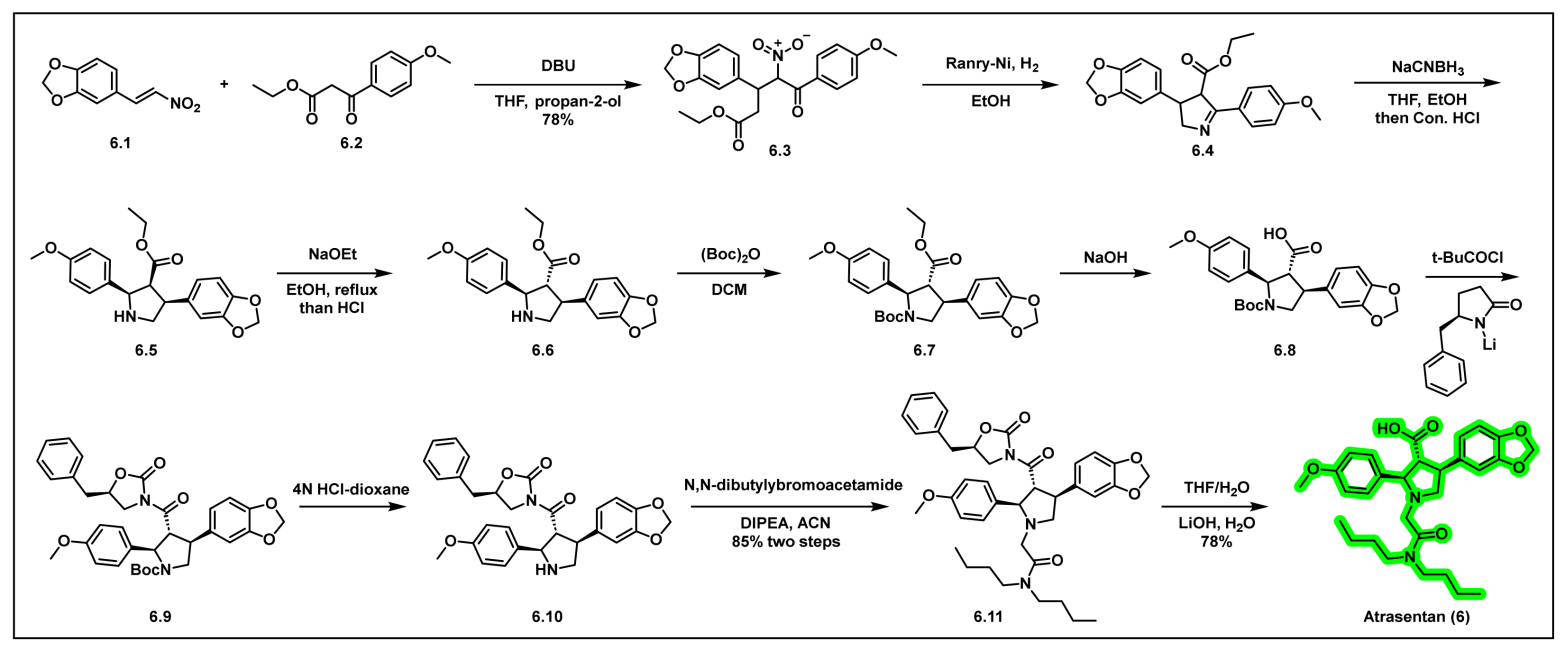
Synthesis of atrasentan.

**Figure 9:**
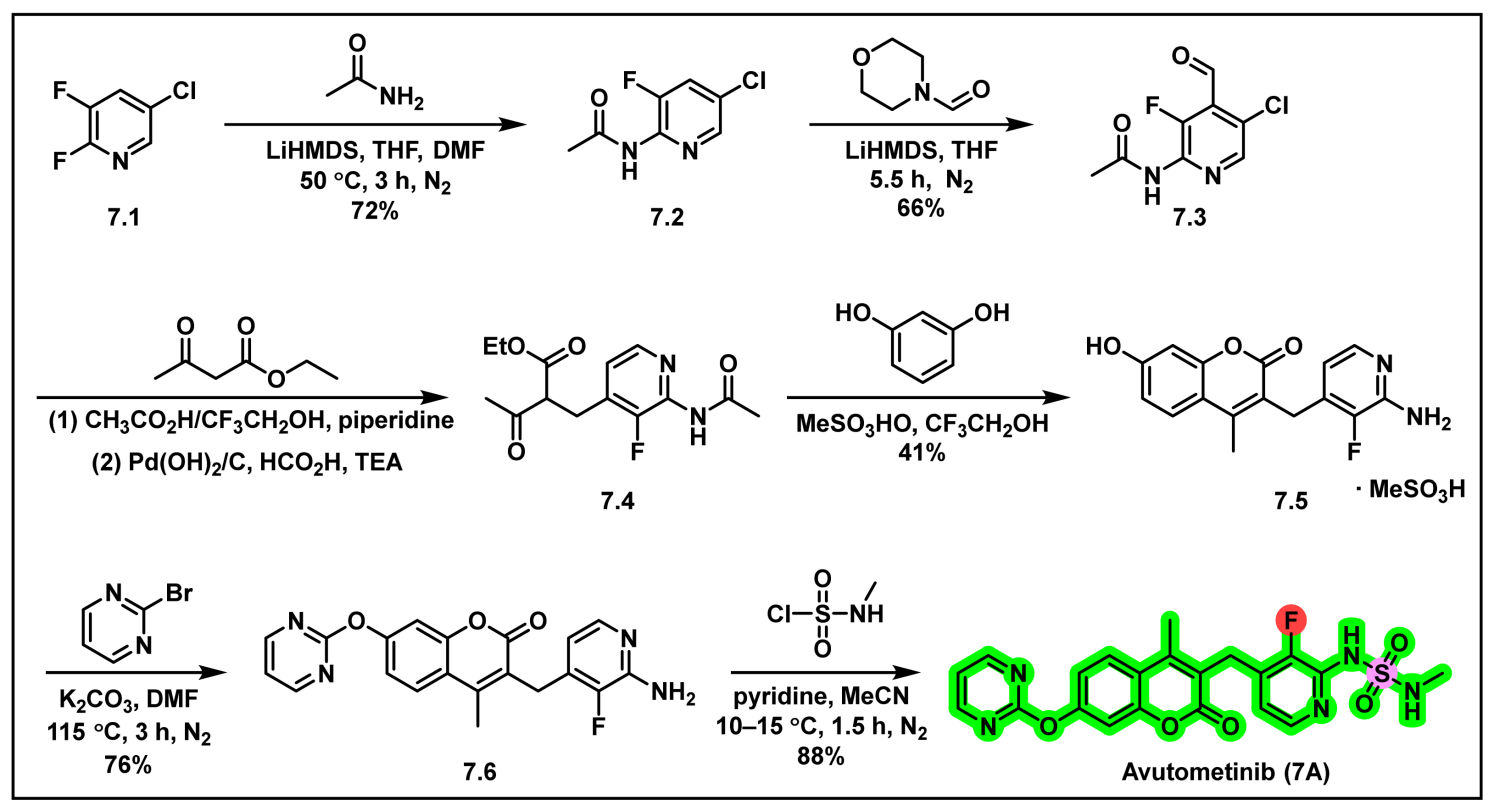
Synthesis of avutometinib.

**Figure 10:**
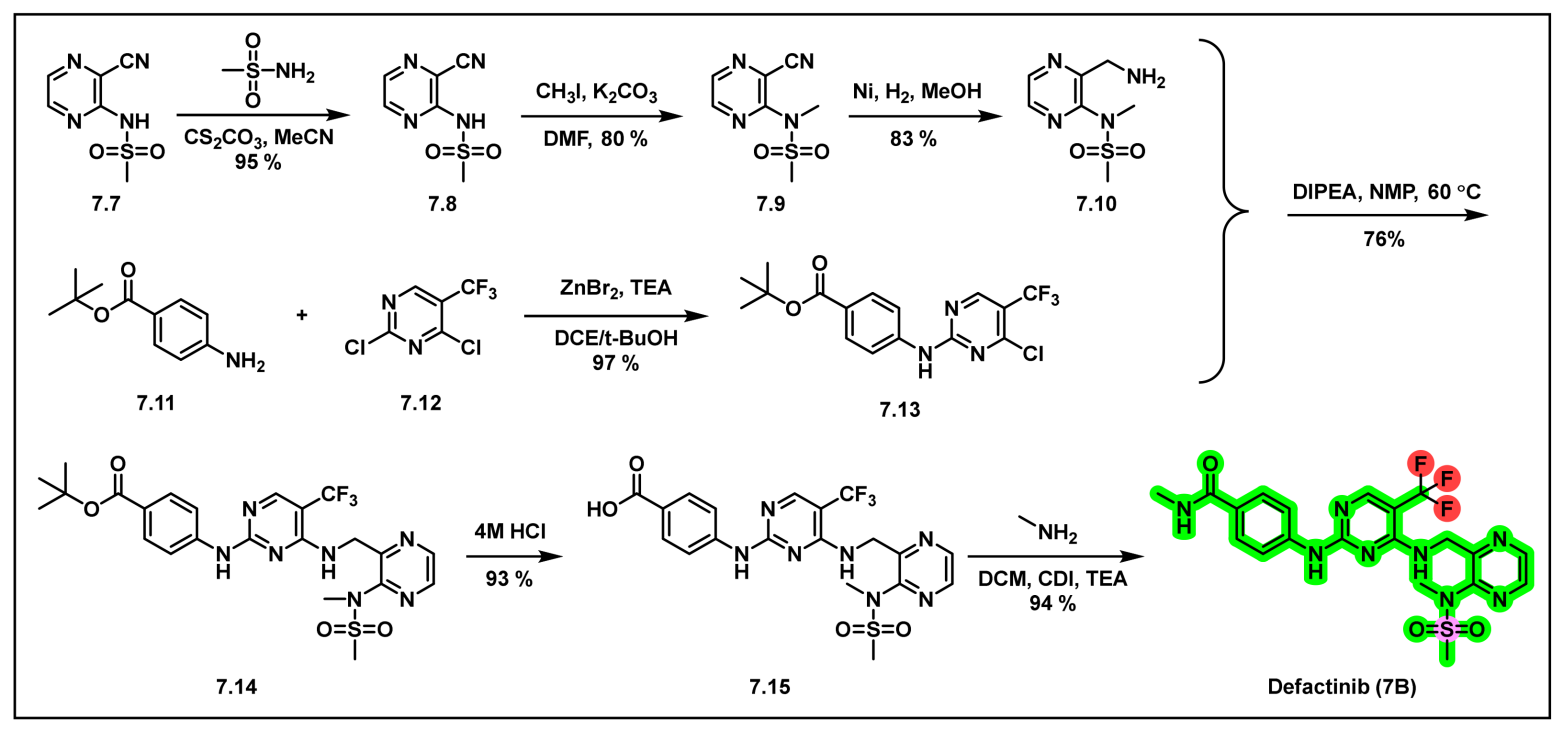
Synthesis of defactinib.

**Figure 11:**
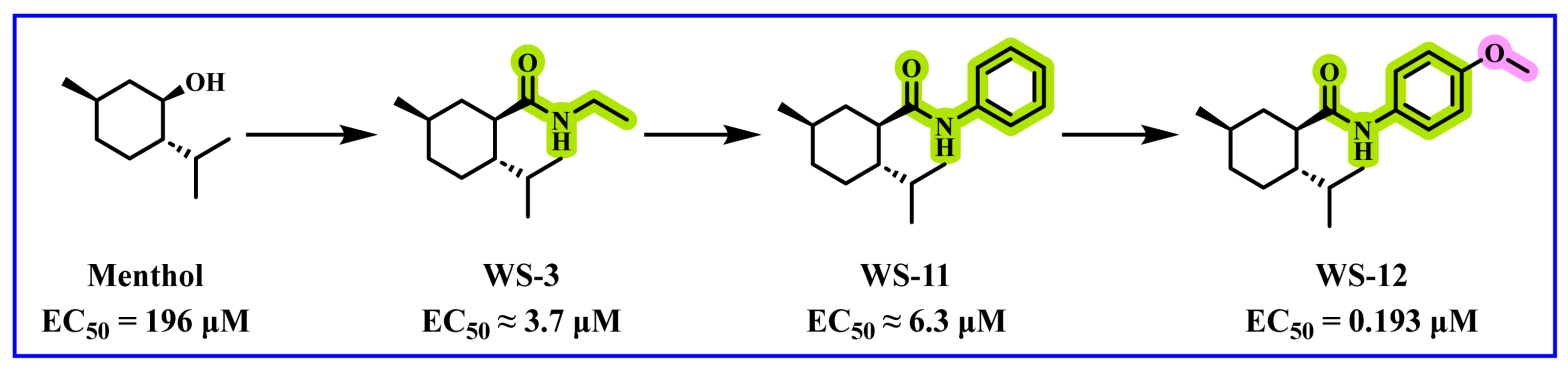
Discovery of WS-12.

**Figure 12:**
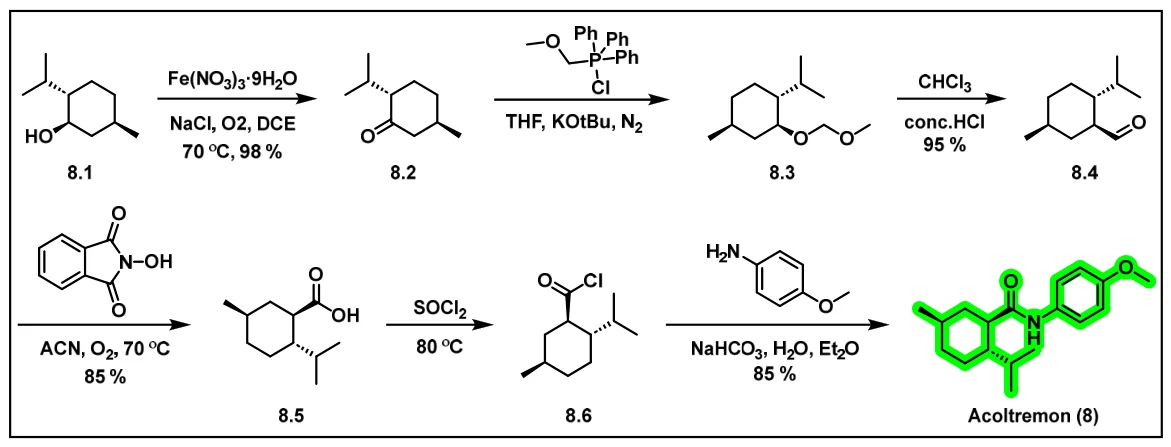
Synthesis of acoltremon.

**Figure 13:**
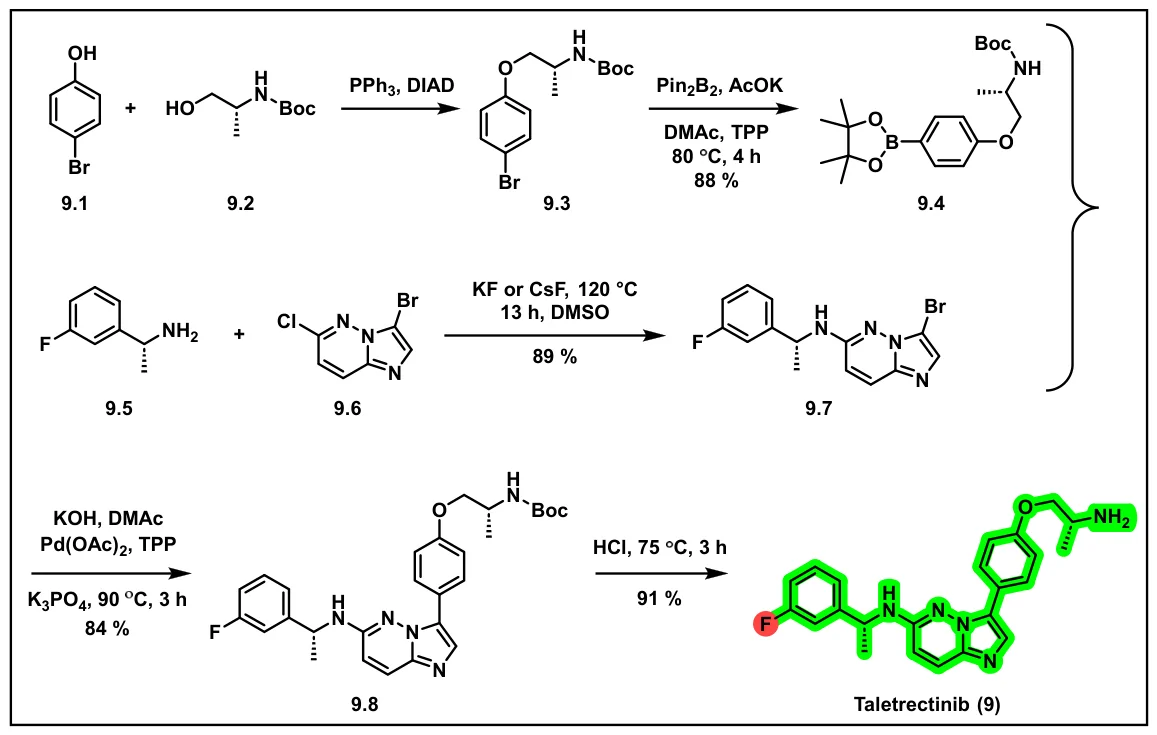
Synthesis of taletrectinib.

**Figure 14:**
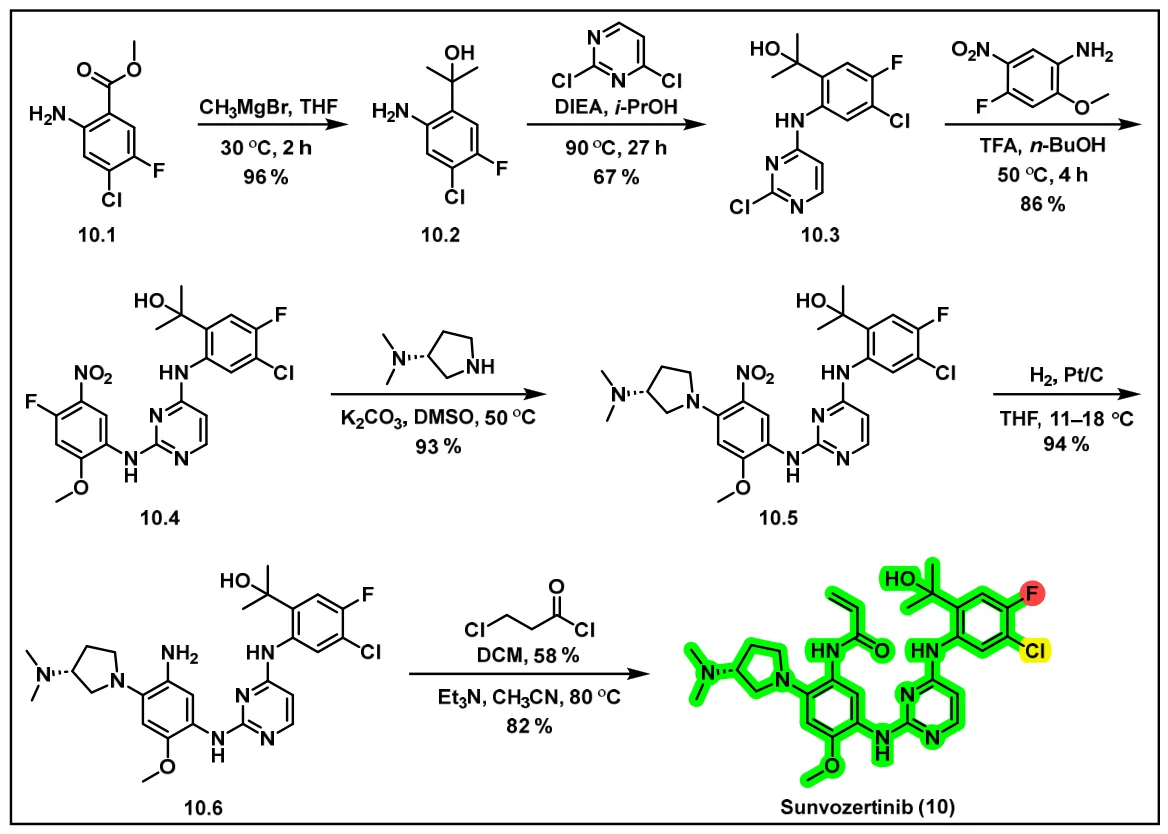
Synthesis of sunvozertinib.

**Figure 15:**
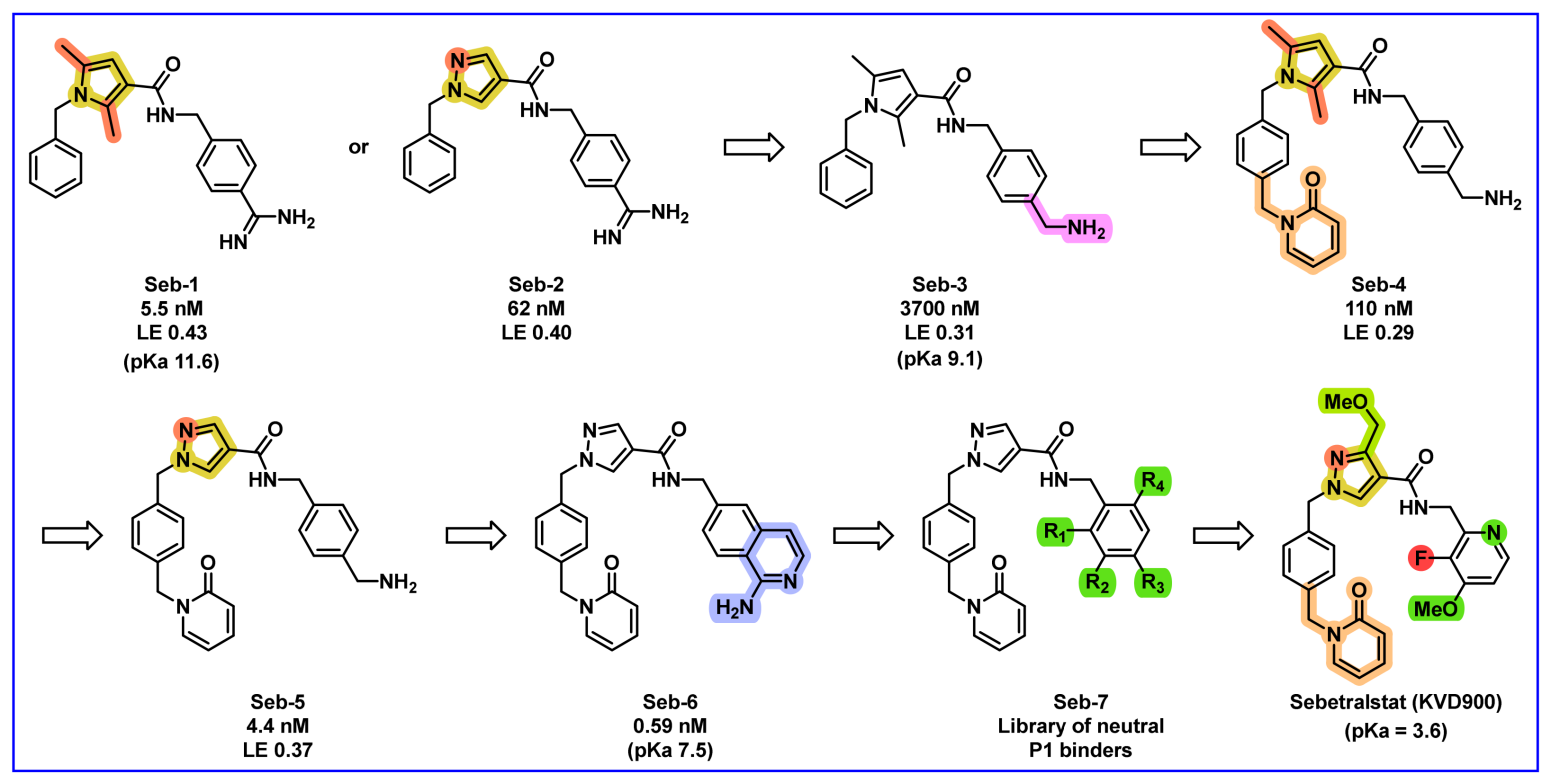
Optimization of sebetralstat.

**Figure 16:**
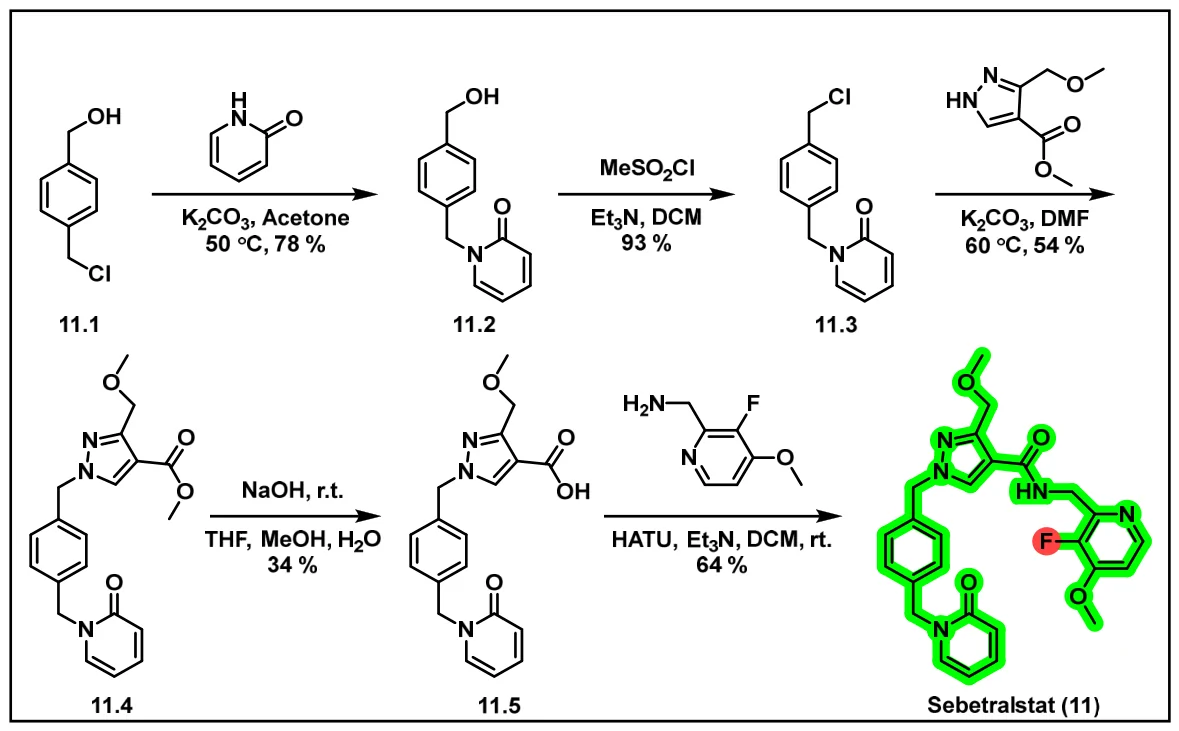
Synthesis of sebetralstat.

**Figure 17:**
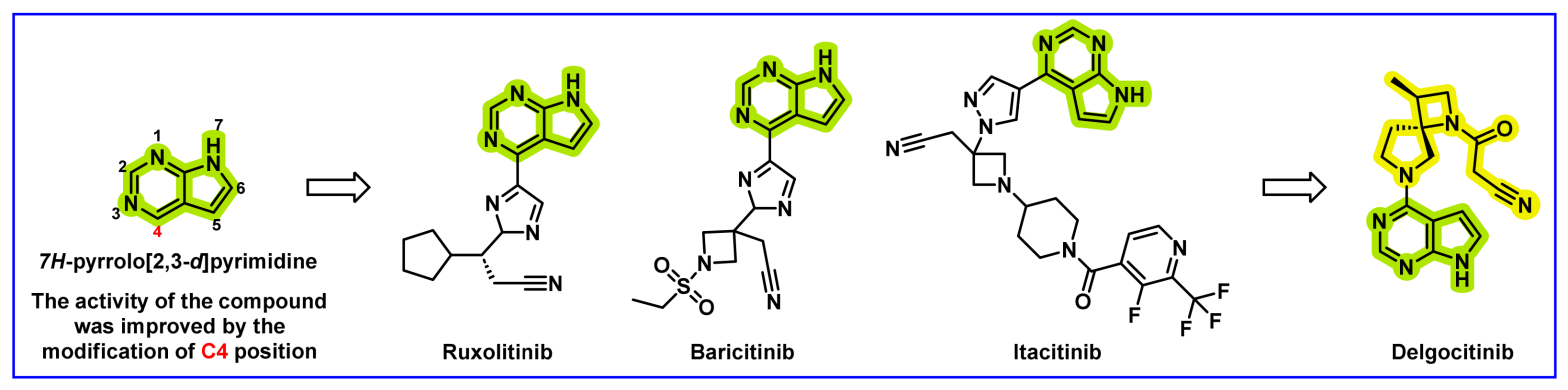
Optimization of delgocitinib.

**Figure 18:**
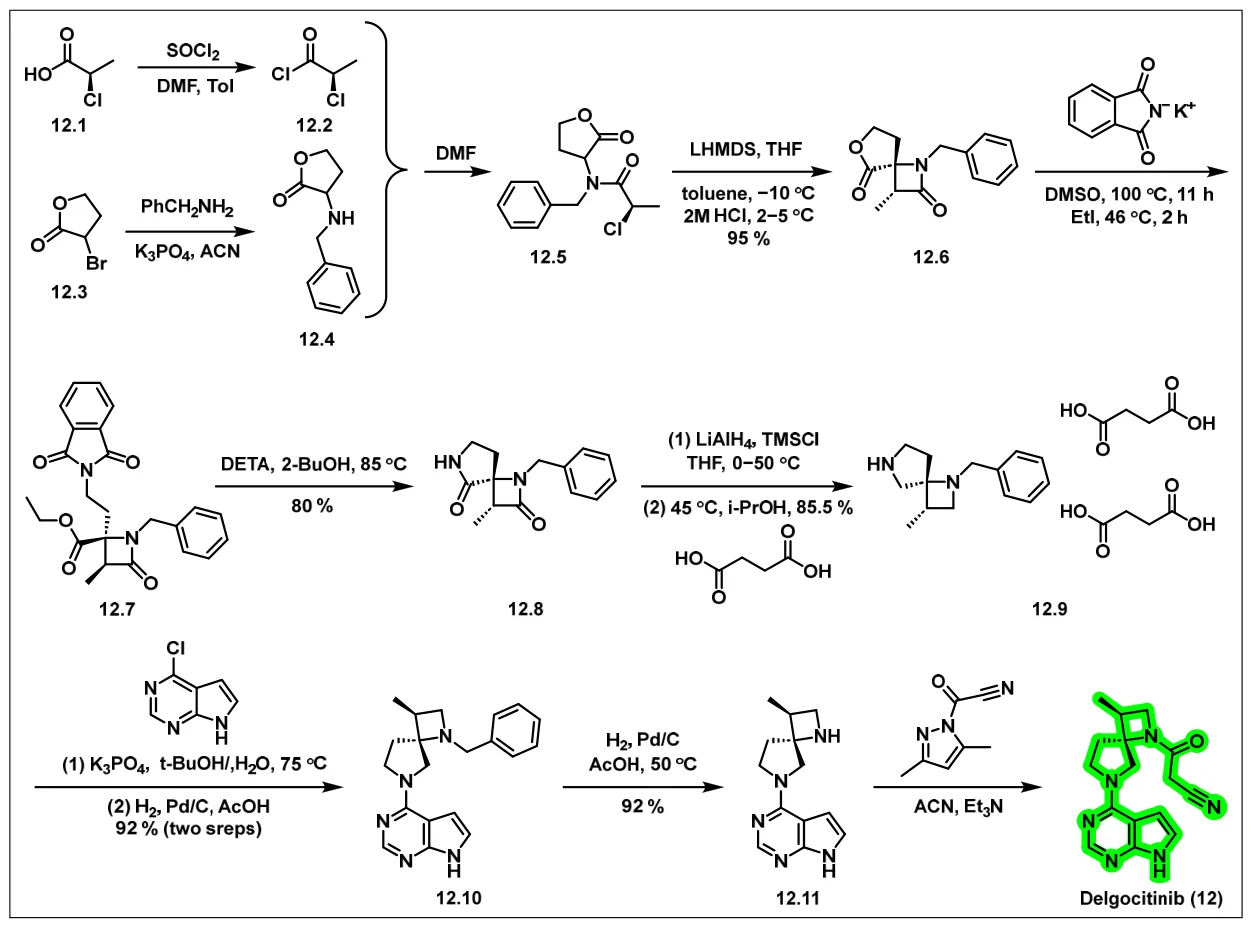
Synthesis of delgocitinib.

**Figure 19:**
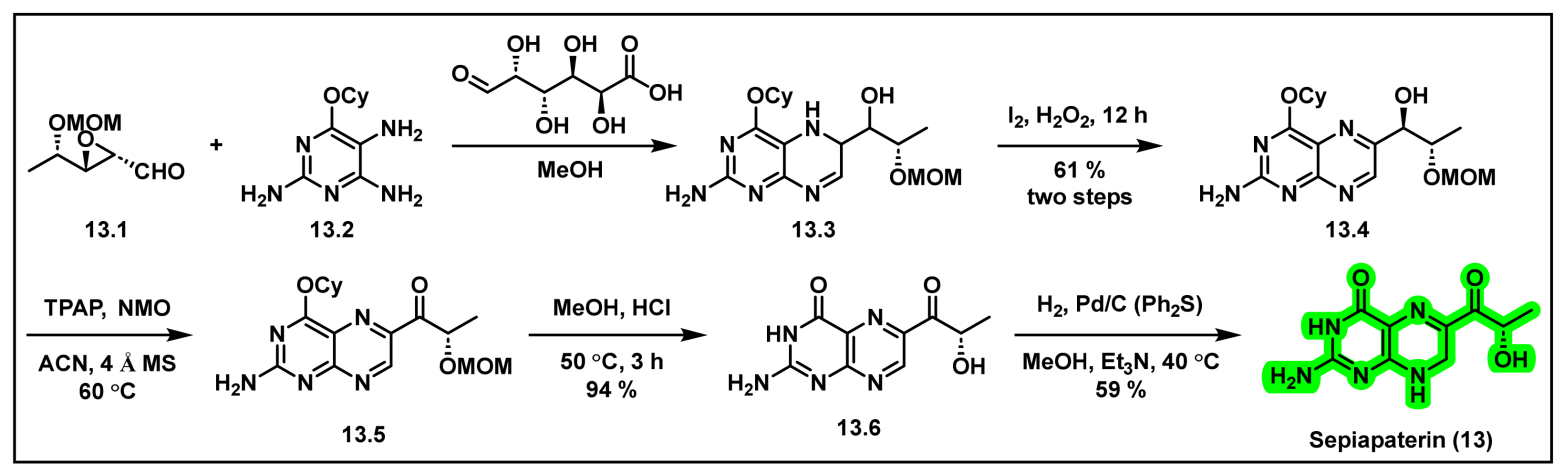
Synthesis of sepiapterin.

**Figure 20:**
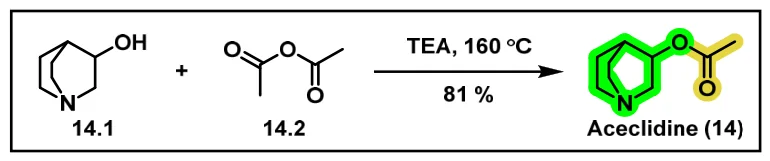
Synthesis of aceclidine.

**Figure 21:**
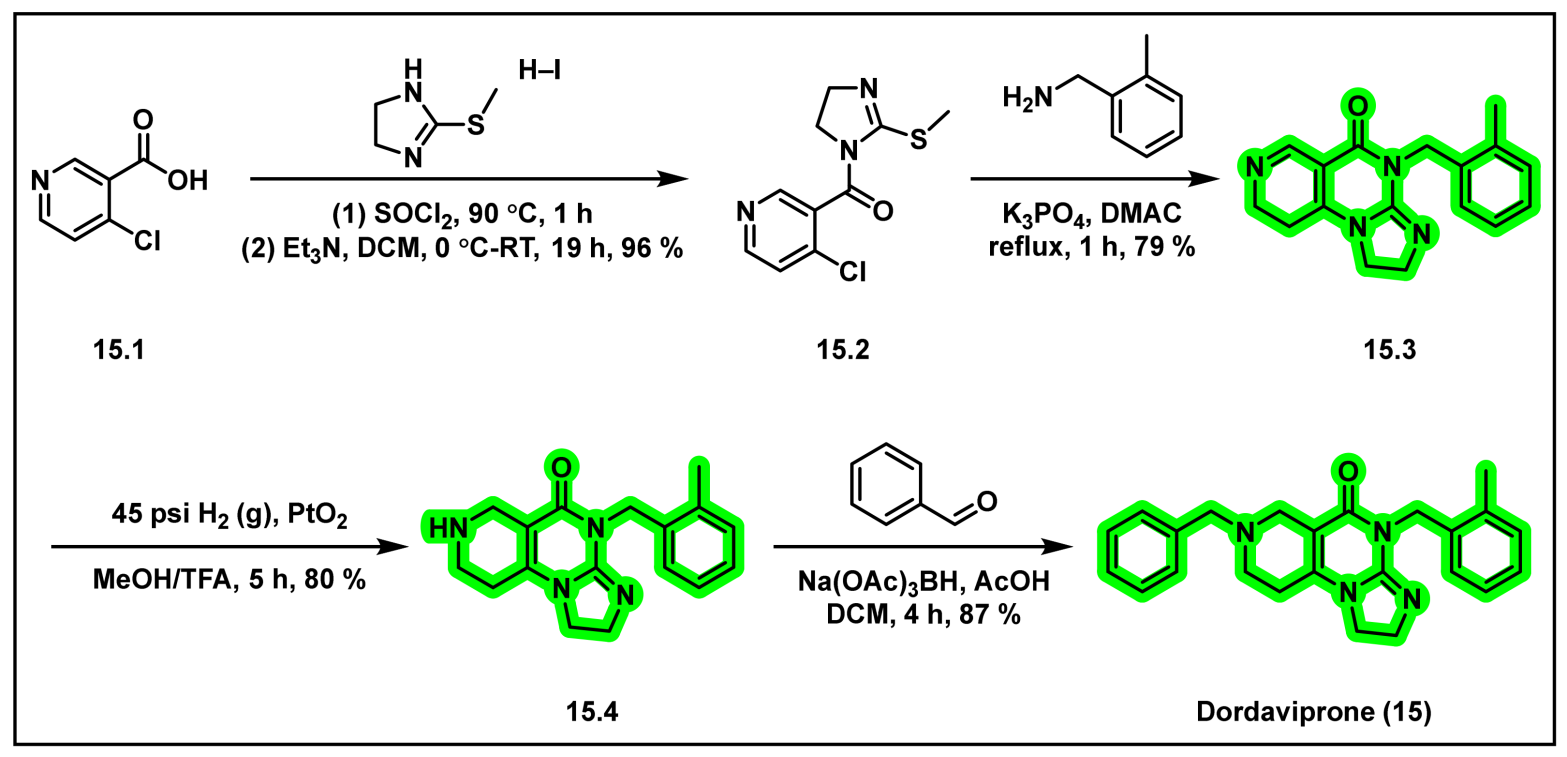
Synthesis of dordaviprone.

**Figure 22:**
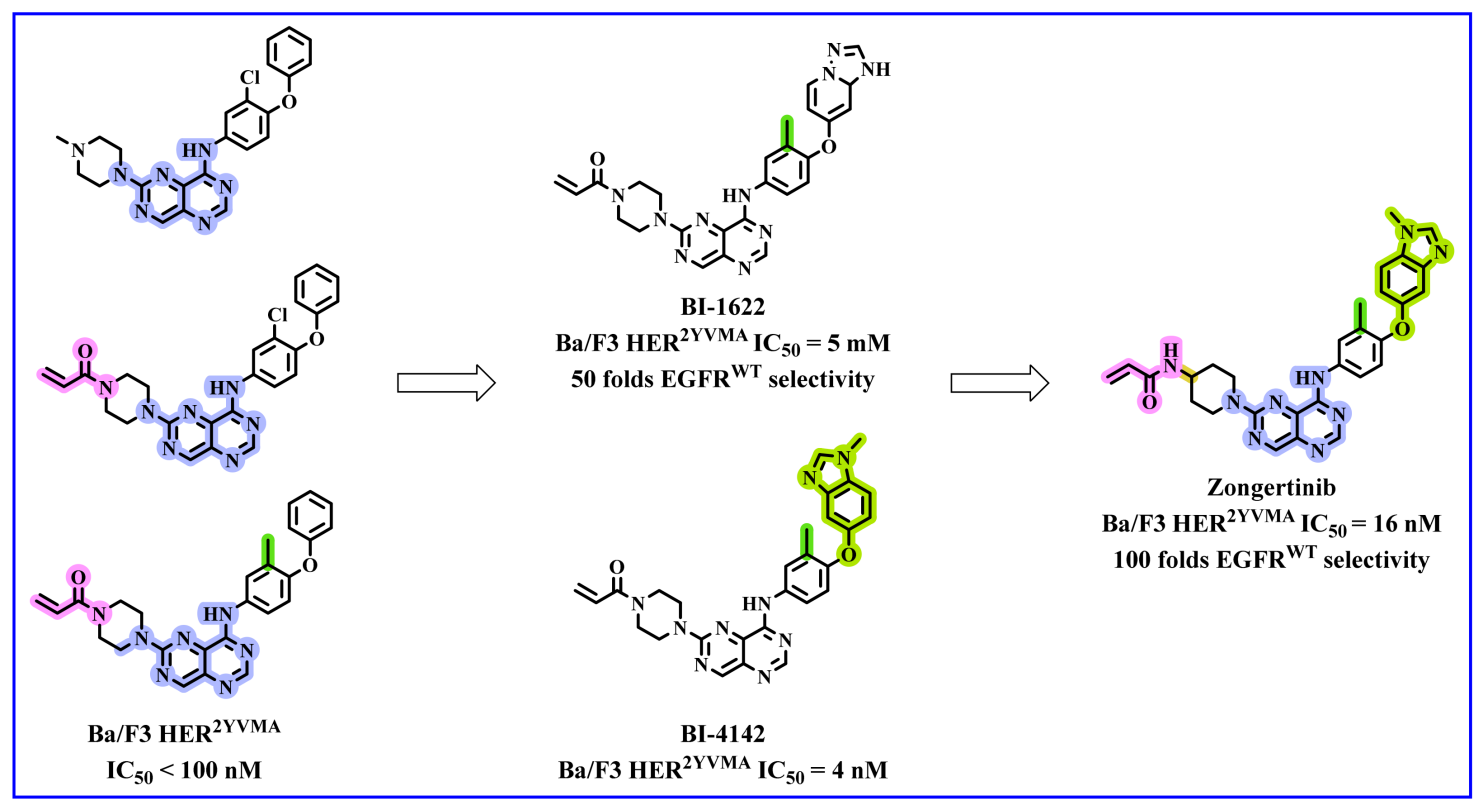
Optimization of zongertinib.

**Figure 23:**
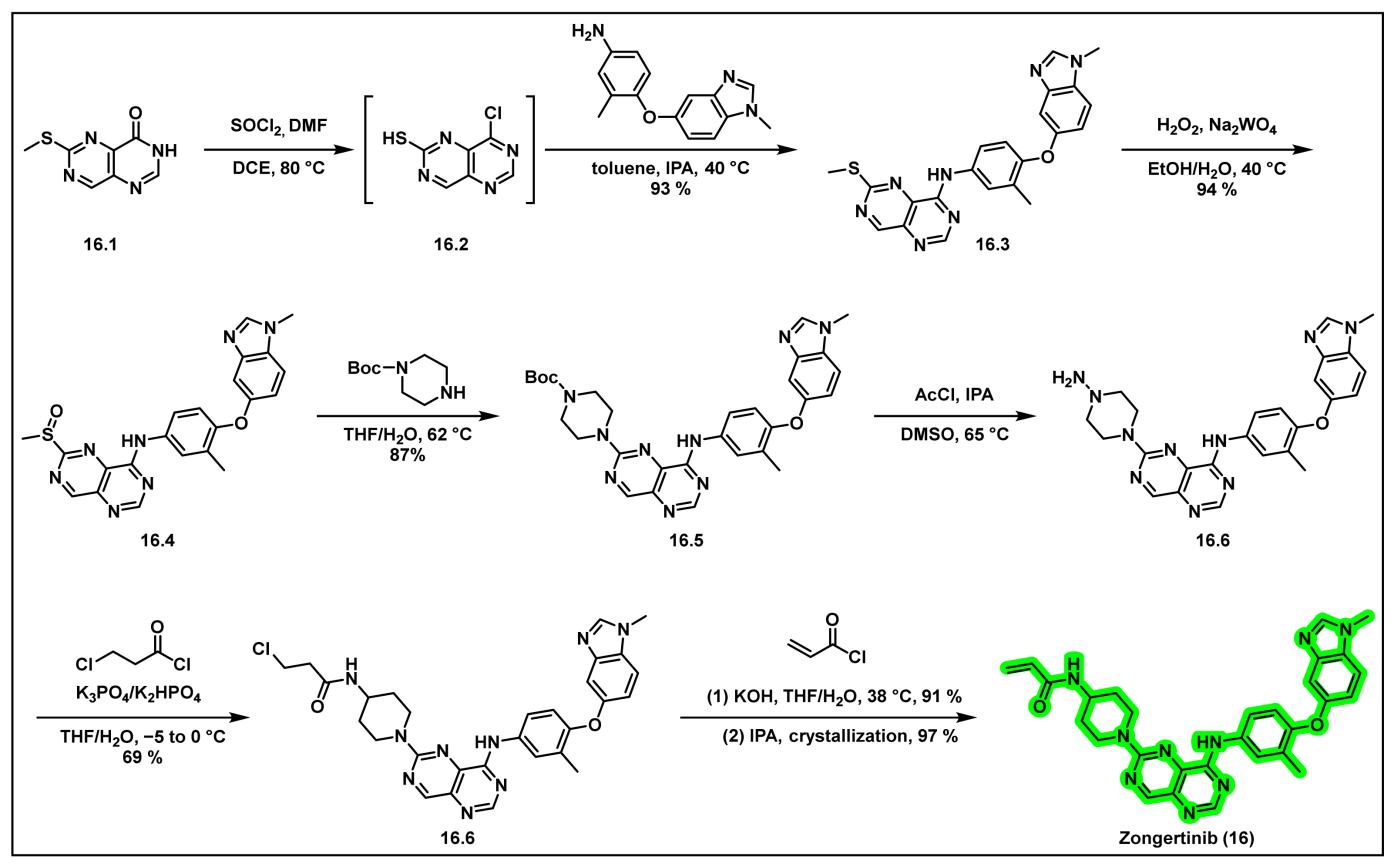
Synthesis of zongertinib.

**Figure 24:**
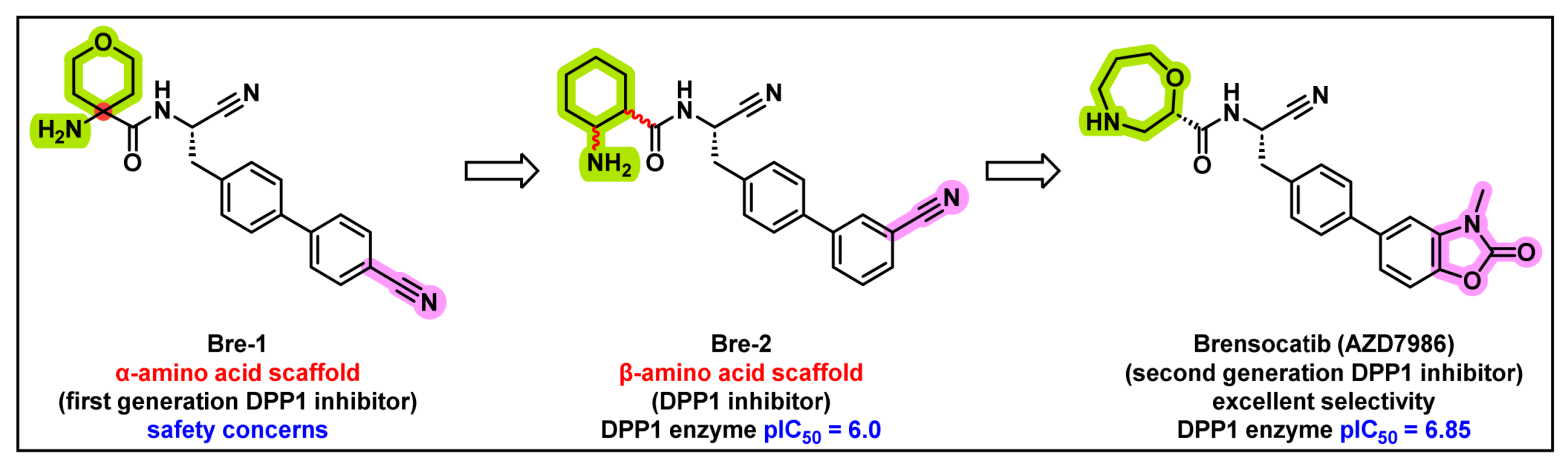
Optimization of brensocatib.

**Figure 25:**
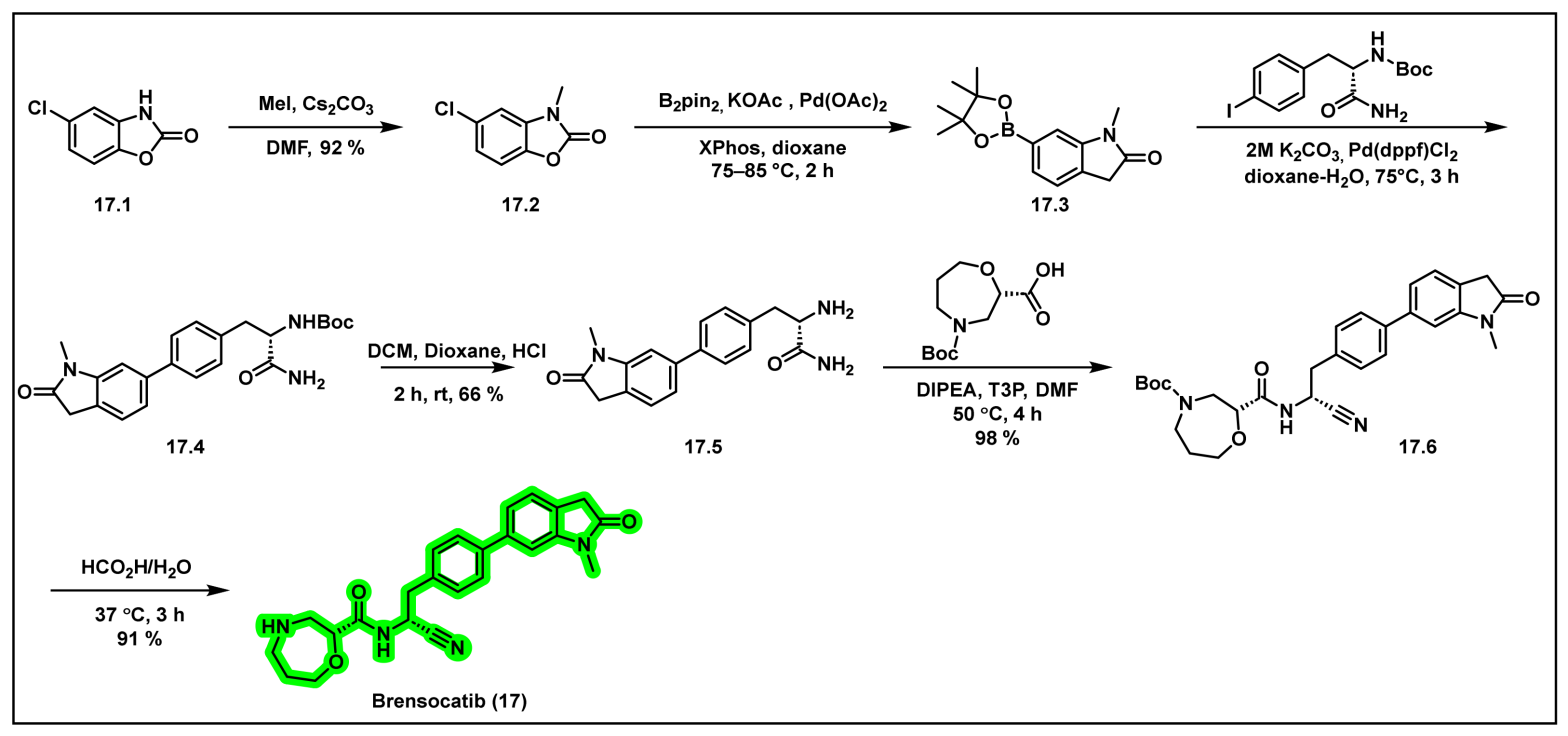
Synthesis of brensocatib.

**Figure 26:**
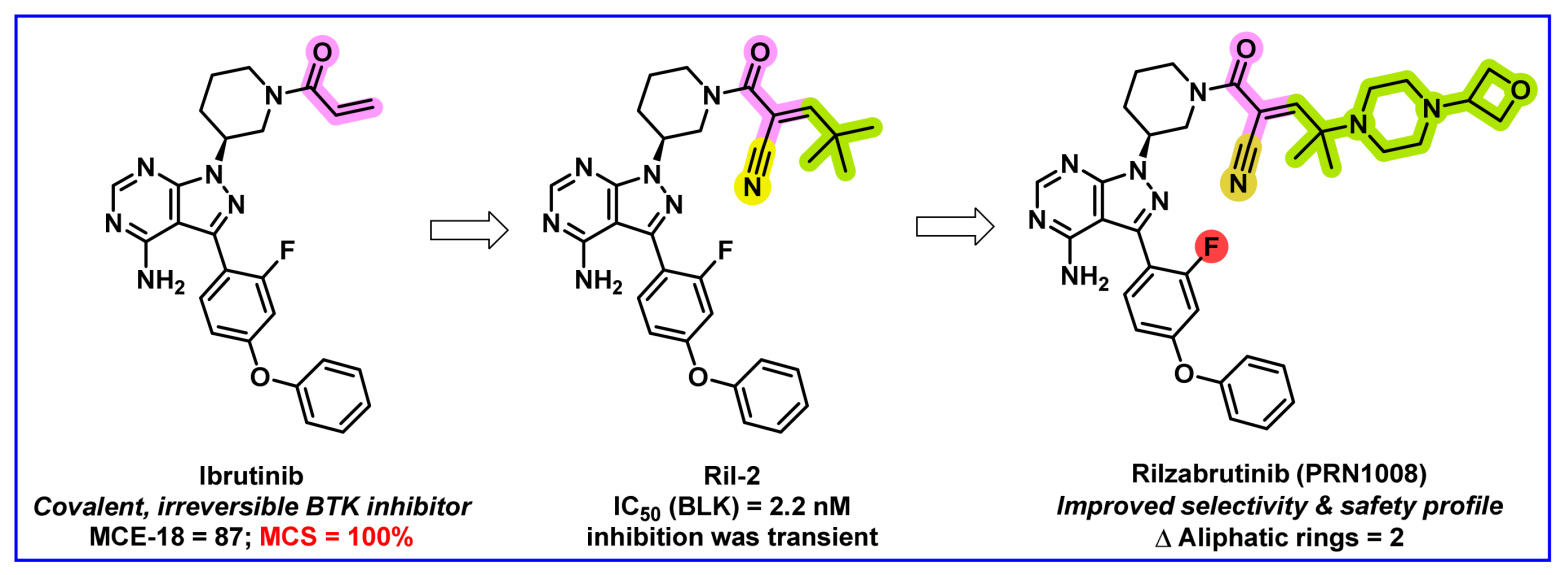
Optimization of rilzabrutinib.

**Figure 27:**
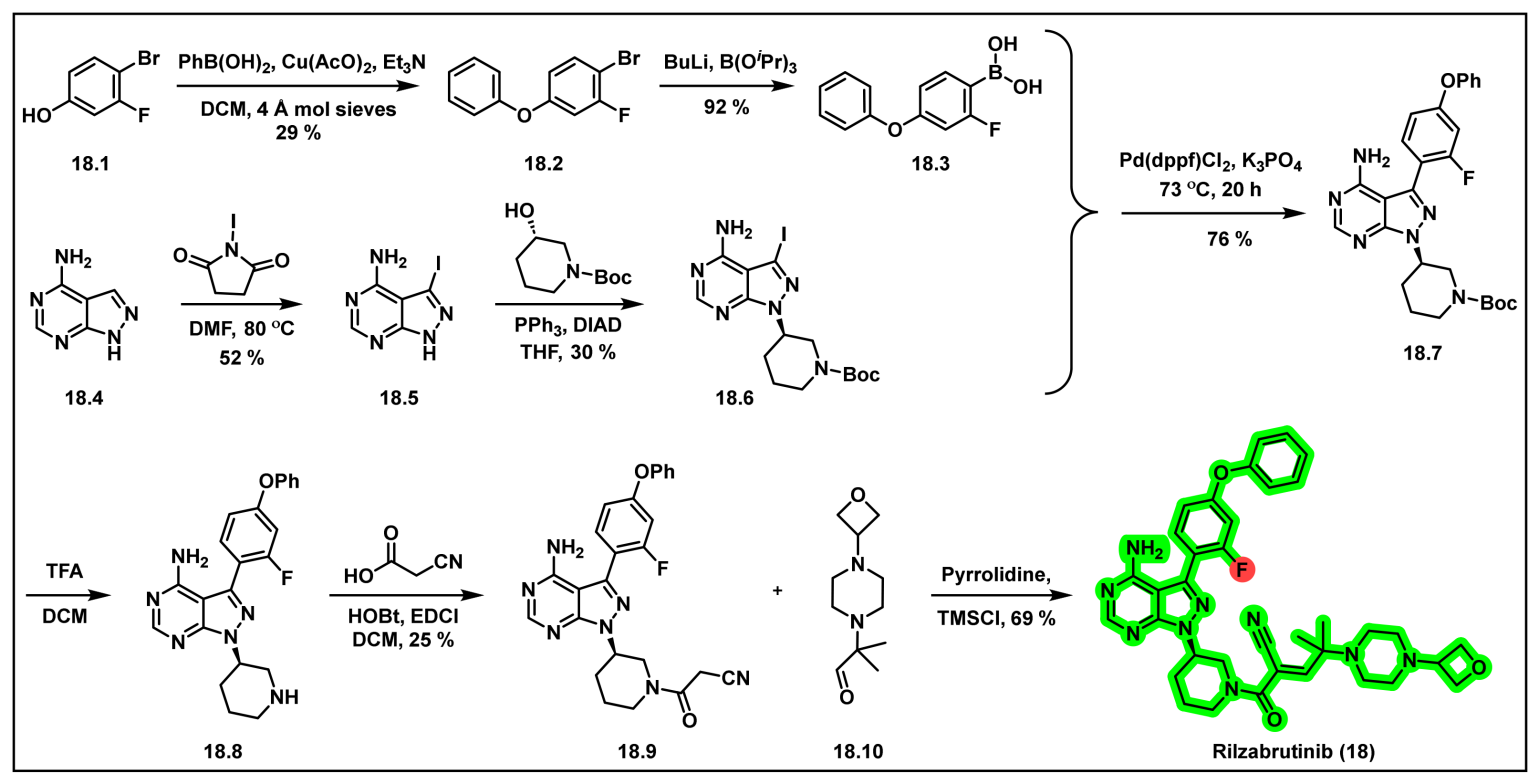
Synthesis of rilzabrutinib.

**Figure 28:**
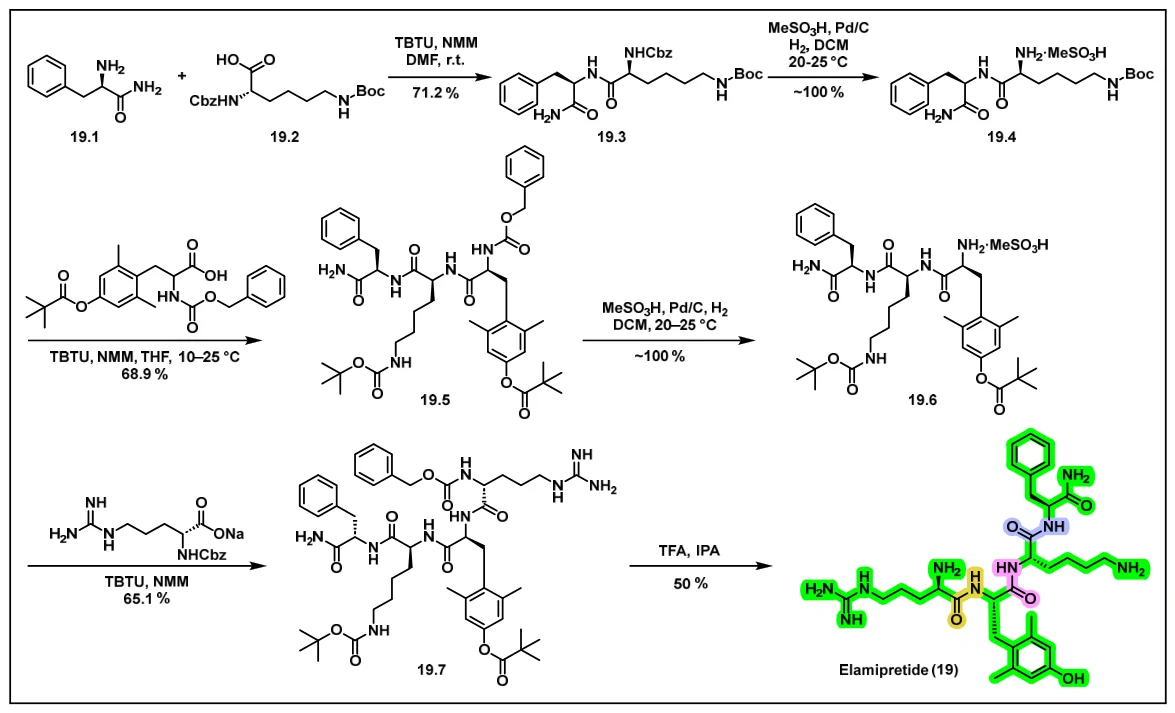
Synthesis of elamipretide.

**Figure 29:**
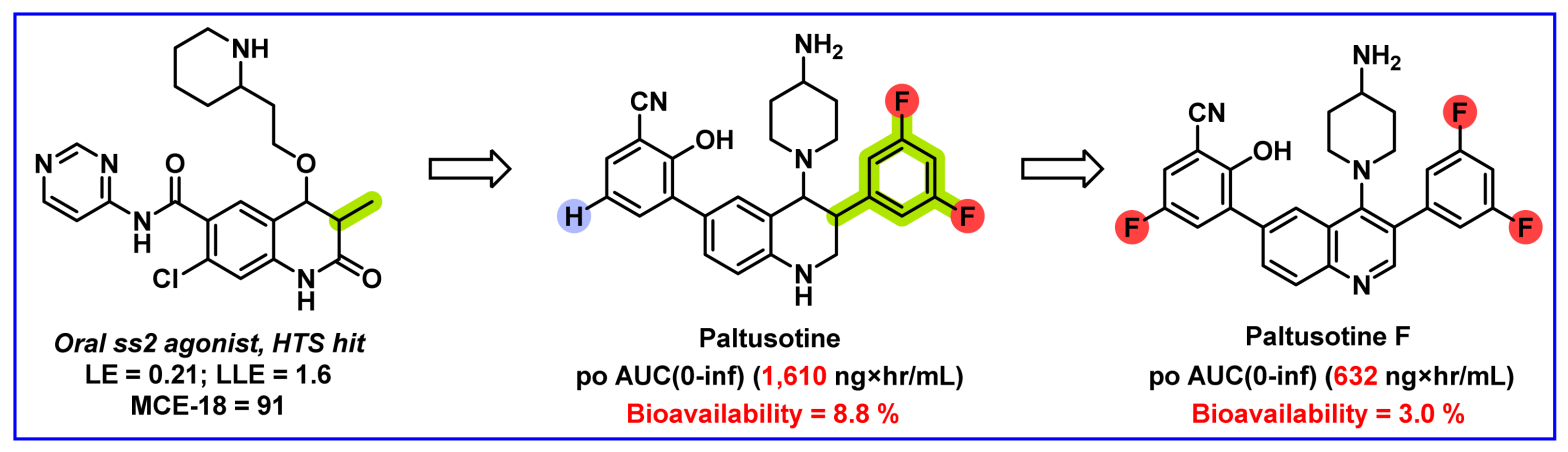
Optimization of paltusotine.

**Figure 30:**
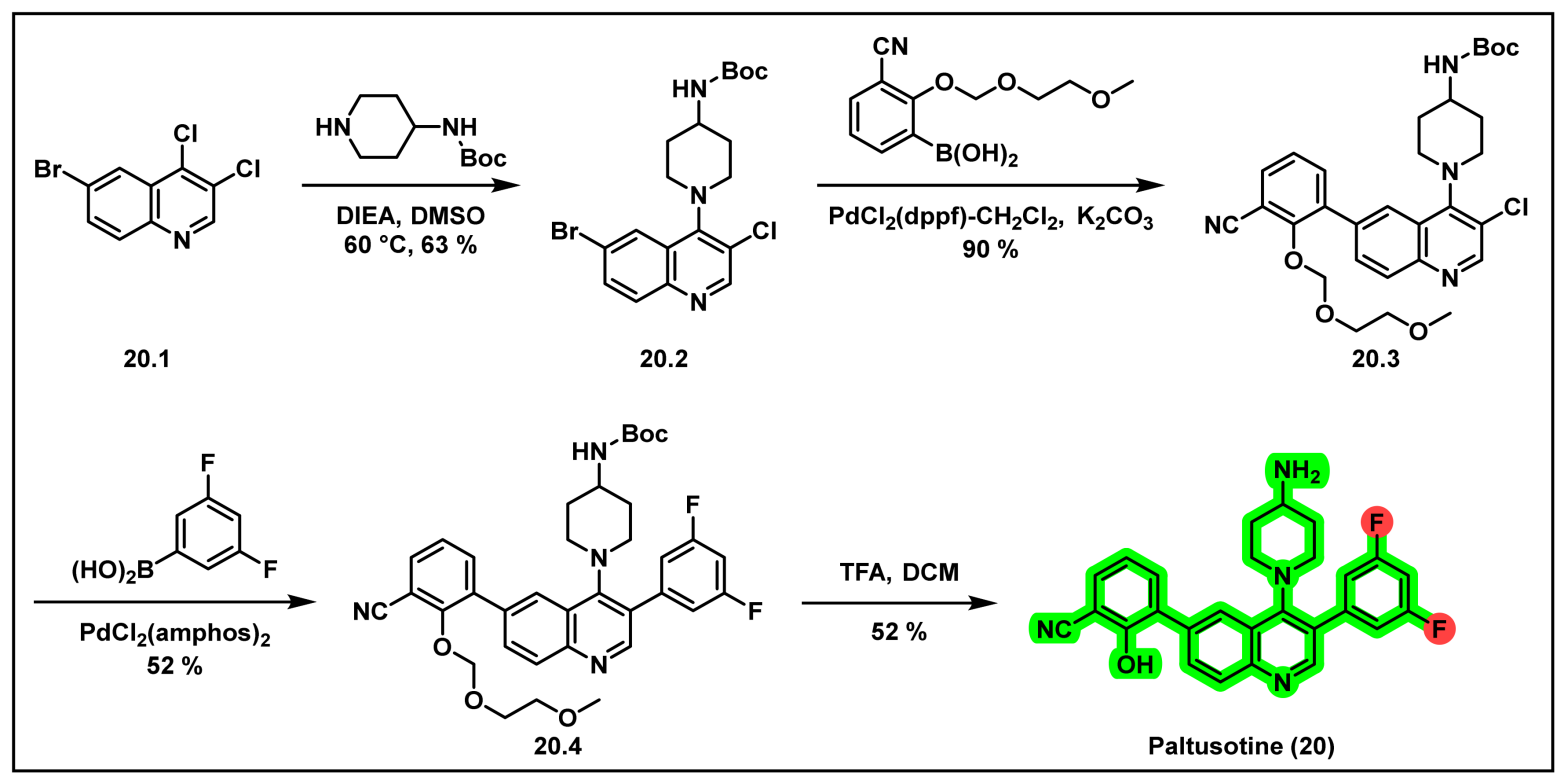
Synthesis of paltusotine.

**Figure 31:**
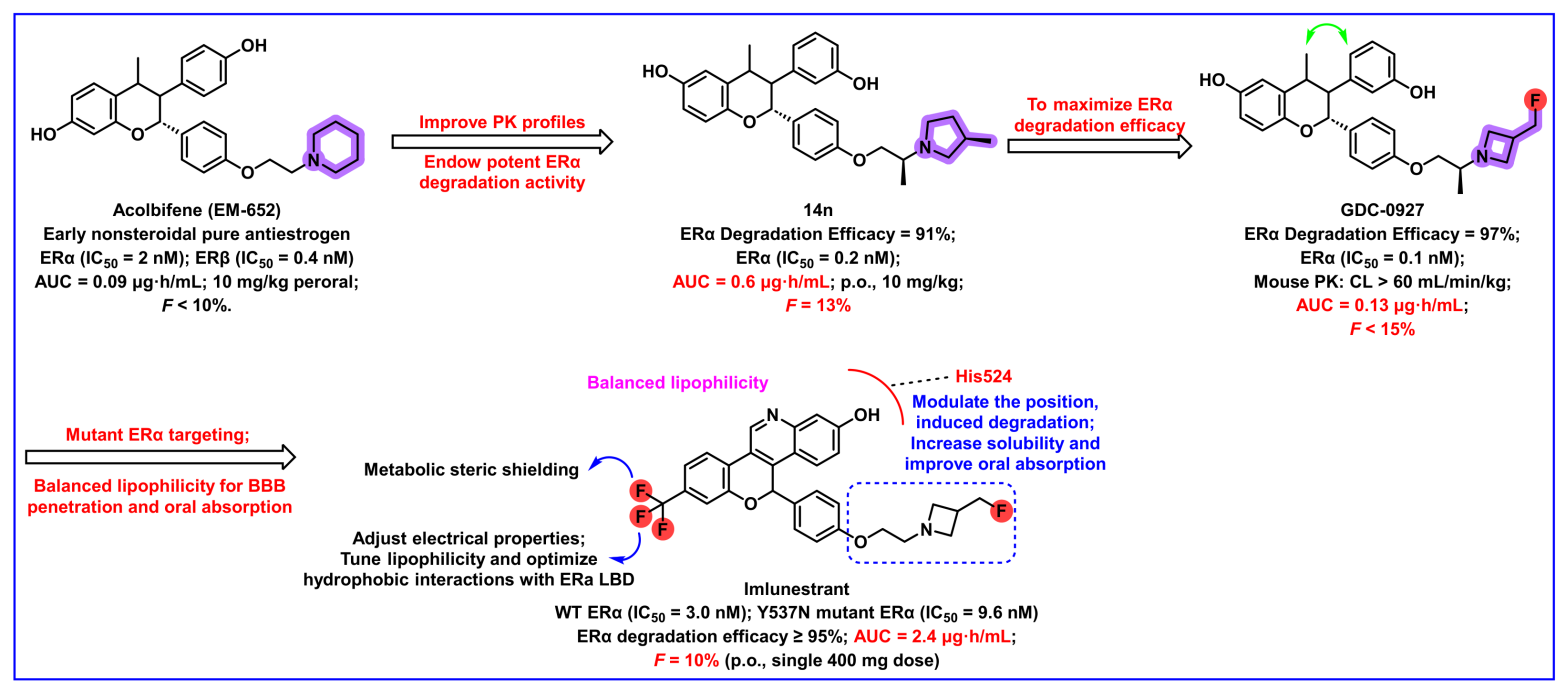
Optimization of imlunestrant.

**Figure 32:**
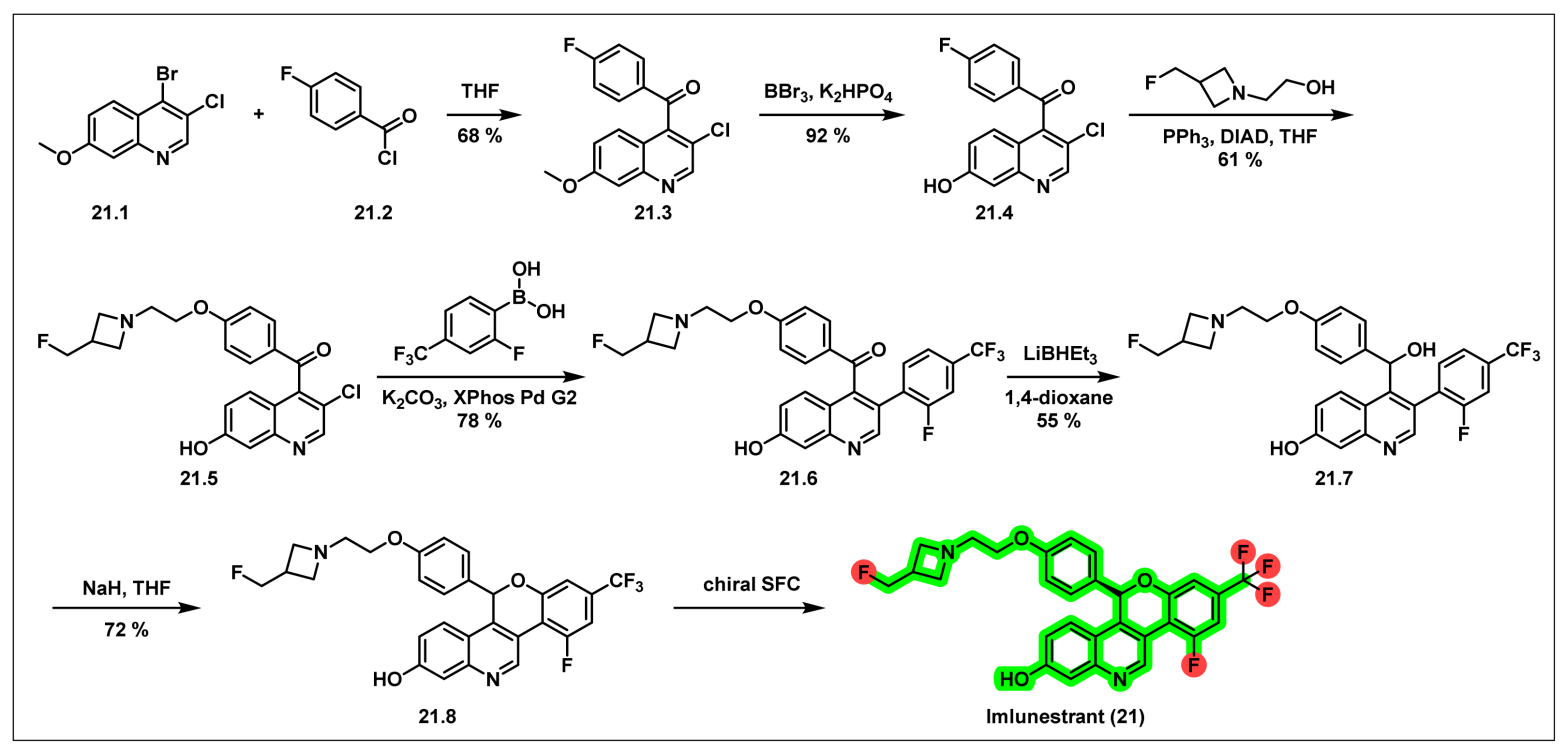
Synthesis of imlunestrant.

**Figure 33:**
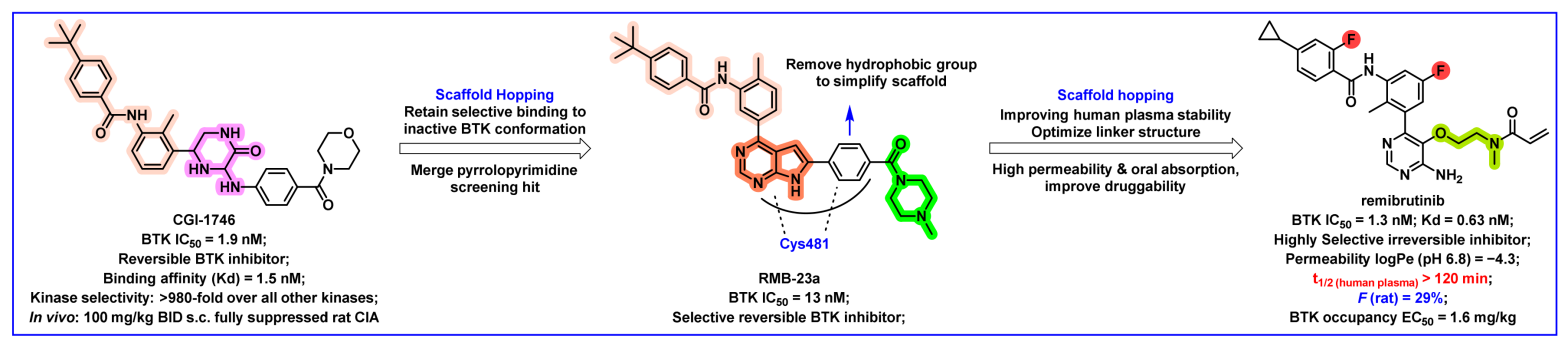
Optimization of remibrutinib.

**Figure 34:**
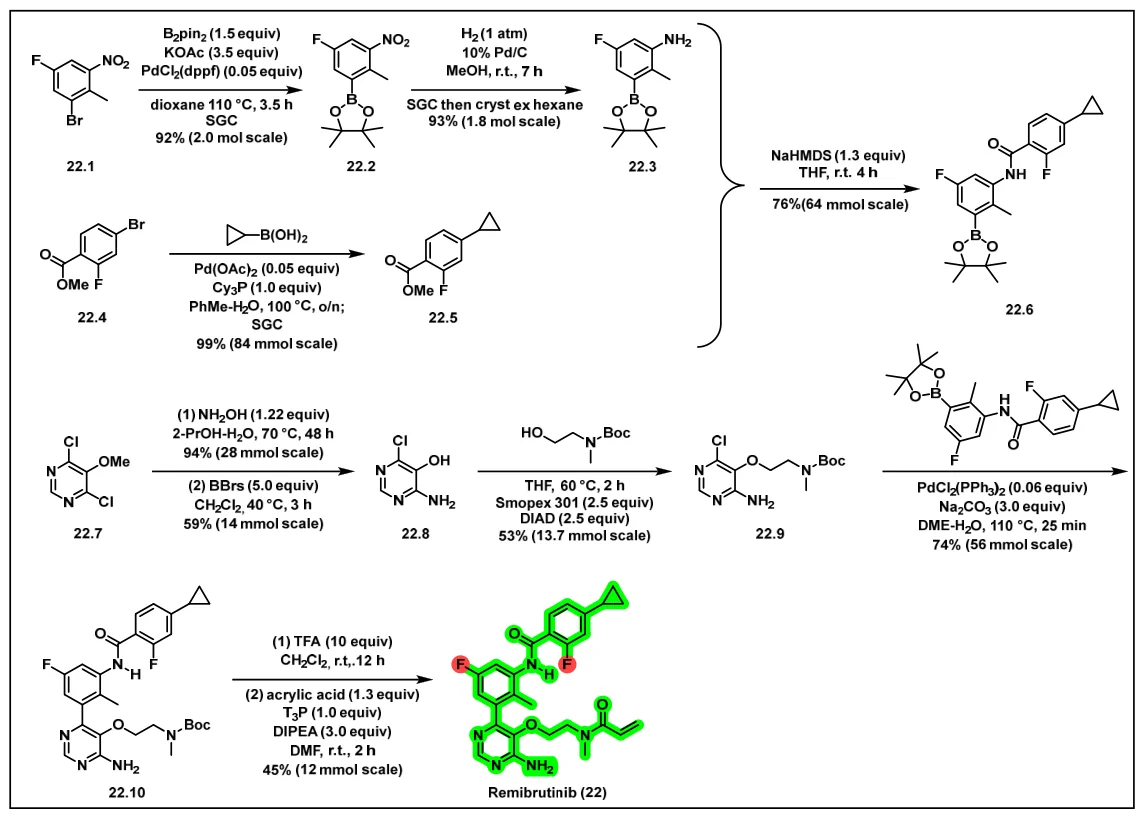
Synthesis of remibrutinib.

**Figure 35:**
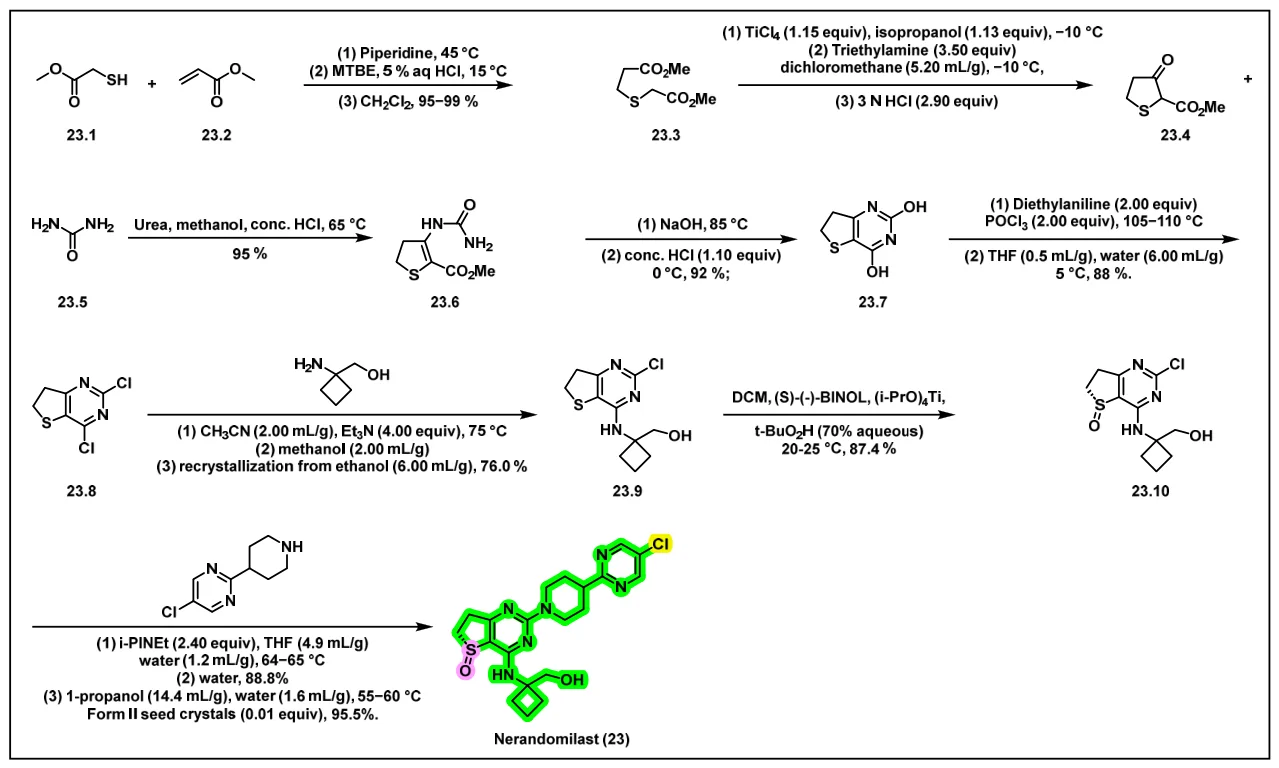
Synthesis of nerandomilast.

**Figure 36:**
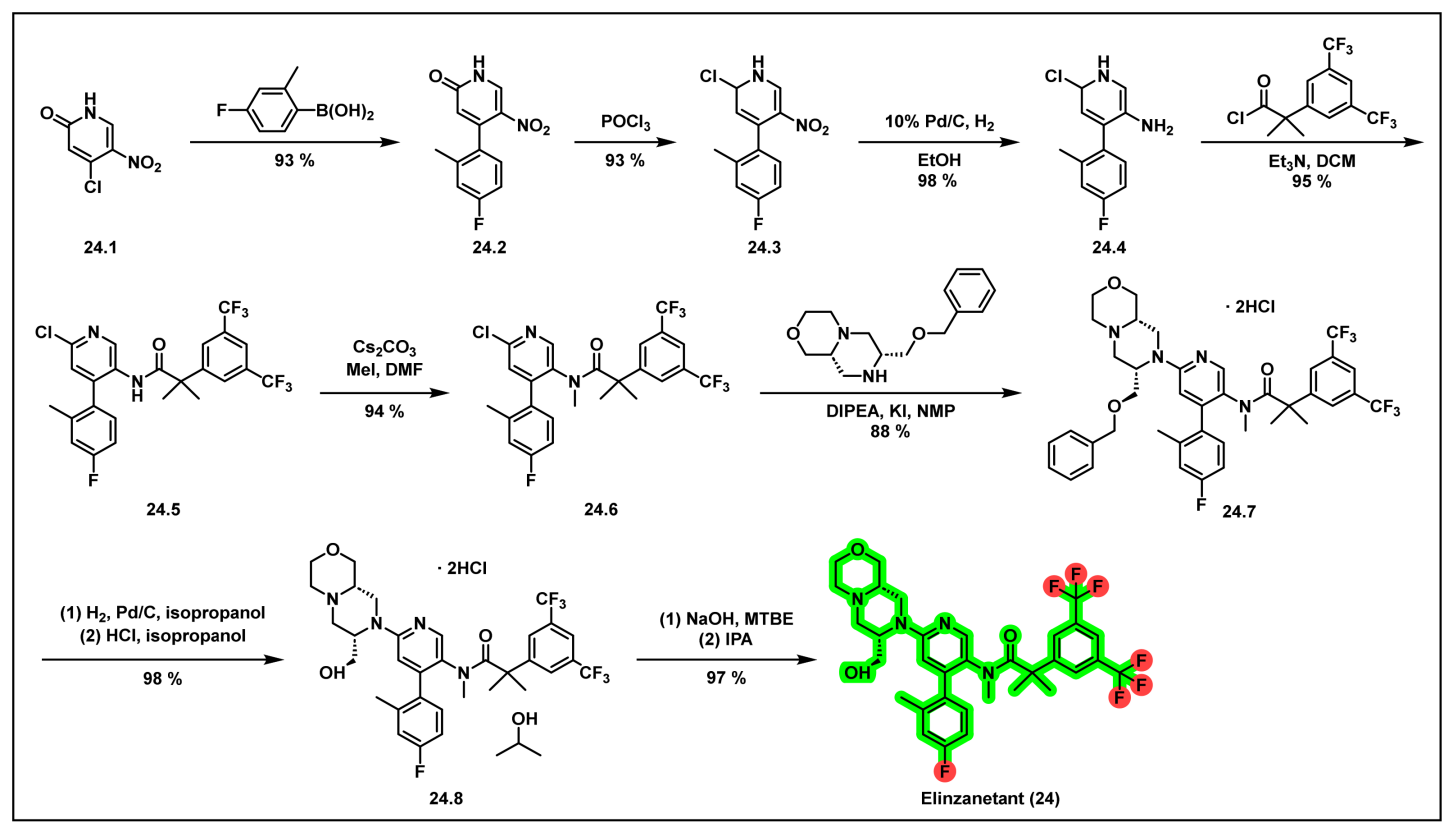
Synthesis of elinzanetant.

**Figure 37:**
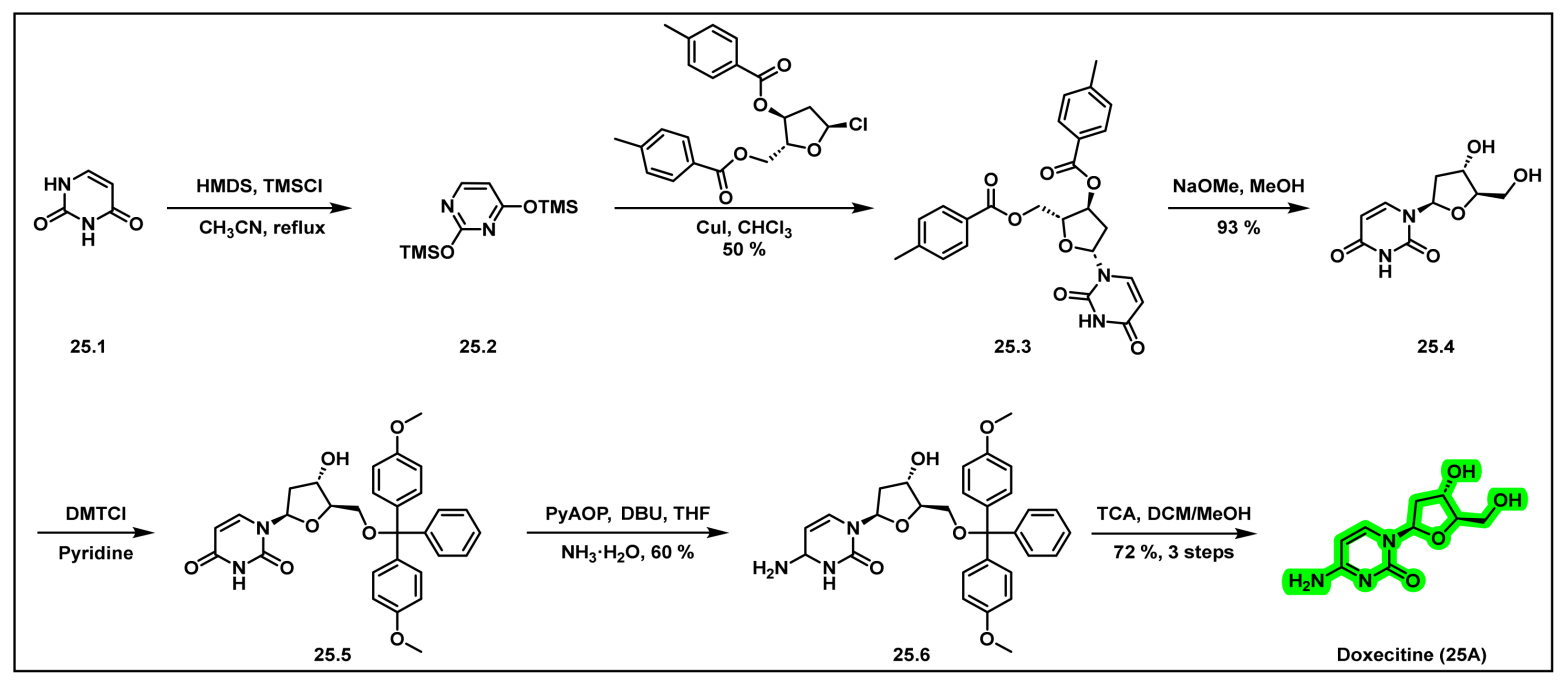
Synthesis of doxecitine.

**Figure 38:**
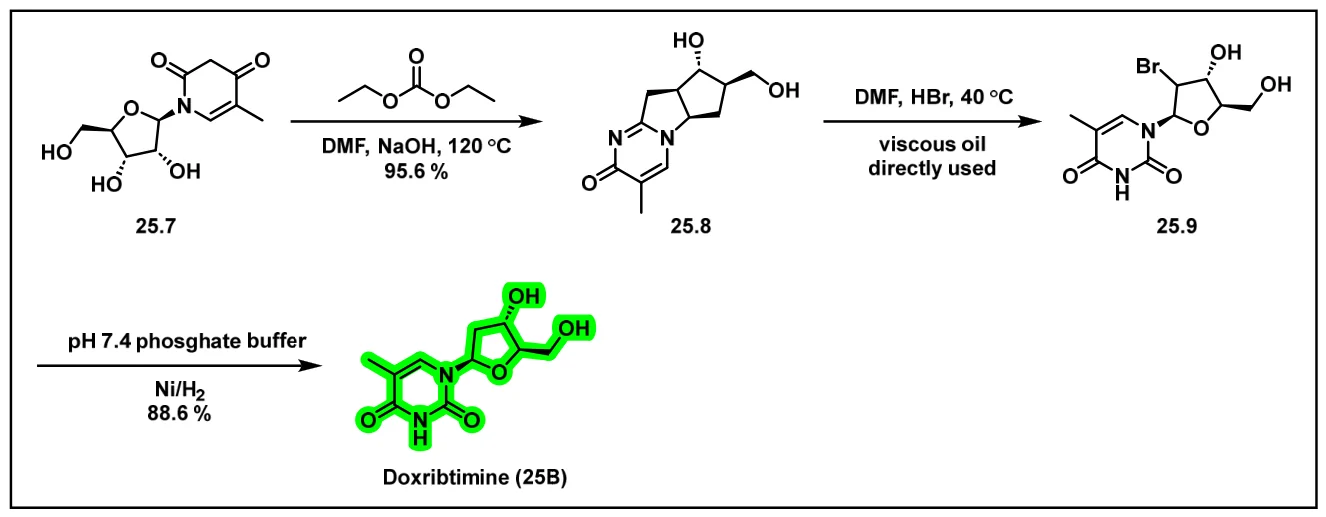
Synthesis of doxribtimine.

**Figure 39:**
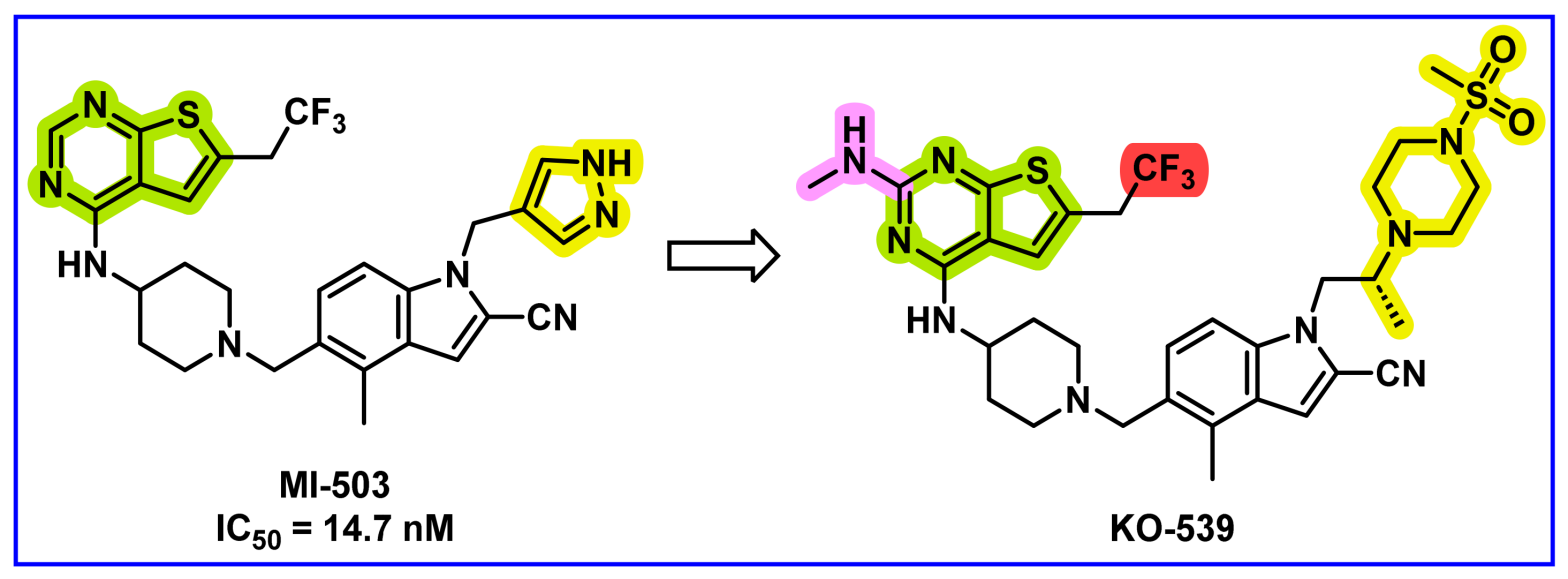
Optimization of ziftomenib.

**Figure 40:**
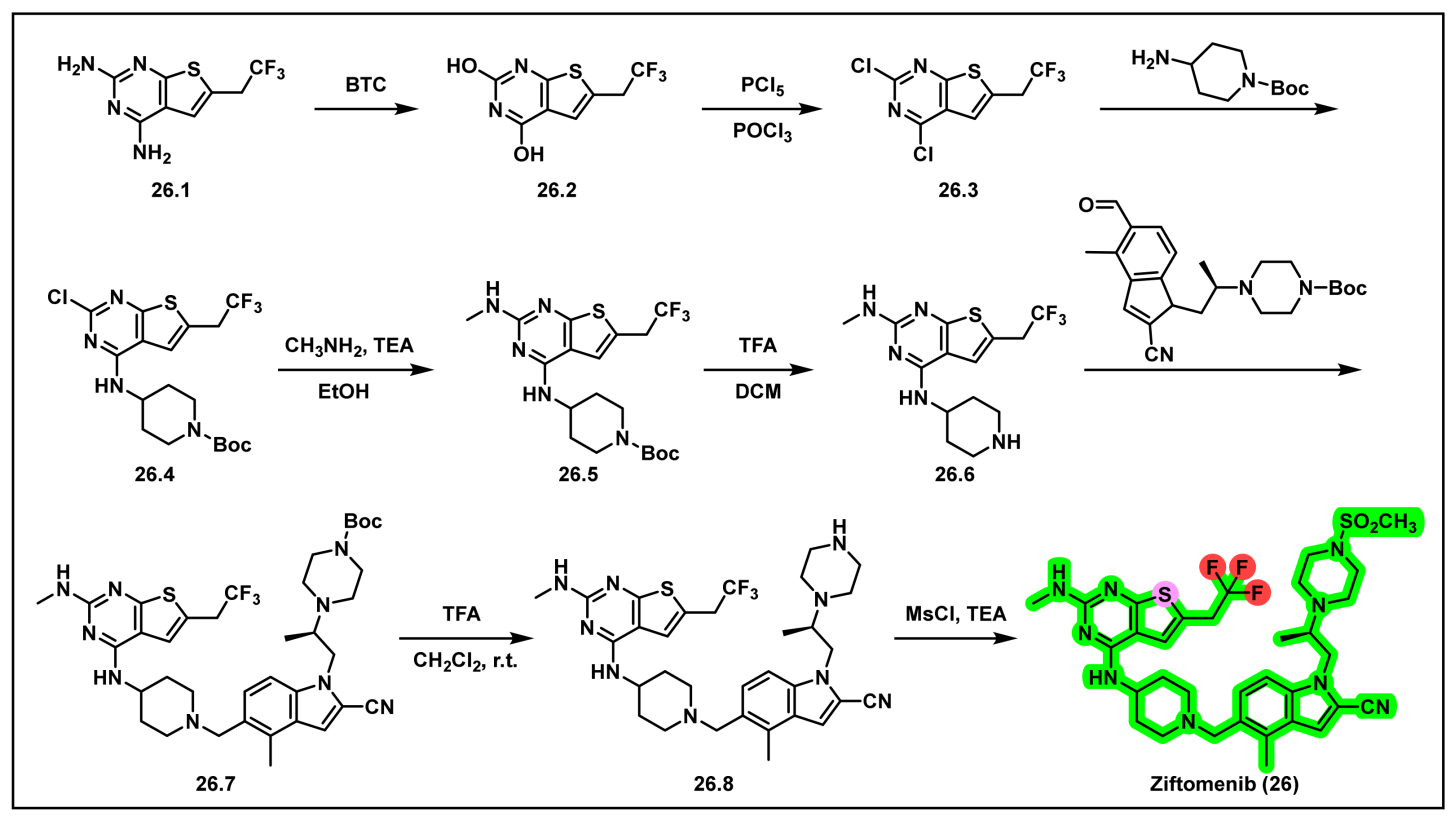
Synthesis of ziftomenib.

**Figure 41:**
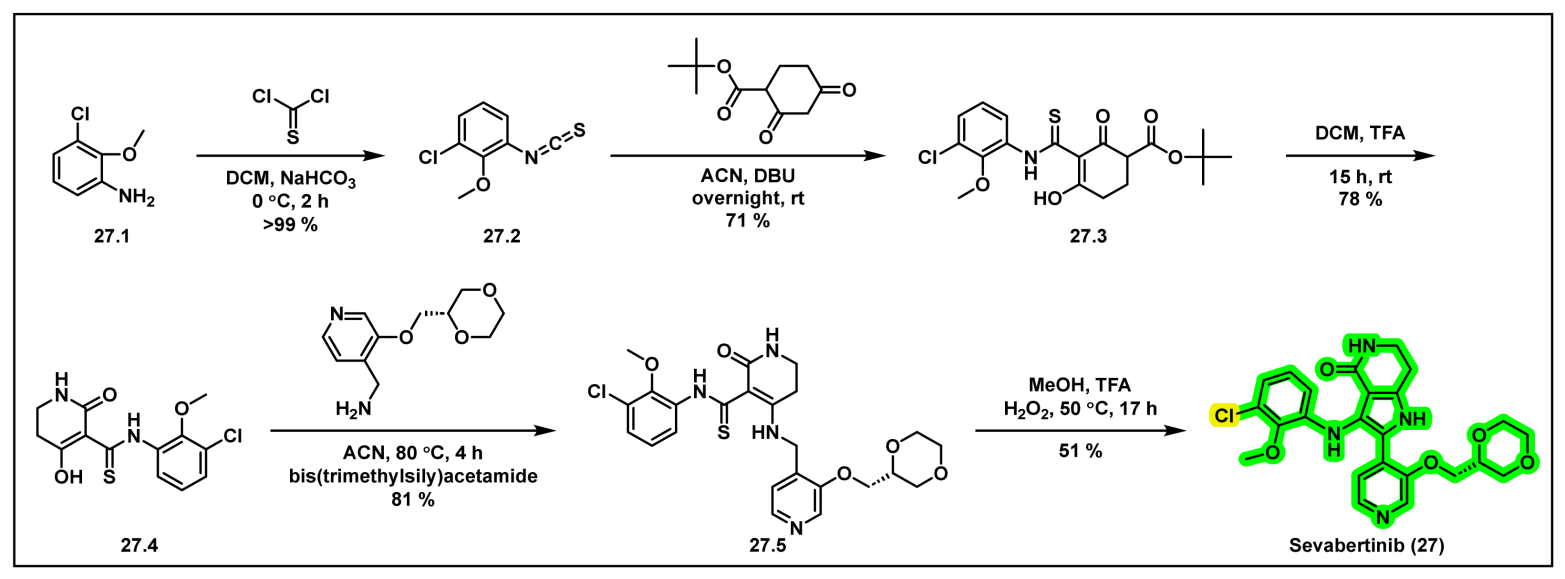
Synthesis of sevabertinib.

**Figure 42:**
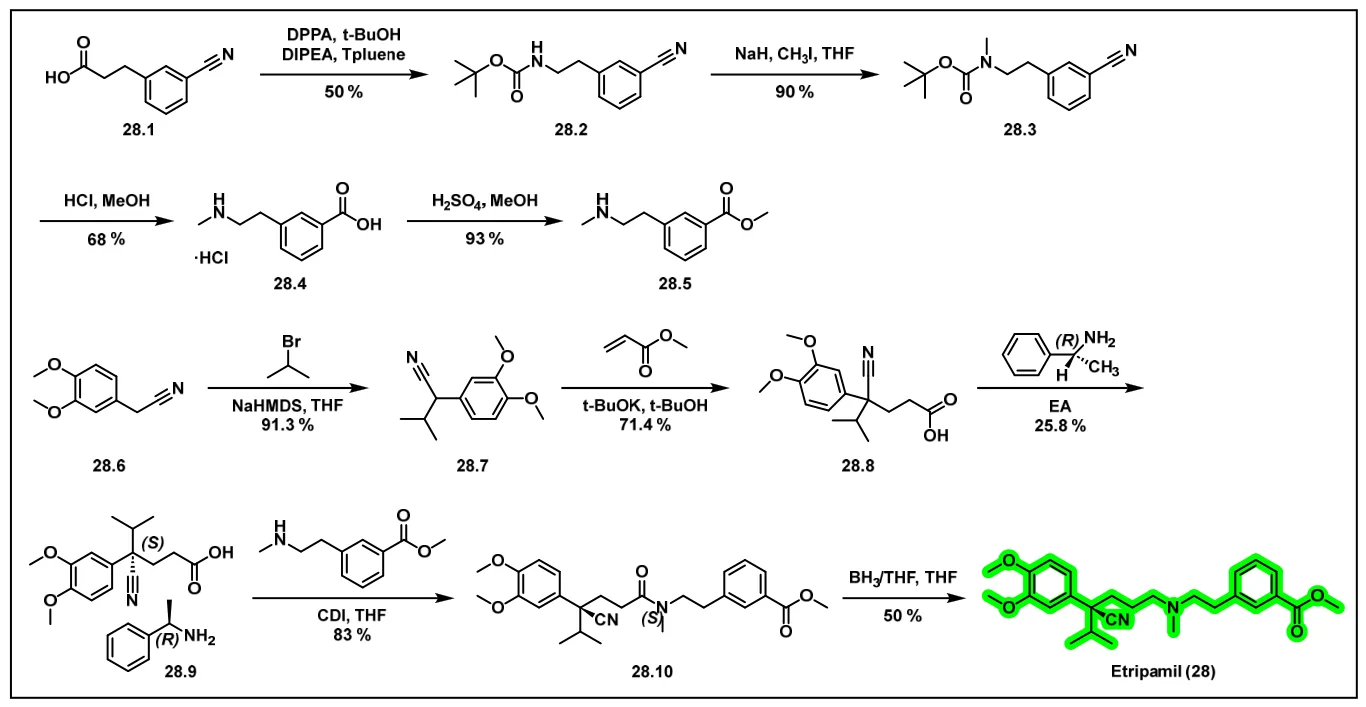
Synthesis of etripamil.

**Figure 43:**
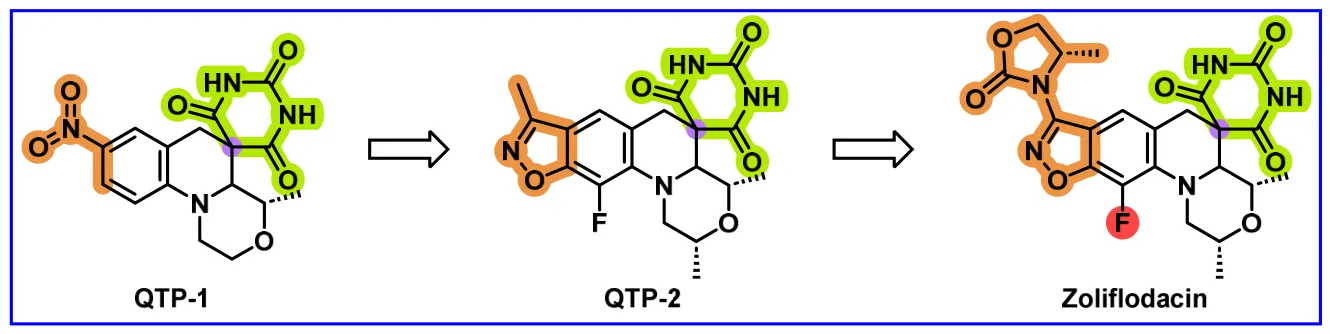
Optimization of zoliflodacin.

**Figure 44:**
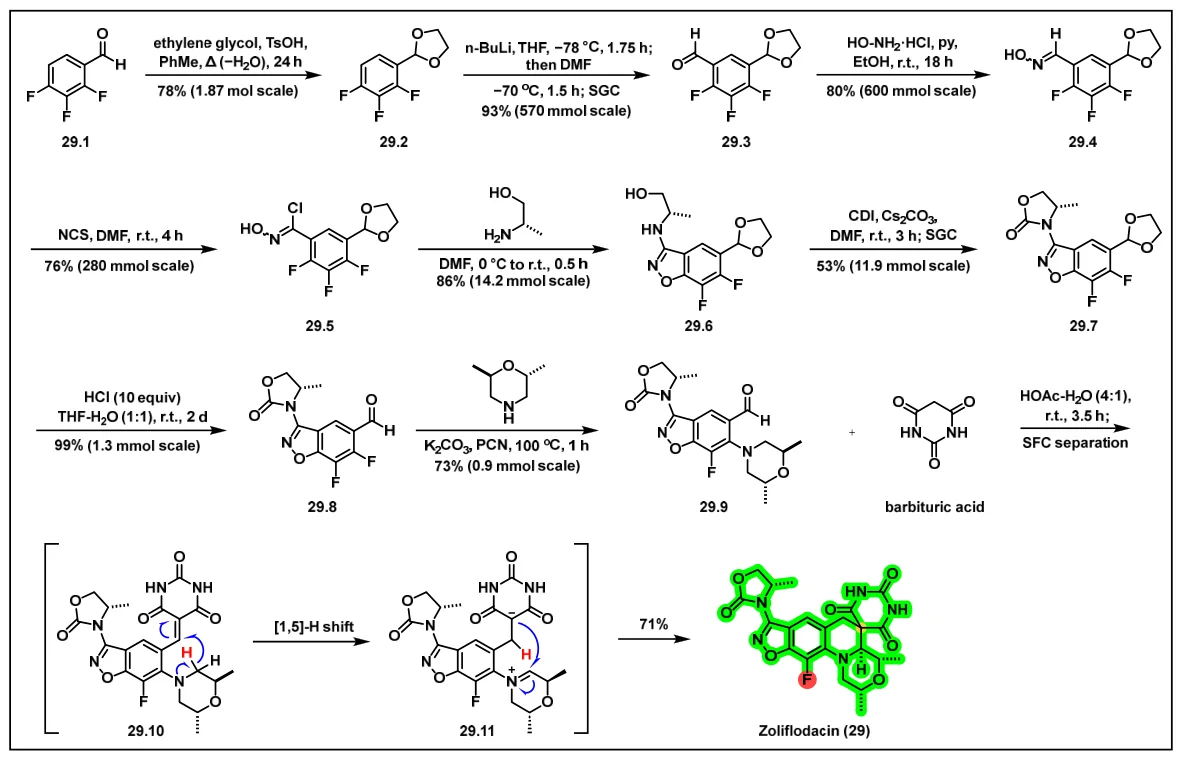
Synthesis of zoliflodacin.

**Figure 45:**
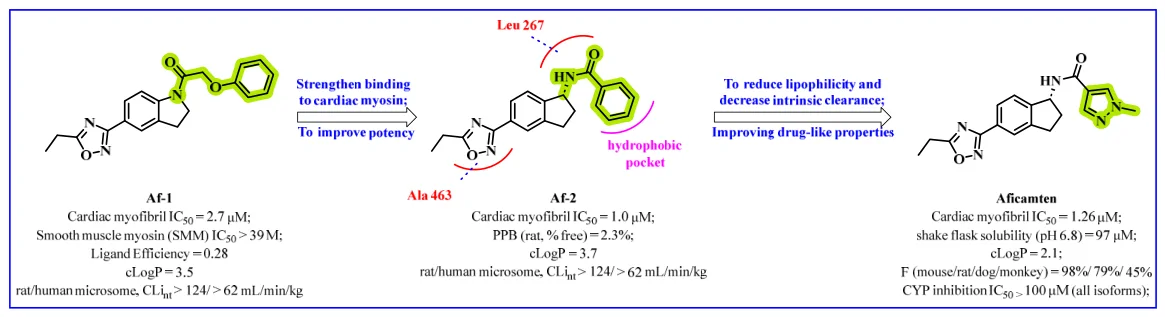
Optimization of aficamten.

**Figure 46:**
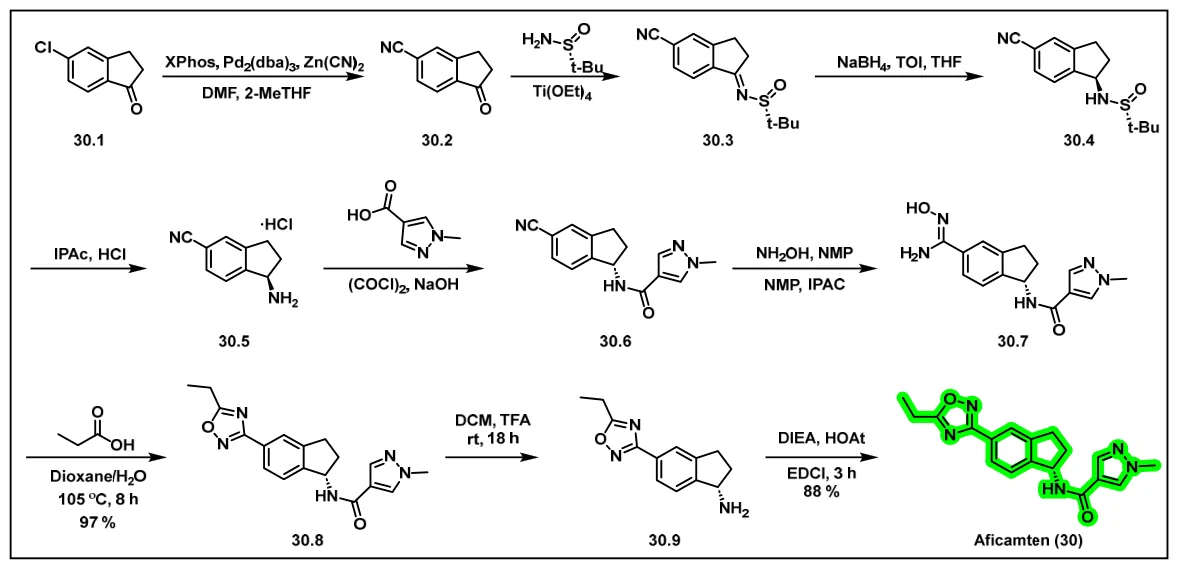
Synthesis of aficamten.

**Figure 47:**
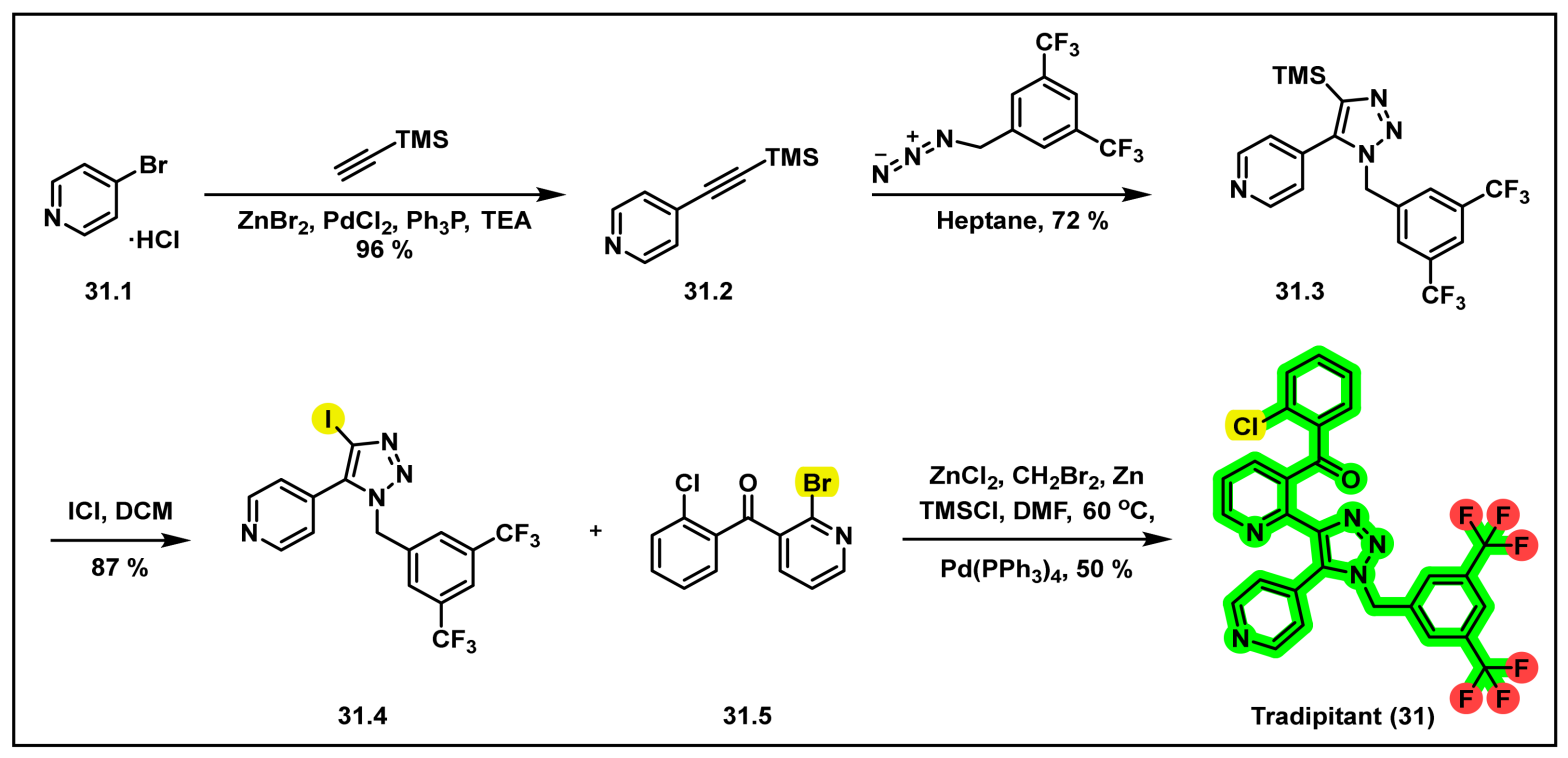
Synthesis of tradipitant.

**Table 1:** Small-molecule drugs approved by the U.S. FDA during 2025 (https://www.fda.gov/drugs/novel-drug-approvals-fda/novel-drug-approvals-2025, accessed on 10 September 2026).

No.	Approval Date	Brand Name (Company)	Active Ingredient (Chemical Structure)	Type	Clinical Trials (Administration)	Indication (Mechanism)	Possible Side Effects
**1**	21 January 2025	Grafapex (Medexus Pharmaceuticals)	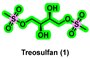	Follow-on	NCT00822393, superior 2-year event-free survival in AML/MDS patients undergoing allogeneic HSCT; (intravenous)	Preparative regimen for hematopoietic stem cell transplantation in cancer; DNA alkylating drug	Abnormal blood chemistry results, gastrointestinal disorders
**2**	30 January 2025	Journavx (Vertex Pharmaceuticals)	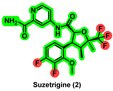	First-in-class	NCT05553366, NCT05463841, Postoperative analgesia for bunionectomy and abdominoplasty; (oral administration)	Moderate-to-severe acute pain in adults; non-opioid analgesic via selective Nav1.8 sodium channel inhibition to block pain signal transmission	Pruritus, muscle spasms, elevated creatine phosphokinase (CPK) levels, and rash
**3**	11 February 2025	Gomekli (SpringWorks Therapeutics)	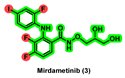	Follow-on	NCT03962543, significant and durable tumor volume reduction; (oral administration)	Plexiform neurofibromas associated with neurofibromatosis type 1 in adults/children ≥ 2 years old; MEK1/2 inhibitor targeting abnormal RAS pathway activation	Rash, diarrhea, nausea, musculoskeletal pain, vomiting, fatigue, headache
**4**	14 February 2025	Romvimza (Ono Pharmaceutical)	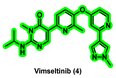	Follow-on	NCT05059262, superior efficacy and favorable safety in tenosynovial giant cell tumor; (oral administration)	Symptomatic tenosynovial giant cell tumor in adults; selective CSF1R kinase inhibitor to suppress tumor growth	Periorbital edema, fatigue, rash, peripheral edema, facial edema, neutropenia, leukopenia, pruritus, and increased alanine aminotransferase
**5**	25 March 2025	Blujepa (GlaxoSmithKline)	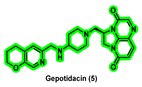	First-in-class	NCT04020341, NCT04187144, significant superiority over Nitrofurantoin; (oral administration)	Uncomplicated urogenital gonorrhea/uncomplicated urinary tract infections; novel triazaacenaphthylene bacterial type II topoisomerase inhibitor	Gastrointestinal in nature, diarrhea, nausea
**6**	2 April 2025	Vanrafia (Novartis)	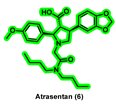	Follow-on	NCT04573478, Proteinuria decreased by 36.1% compared with placebo; (oral administration)	Reduce proteinuria in adults with primary IgA nephropathy; selective endothelin A receptor antagonist	Peripheral edema, anemia, elevated liver transaminases
**7**	8 May 2025	Avmapki Fakzynja Co-Pack (Verastem Oncology)	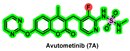 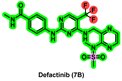	First-in-class	NCT04655270 confirmed overall response rate of 44%; (oral administration)	Recurrent low-grade serous ovarian cancer with KRAS mutations; MEK inhibitor plus FAK inhibitor dual targeted therapy	Elevated blood enzyme levels, nausea, fatigue, abnormal liver function tests, and rash
**8**	28 May 2025	Tryptyr (Alcoltremon)	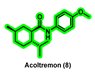	First-in-class	NCT05285644, NCT05360966, higher proportion of patients had ≥10 mm increase (ophthalmic administration)	To treat the signs and symptoms of dry eye disease; TRPM8 thermoreceptor agonist	Instillation site pain
**9**	11 June 2025	Ibtrozi (Nuvation Bio)	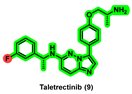	Follow-on	NCT04395677, NCT04919811, confirmed objective response rate (ORR) reached 52% and 62%; (oral administration)	Locally advanced/metastatic ROS1-positive non-small cell lung cancer (NSCLC); selective ROS1/NTRK kinase inhibitor	Diarrhea, nausea, vomiting, dizziness, rash, constipation, and fatigue
**10**	2 July 2025	Zegfrovy (Dizal (Jiangsu) Pharmaceutical)	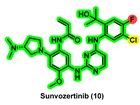	Follow-on	NCT03974022, ORR was 46% and median DOR was 11.1 months; (oral administration)	Metastatic NSCLC with EGFR exon 20 insertion mutations; irreversible EGFR inhibitor targeting exon 20 insertions	Diarrhea, elevated creatine phosphokinase, anemia, elevated lipase, leukopenia, hypokalemia, decreased appetite, and asthenia
**11**	3 July 2025	Ekterly (KalVista Pharmaceuticals)	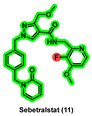	Follow-on	NCT05259917, HAE attack symptom relief was significantly faster; (oral administration)	Hereditary angioedema (HAE) attacks; plasma kallikrein inhibitor to reduce attack frequency	Headache, nasopharyngitis, abdominal pain, fatigue, nausea
**12**	23 July 2025	Anzupgo (LEO Pharma)	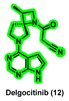	Follow-on	NCT04871711, NCT04872101, met the primary endpoint with statistically significant higher success rates; (topical administration)	Hand eczema; pan-JAK inhibitor targeting inflammatory cytokine pathways	Application site pain, paresthesia, pruritus, erythema, and bacterial skin infections
**13**	28 July 2025	Sephience (PTC Therapeutics)	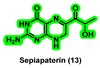	Follow-on	NCT05099640, relative Phe reduction of 64.2% was observed with sapropterin over placebo; (oral administration)	Hyperphenylalaninemia; phenylalanine hydroxylase activator	Diarrhea, headache, abdominal pain, hypophenylalaninemia
**14**	31 July 2025	Vizz (LENZ Therapeutics)	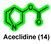	Follow-on	NCT05656027, NCT06045299, improved near vision; (ophthalmic administration)	Presbyopia; muscarinic acetylcholine receptor agonist to improve near vision	Ocular irritation, blurred vision, and headache
**15**	6 August 2025	Modeyso (Jazz Pharmaceuticals)	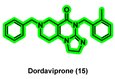	First-in-class	NCT03416530, NCT02525692, NCT03295396, NCT05392374, NCT03134131, DOR was 10.3 months; (oral administration)	To treat diffuse midline glioma harboring an H3 K27M mutation; Caseinolytic protease P protease activator	Fatigue, headache, vomiting, nausea, and musculoskeletal pain
**16**	8 August 2025	Hernexeos (Boehringer Ingelheim)	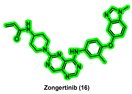	Follow-on	NCT04886804, the BICR-assessed ORR was 76%; (oral administration)	HER2-mutated metastatic NSCLC; pan-HER tyrosine kinase inhibitor	Diarrhea, rash, hepatotoxicity, fatigue, and upper respiratory tract infection.
**17**	12 August 2025	Brinsupri (Insmed Incorporated)	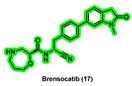	First-in-class	NCT04594369, NCT03218917, reduced annualized pulmonary exacerbation rates; (oral administration)	Non-cystic fibrosis bronchiectasis; reversible dipeptidyl peptidase 1 inhibitor	Upper respiratory tract infection, headache, rash, dry skin, hyperkeratosis, and hypertension
**18**	29 August 2025	Wayrilz (Sanofi)	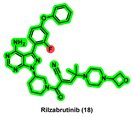	First-in-class	NCT04562766, 23% of patients achieved a durable platelet response; (oral administration)	Immune thrombocytopenia; bruton tyrosine kinase (BTK) kinase inhibitor	Diarrhea, nausea, headache, abdominal pain, and COVID-19.
**19**	19 September 2025	Forzinity (Stealth)	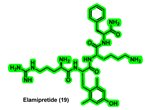	First-in-class	NCT03098797, sustained improvements in knee extensor muscle strength; (subcutaneous administration)	Muscle strength in Barth syndrome; mitochondrial cardiolipin binder	Injection site reactions
**20**	25 September 2025	Palsonify (Crinetics Pharmaceuticals, Inc.)	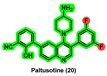	Follow-on	NCT05192382, NCT04837040, rapid onset, reliable biochemical control, and sustained efficacy; (oral administration)	Acromegaly and functional pituitary adenomas; selective somatostatin receptor agonist to suppress growth hormone secretion	Diarrhea, abdominal pain, nausea, decreased appetite, hyperglycemia, palpitations, and gastroenteritis
**21**	25 September 2025	Inluriyo (Eli Lilly and Company)	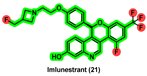	Follow-on	NCT04975308, median progression-free survival of 5.5 months; (oral administration)	ER-positive, HER2-negative, ER1-mutated advanced breast cancer; novel selective estrogen receptor degrader (SERD)	Decreased hemoglobin, musculoskeletal pain
**22**	30 September 2025	Rhapsido (Novartis)	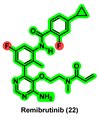	First-in-class	REMIX-1 NCT05030311, REMIX-2 NCT05032157, greater improvements in pruritus and hives; (oral administration)	Chronic spontaneous urticaria in antihistamine-refractory adults; selective BTK inhibitor to suppress mast cell activation	Nasopharyngitis, bleeding, headache, nausea, and abdominal pain
**23**	7 October 2025	Jascayd (Boehringer Ingelheim)	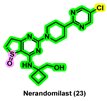	First-in-class	NCT05321069, NCT04419506, significantly attenuated FVC decline; (oral administration)	Idiopathic pulmonary fibrosis in adults; PDE selective inhibitor to reduce lung inflammation and fibrosis	Diarrhea, nausea, vomiting, depression
**24**	24 October 2025	Lynkuet (Bayer)	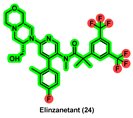	First-in-class	NCT05042362, NCT05099159, reduced the frequency and severity of hot flashes; (oral administration)	Moderate-to-severe menopausal vasomotor symptoms; dual neurokinin-1 (NK1)/neurokinin-3 (NK3) receptor antagonist (non-hormonal therapy)	Mild headache, fatigue
**25**	3 November 2025	Kygevvi (UCB)	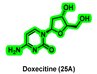 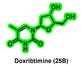	First-in-class	NCT03845712, NCT03701568, NCT05017818 reduced the overall risk of death by approximately 86%; (oral administration)	Thymidine kinase 2 deficiency; pyrimidine nucleoside replacement therapy	Diarrhea, abdominal pain, vomiting, and elevated alanine and aspartate aminotransferase levels
**26**	13 November 2025	Komzifti (Kura Oncology and Kyowa Kirin)	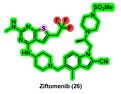	Follow-on	NCT04067336, a 21.4% CR + CRh rate with a 5.0-month median duration of remission; (oral administration)	Relapsed/refractory NPM1-mutated AML in adults; menin–MLL interaction inhibitor	Unspecified organism, hemorrhage, diarrhea, nausea, fatigue, edema
**27**	19 November 2025	Hyrnuo (Bayer)	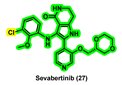	Follow-on	NCT05099172; better efficacy in HER2-mutated NSCLC patients naive to HER2 therapy; (oral administration)	Prior-treated HER2-mutated advanced NSCLC; selective HER2 TKD inhibitor	Diarrhea, rash, paronychia
**28**	12 December 2025	Cardamyst (Milestone)	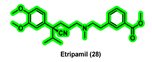	Follow-on	NCT03464019, 64% of patients achieved sinus rhythm restoration within 30 min; (intranasal administration)	Acute conversion of adult PSVT episodes; AV nodal calcium channel blocker	Nasal congestion, rhinorrhea, throat irritation, and epistaxis
**29**	12 December 2025	Nuzolvence (Innoviva Specialty Therapeutics)	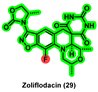	First-in-class	NCT04957257, non-inferior to SOC in achieving microbiological cure at the urogenital site; (oral administration)	Uncomplicated urogenital gonorrhea due to *Neisseria gonorrhoea*; spiropyrimidinetrione bacterial topoisomerase inhibitor	Neutropenia, headache, leukopenia, dizziness, nausea, and diarrhea
**30**	19 December 2025	Myqorzo (Innoviva Specialty Therapeutics)	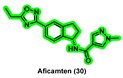	Follow-on	NCT05186818, significantly improved exercise capacity; (oral administration)	Symptomatic obstructive hypertrophic cardiomyopathy (adults); cardiac myosin inhibitor	Hypertension and heart failure risk
**31**	30 December 2025	Nereus (Wanda Pharmaceuticals)	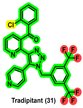	Follow-on	NCT04327661, NCT05903924, significant efficacy in reducing the incidence of vomiting; (oral administration)	Prevention of motion-induced vomiting (adults); NK-1 receptor antagonist	Somnolence, headache, and fatigue

## Data Availability

No new data were created or analyzed in this study. Data sharing is not applicable to this article.
